# Tenascin-N contributes to spinal motor nerve morphogenesis during development

**DOI:** 10.1093/g3journal/jkag259

**Published:** 2026-09-21

**Authors:** Charles G Marcucci, Brianna Peskin Gifford, Marieke Jones, Coleman Blanton, Sarah Kucenas

**Affiliations:** Neuroscience Graduate Program, University of Virginia, Charlottesville, VA 22904, United States; Medical Scientist Training Program, University of Virginia, Charlottesville, VA 22904, United States; Program in Fundamental Neuroscience, University of Virginia, Charlottesville, VA 22904, United States; Program in Fundamental Neuroscience, University of Virginia, Charlottesville, VA 22904, United States; Department of Biology, University of Virginia, Charlottesville, VA 22904, United States; Department of Public Health Sciences, University of Virginia, Charlottesville, VA 22904, United States; Program in Fundamental Neuroscience, University of Virginia, Charlottesville, VA 22904, United States; Department of Biology, University of Virginia, Charlottesville, VA 22904, United States; Neuroscience Graduate Program, University of Virginia, Charlottesville, VA 22904, United States; Medical Scientist Training Program, University of Virginia, Charlottesville, VA 22904, United States; Program in Fundamental Neuroscience, University of Virginia, Charlottesville, VA 22904, United States; Department of Biology, University of Virginia, Charlottesville, VA 22904, United States; University of Virginia Brain Institute, Charlottesville, VA 22904, United States

**Keywords:** Neurodevelopment, Motor nerves, Extracellular matrix, Zebrafish, Neural development, Motor axons

## Abstract

Spinal motor nerves are an integral component of the nervous system whose development requires the coordination of many diverse cell types, including motor neurons, glia, and muscle. Although several molecular mechanisms guiding these interactions are known, many remain to be uncovered. ECM proteins also play a critical role in motor nerve assembly, yet their functions are less understood compared to classical pathfinding and guidance cues. Here, we identify a role for *tenascin-n* (*tnn*), an ECM glycoprotein, in spinal motor nerve development in zebrafish. Using in situ hybridization and immunohistochemistry, we show that *tnn/*Tnn is expressed and localized along vertical myosepta and the border of the ventral neural tube during spinal motor nerve development. To assess its function, we generated a CRISPR/Cas9 mutant allele, *tnn^uva96^*, and performed in vivo imaging and morphological analysis throughout motor nerve development. Loss of *tnn* leads to a subtle and transient trend of increase in ectopic motor axon exit and aberrant motor axon branching in the zebrafish trunk. Our findings reveal a previously unrecognized role for *tnn* in spinal motor nerve assembly and expand our understanding of the diverse molecular contributors to spinal motor nerve development and morphogenesis.

## Introduction

Vertebrate spinal motor nerve development is a tightly orchestrated process that involves coordinated construction by multiple cell types, including neurons, the myotome, and glial cells. Many studies have identified factors crucial for their assembly, including, but not limited to, intrinsic factors, mediators of cell–cell interactions, molecular gradients, and extracellular matrix (ECM) proteins, including collagens, chondroitin sulfate proteoglycans, and Tenascin-C (Bernhardt and Schachner 2000; Schweitzer et al. 2005; Schneider and Granato 2006; Hilario et al. 2010; Guillon et al. 2016; Nemoz-Billet et al. 2024). However, we have yet to uncover all the molecular mechanisms involved and how they cooperate to coordinate motor nerve assembly.

All vertebrates undergo a similar process of spinal motor nerve assembly that involves pioneering motor axons, molecular guidance of a leading growth cone, association of glial cells, and innervation of muscle targets (Bonanomi and Pfaff 2010; Clotman and Tissir 2018). Numerous vertebrate models have provided important insights into elucidating the molecular mechanisms that control these processes, including zebrafish (*Danio rerio*). Zebrafish are an excellent and often used model to study the developmental dynamics of spinal motor nerves as the sequence of events is conserved across vertebrates with the added benefit that they can be easily genetically manipulated and imaged in vivo. In this model, motor nerve assembly begins at approximately 18 h post fertilization (hpf) as the 3 primary motor neurons (MNs) (called rostral primary, middle primary, and caudal primary) extend their axons out of the ventral neural tube at sites called motor exit point transition zones (MEP TZs) (Myers 1985; Myers et al. 1986; Westerfield et al. 1986). Simultaneously, neural crest cells begin streaming ventrally toward exiting motor axons (Raible et al. 1992; Schilling and Kimmel 1994). Following axon extension by the 3 primary MNs, secondary MNs extend axons into the periphery at approximately 22 hpf, increasing in number over time (Pike et al. 1992; Beattie et al. 1997). Between 40 and 72 hpf, motor exit point (MEP) glia and perineurial glia (PG) exit the neural tube and associate with spinal motor nerves, where they will eventually myelinate proximal axon segments and ensheath nerve fascicles, respectively (Kucenas et al. 2008a, 2008b; Binari et al. 2013; Smith et al. 2014; Morris et al. 2017). Around this same stage, neural crest cells associated with nascent motor nerves begin differentiating into Schwann cells and myelinate peripheral motor axons (Raible and Eisen 1994; Schilling and Kimmel 1994; Brösamle and Halpern 2002; Lyons et al. 2005). By 96 hpf, zebrafish larvae have fully functional peripheral motor nerves, including myelinated axons and nerve fascicle ensheathment by PG. In mice, these same events occur. However, the process takes multiple weeks beginning with MN specification and axon extension at E10.5 with the achievement of adult-like movement by P21 (Jessen and Mirsky 2005; Bucchia et al. 2018; Patel et al. 2022).

Many molecules are critical for guiding motor axons out of the central nervous system and directing them to their muscle targets in the periphery, with diffusible attractant or repellant cues and their cognate receptors being the most classically studied, including Slit/Robo, Netrin1/DCC, Semaphorins/Plexins, and Cxcl12/Cxcr4 (Rothberg et al. 1990; Brose et al. 1999; Huber et al. 2005; Lieberam et al. 2005; Palaisa and Granato 2007; Kim et al. 2015, 2017). Less often studied, however, are ECM proteins. A few with known roles include collagens, chondroitin sulfate proteoglycans (CSPGs), and Tenascin-C (Bernhardt and Schachner 2000; Schweitzer et al. 2005; Schneider and Granato 2006; Hilario et al. 2010; Guillon et al. 2016; Nemoz-Billet et al. 2024). Multiple collagen proteins, including ones encoded by *col19a1*, *col18a1*, and *col15a1b*, are crucial for directing peripheral motor axons to their muscle target. For example, loss of *col15a1b* results in defects in primary and secondary motor axon pathfinding and subsequent muscle atrophy (Schneider and Granato 2006; Hilario et al. 2010; Guillon et al. 2016; Nemoz-Billet et al. 2024). Removal of CSPGs via treatment with the enzyme chondroitinase ABC results in aberrant branching of ventral motor axons (Bernhardt and Schachner 2000). Finally, loss of *tenascin-C* (*tnc*) results in aberrant branching of motor axons at a developmental choice point of growing motor axons called the horizontal myoseptum at 33 hpf and increased branching of ventral motor axons at 27 hpf in zebrafish (Schweitzer et al. 2005; Nemoz-Billet et al. 2024). Constituents of the ECM are clearly important contributors to spinal motor nerve development, yet we still have not uncovered all the key players nor elucidated their roles in spinal motor nerve assembly.

In our studies, we sought to investigate whether an understudied tenascin family member, Tenascin-N (Tnn), also played a role in spinal motor nerve development. Tnn, originally named Tnw, was first described by Weber et al. (1998) in zebrafish as the fourth member of the tenascin family, and later named Tnn after the homolog was discovered and named in mice (Neidhardt et al. 2003). In their initial description, Weber et al. (1998) described partially overlapping expression of *tnn* and *tnc* in the developing zebrafish embryo. The initial study in which Tnn was identified in mice demonstrated that it repelled neurite growth of hippocampal cells in culture (Neidhardt et al. 2003). Additionally, Tnn is implicated in stem cell niches, cancer cell signaling, and tumor cell migration and metastasis (Tucker and Degen 2019 review). However, there are no studies investigating Tnn in spinal motor nerve development.

In this study, we identify a transient role for Tnn in spinal motor development. Expression analysis revealed that *tnn* is expressed between 1 and 3 d post fertilization (dpf) in zebrafish embryos and larvae, a critical window during spinal motor nerve assembly. To investigate its function, we generated a putative null mutant allele using CRISPR/Cas9 genome editing and characterized this line through immunohistochemistry (IHC) and in vivo imaging. Loss of *tnn* resulted in transient trends of increased incidence of ectopic spinal motor nerve exits and perturbed axon arborization at 2 dpf. These findings demonstrate that *tnn* contributes to early spinal motor nerve morphogenesis. By focusing on understudied ECM component, our work reveals a previously unrecognized contributor to motor nerve development and underscores the need to further explore the role of ECM in spinal motor nerve assembly.

## Materials and methods

### Zebrafish husbandry

All animal studies were approved by the University of Virginia Institutional Animal Care and Use Committee. Animals were housed at 28 °C at a density of 8 to 10 fish/L and exposed to a 14/10 h light–dark cycle. Previously published zebrafish lines used in this study are included in “Zebrafish transgenic lines.” Newly created zebrafish lines from this study are described in “Generation of stable *tnn* mutant line.” Embryos were produced by pairwise, natural matings and raised in egg water at 28.5 °C. All embryos and larvae were staged according to hpf or dpf (Kimmel et al. 1995) and screened for desired transgenic fluorescence prior to use. Sex was not considered as a variable for experiments because sex is indeterminate in zebrafish prior to 25 dpf (Takahashi 1977).

### Zebrafish transgenic lines

Previously published transgenic lines used in this study include *Tg(olig2:dsred2)^vu19^* (Shin et al. 2003) and *Tg(nkx2.2a:meGFP)^vu17^* (Kucenas et al. 2008b). All transgenic lines described are stable and incorporated into the germline.

### Guide RNA template generation and transcription

We identified *tnn* and *tncb* guide RNAs (gRNAs) using *CRISPRscan* (Moreno-Mateos et al. 2015). We synthesized these templates through PCR amplification with a gene-specific forward primer and the following constant oligonucleotide reverse primer, 5′-*AAAAGCACCGACTCGGTGCCACTTTTTCAAGTTGATAACGGACTAGCCTTATTTTAACTTGCTATTTCTAGCTCTAAAAC*-3′. For the *tnn* gRNA used to create the *tnn^uva96^* allele, we targeted 5′-*CCTCTGGGCCTATAAGGGCTCTC*-3′ in the sixth coding exon and synthesized it using the following forward primer: 5′-*taatacgactcactataGGGAGCCCTTATAGGCCCAGgttttagagctagaaATAGCAAG*-3′. For the alternative *tnn* gRNA, we targeted 5′-*CCACCAGGGGGCGCCTTTCTCCA*-3′ in the 13th coding exon and synthesized it using the following forward primer: 5′-*taatacgactcactataGGGAGAAAGGCGCCCCCTGGgttttagagctagaaATAGCAAG-*3′. For the *tncb* gRNA, we targeted 5′-*TGGTGTCTTGCAGGCCGCTGGGG*-3′ in the eighth exon and synthesized it using the following forward primer 5′-*taatacgactcactataGGGTGTCTTGCAGGCCGCTGgttttagagctagaaATAGCAAG*-3′. To synthesize gRNA templates, we conducted PCR, gel electrophoresis to confirm product size, and purification using QIAquick PCR purification kit (Qiagen). After purification, gRNAs were transcribed using MEGAscript (Ambion) or HiScribe T7 RNA synthesis kit (New England Biolabs) and precipitated via lithium chloride precipitation.

### Generation of stable *tnn* mutant line and crispants

To create a *tnn* mutant allele, *tnn* crispants, and *tnc* crispants, we injected a mixture containing gRNA (200 ng/μL) and Cas9 protein into embryos at the 1-cell stage (or just Cas9 protein alone for Cas9 controls). For the stable *tnn* mutant line, we confirmed insertion and deletion mutations (indels) by extracting genomic DNA via HotSHOT (hot sodium hydroxide and tris), followed by PCR amplification using the following primers: forward primer, 5′*TGACGGAAAACTCTGCGACT*-3′, and reverse primer, 5′-*TCCCTTGAGGAAACGCTTGA*-3′. Purified DNA was then sent for Sanger sequencing through GENEWIZ (Azenta Life Sciences) to confirm successful mutagenesis (Truett et al. 2000). We identified founders and confirmed germline integration by outcrossing F_0_ adults to wild-type (WT) adults and analysis of genomic DNA of F_1_ offspring via Sanger sequencing. We first identified mutant alleles in F_1_ adults via analysis of Sanger sequencing. To confirm suspected mutant alleles, we cloned the PCR product into pCR4-TOPO TA vector (Invitrogen), amplified vectors containing *tnn* mutant alleles in chemically competent *Escherichia coli*, isolated DNA via miniprep (Qiagen), and analyzed Sanger sequencing for indels. We identified F_1_ adults containing a 4 base-pair insertion in the *tnn* gene, and these were raised to establish a stable mutant line for further experimentation (*tnn^uva96^*). All subsequent generations were genotyped as described in “Genotyping and crispant confirmation.”

### Genotyping and crispant confirmation

Genomic DNA was extracted using HotSHOT (hot sodium hydroxide and tris) (Truett et al. 2000). For genotyping of the *tnn^uva96^* allele, PCR amplification was performed using primers listed above, followed by restriction enzyme digestion with Sau96I restriction endonuclease (New England Biolabs). For the digestion, samples were incubated for 1 h at 37 ^°^C followed by heat inactivation for 20 min at 65 ^°^C. The resulting digest products were screened with gel electrophoresis on a 2% agarose gel.

To confirm mosaic mutagenesis in *tnn* and *tncb* crispants, larvae were recovered from imaging dishes after experimentation, and the same procedures were used as described above, except that following PCR amplification, PCR products were purified using QIAquick PCR purification kit (Qiagen) and sent for sequencing through GENEWIZ (Azenta Life Sciences). The resulting chromatograms were analyzed for effective Cas9 cutting, and data acquired from larvae displaying only wild-type sequence was discarded. Primers used for *tnn* alternative gRNA cut-site amplification were as follows: forward primer 5′-*ACAGTATGAGGCGCGTTTTG*-3′ and reverse primer 5′-*ACAAATTCCTCAGCACTCACCA*-3′. Primers used for *tncb* amplification were as follows: forward primer 5′- *TCTGGATTCCCCAAAGCACC*-3′ and reverse primer 5′- *TCTCCAATGTCTGGTTCGCA*-3′.

### In vivo imaging

Embryos were treated with 0.003% PTU at 24 hpf to reduce pigment formation and screened for transgenic fluorescence prior to imaging. For stages before 2 dpf, embryos were manually dechorionated. Embryos and larvae were immobilized prior to imaging using diluted tricaine (MS-222; Syndel/Western Chemical, ordered from The Pond Outlet). Larvae were then mounted laterally in 0.8% low gelling point agarose in 4-well glass bottom 35-mm Petri dishes (Fisher, Greiner Bio-One) and covered with diluted tricaine (concentration varied by age), unless otherwise noted. Imaging was performed on a motorized Zeiss Axio Observer Z1 microscope equipped with a Quorum WaveFX-XI (Quorum Technologies), Andor CSU-W1 Spinning Disc Confocal Microscope, or Andor Dragonfly 200 Spinning Disc Confocal Microscope with iXon Ultra camera with 40×/1.10 objective (Andor/Oxford Instruments). Images were illuminated with 488-nm (green fluorescent protein [GFP], membrane enhanced green fluorescent protein [mEGFP]) and 561-nm (red fluorescent protein [RFP], dsRed) lasers. For still imaging of spinal motor nerves, 3 images were captured starting at the level of the 10th somite and moving posteriorly along the spinal cord. Time-lapse imaging also occurred within this 3-field-window beginning at the 10th somite. Time-lapse length and time-step varied according to the feasibility and needs of the experiment and are stated if relevant. Images were acquired as 60-μm z-series with 2-μm step size unless otherwise stated.

### Cryosectioning

Fixed larvae were mounted in sectioning agar (1.5% agar; Fisher Scientific; 5% sucrose; Sigma; 100-mL ultrapure water) and submerged in 30% sucrose in 1× phosphate-buffered saline (PBS) overnight at 4 °C. Agar blocks were frozen by placing them on a small raft floating in 2methylbutane (Fisher Scientific), the container of which was submerged in a bath of liquid nitrogen. Blocks were sectioned to a thickness of 20 or 40 µm (depending on the experiment) with a cryostat microtome and mounted on microscope slides (VWR). Slides were air dried at room temperature for 3 h and then stored at −20 °C or −80 °C until needed for IHC or in situ hybridization (ISH). Prior to sectioning for IHC or ISH experiments performed on *tnn^uva96^* WT, heterozygote, and mutant clutchmates, larvae were anesthetized in diluted tricaine (MS-222; Syndel/Western Chemical, ordered from The Pond Outlet; concentration varied by age), and the heads were removed with a scalpel. Heads were kept for genotyping, and the trunks were fixed for sectioning.

### Whole-mount IHC

Embryos and larvae (1 to 3 dpf) were dechorionated (if necessary), fixed in 4% paraformaldehyde (PFA; Sigma) for 2 h shaking at room temperature (RT) for all experiments, and used immediately after for antibody staining or stored in 100% methanol for future use. Embryos/larvae were washed in PBSTx (1% Triton X-100, 1× PBS) 3 × 10 min with shaking and then incubated with Proteinase K (diluted in PBSTx to a final concentration of 20 μg/mL) for 20 min at RT. Embryos/larvae were then washed 2 × 5 min with shaking in PBSTx, fixed again in 4% PFA (diluted in PBSTx) with shaking for 20 min at RT, and washed 3 × 5 min with shaking at RT. Embryos/larvae were then blocked in 5% goat serum + 1% DMSO in PBSTx for 1 to 2 h with shaking at RT and then overnight at 4 °C with antibodies in blocking solution. After antibody incubation, embryos/larvae were washed in PBSTx 4 × 10 min with shaking at RT and incubated in a secondary antibody for 2 h with shaking at RT or overnight at 4 °C. Embryos/larvae were washed extensively with PBSTx and stored in 50% glycerol/50% PBS at 4 °C until imaging. The following antibodies were used: rabbit anti-Tenascin-N (1:500; MyBioSource), mouse antiacetylated tubulin (1:10,000; Sigma), Alexa Fluor 488 goat anti-rabbit IgG(H+L) (1:400; Thermo Fisher), and Alexa Fluor 568 goat anti-mouse (1:400; Thermo Fisher). Fish were immobilized and imaged in glass-bottom dishes with microscopes described in “In vivo imaging.”

### IHC on section

Embryos were fixed in 4% PFA for 2 h at RT and sectioned as described in “Cryosectioning.” Sections were rehydrated in 1× PBS for 10 min and blocked with 2% goat serum + 2 mg/mL bovine serum albumin (Fisher Scientific) in PBS for 30 min to 1 h at RT. Sections were incubated in primary antibody overnight at 4 °C. Then, sections were washed continuously with PBS for 45 min followed by secondary antibody incubation for 2 h at RT. The same antibodies and concentrations were used as described in “Whole-mount IHC.”

Slides were stored in the dark until they were imaged using microscopes described in “In vivo imaging.”

### In situ probe synthesis

The *tnn* RNA probe for chromogenic ISH was designed and synthesized in our lab as described in Fontenas and Kucenas (2021). The primers used to generate the *tnn* RNA probe were as follows: forward primer, 5′-*AGGAATCAAGGTGCTGAGCC*-3′, and reverse primer plus T7 sequence, 5′-*TAATACGACTCACTATAGAGCTGGTAAGATGTGGCACC*-3′. For fluorescence in situ hybridization (FISH) experiments, the *tnn* RNA probe (RNAscope probe Dr-tnn-C3) was purchased from Advanced Cell Diagnostics.

### Chromogenic in situ hybridization

Embryos and larvae (1 to 3 dpf) were dechorionated, if necessary, and fixed in 4% PFA for 2 h with shaking at RT and then transferred to 100% MeOH overnight at −20 °C. Chromogenic ISH was performed as described in Hauptmann and Gerster (2000). Images were obtained either using a Zeiss Axio Observer inverted microscope equipped with Zen, using a ×40 oil immersion objective or a Zeiss AxioZoom V16 microscope.

### FISH (RNAscope)

Larvae (1 to 3 dpf) were dechorionated, if necessary, and fixed with 4% PFA for 2 h with shaking at RT, dehydrated in 100% MeOH overnight at −20 °C, and cryosectioned (see “Cryosectioning”). RNAscope was performed as described in Wiltbank et al. (2022). Sections were imaged using equipment described in “In vivo imaging”.

### RT-PCR analysis

RNA was extracted using TRIzol reagent (Invitrogen) from 2 dpf *tnn^uva96^* and AB* embryos. cDNA libraries were generated from the RNA using the high-capacity cDNA reverse transcription kit (Thermo Fisher). Using the cDNA as a template, PCR amplification was performed with the following primers: forward primer, 5′-*GCCAGGGAGGACATGAGAAC*-3′, and reverse primer, 5′*ACAGTCGCAGAGTTTTCCGT*-3′. The resulting PCR products were visualized with gel electrophoresis on a 2.5% agarose gel.

### IHC measurements

Tnn IHC images were processed using Fiji. Tnn signal intensity was measured using the formula for corrected total cell fluorescence (CTCF): CTCF = integrated density – (measured area × mean background intensity). The region of interest integrated density was collected by creating a 60 × 60-μm selection in 1 Z-plane of a stacked histogram surrounding the border of 1 somite. The background mean intensity was collected by creating a 30 × 30-μm selection in the same Z-plane as the region of interest in an area outside of the larvae with only background signal.

### Motor nerve periodicity measurements

In vivo images were processed using Fiji. Images were max projected (and rotated if necessary), and then the multipoint tool was used to mark each motor nerve exit from the spinal cord. After marking all exits, the “measure” function was selected, and the “X” value was exported to excel for each image. Periodicity was calculated by subtracting each “X” value (location of a nerve exit) from the next closest “X” value in the posterior direction within each image. Suspected ectopic exits were confirmed by examining single slices in images stacks in Fiji.

### Imaris filament tracing

Nerve branching was quantified using Imaris (Oxford Instruments) first by cropping images containing multiple nerves and the spinal cord to include only the portion of a single nerve and its constituent branches ventral to the spinal cord. Then, we used the filament tracer module in the Imaris software to semiautomatically trace the nerve and its axonal branches, followed by manual corrections and additions missed by the semiautomatic function. In the resulting statistics, we selected segment terminal points (a count of each axonal ending) as a measurement for axonal branching. After tracing of each nerve was completed, we used the measurement points module in Imaris to measure the distance of the most dorsal point to the most ventral point of the trace mask. This measurement was then included in the statistical analysis of terminal points (see “Quantification and statistical analysis”).

### Quantification and statistical analysis

We conducted quantitative analyses to assess Tnn staining intensity, eye and body measurements, ectopic nerve counts, periodicity of spinal motor nerve exit, and spinal motor nerve branching using Imaris, Fiji, GraphPad Prism, R (version 4.5.2), and R Studio software (version 2023.06.1). Periodicity and nerve branching were measured as described above. The Proportions of ectopic larvae with at least 1 instance of ectopic nerves were compared in GraphPad Prism using Fisher's exact test. Tnn staining intensity was compared in GraphPad Prism using the Mann–Whitney test. Body length and eye diameter measurements were compared in GraphPad Prism using ordinary 1-way ANOVA with Holm–Šídák multiple comparisons test. The width of the 10th somite was compared in GraphPad Prism using 1-way ANOVA with Tukey's post hoc test. Periodicity data was analyzed on the nerve level via linear mixed model (LMM), accounting for random effects of larva and image. The variance of periodicity data was analyzed on the image level via a gamma distribution mixed model, accounting for random effect of larva. Terminal point data was analyzed on the nerve level via a Poisson mixed model, accounting for the random effect of larva and an offset for dorsal-to-ventral length. Dorsal-to-ventral trace length data was analyzed on the nerve level via LMM, accounting for the random effects of larva. Mixed effects models to compare motor nerve periodicity, variance of periodicity, terminal points, and dorsal-to-ventral trace length were generated in R. *P* values for mixed models were generated from post hoc Tukey Honestly Significant Difference (HSD). See Supplementary material for code and mixed model justifications used to generate statistical analyses.

## Results

### *Tnn/*TNN is expressed along vertical myosepta and the ventral border of the spinal cord during zebrafish development

To begin investigating the role of Tnn in spinal motor nerve development, we first sought to investigate its expression pattern in the developing zebrafish embryo between 1 and 3 dpf. We examined both RNA and protein expression, and our data confirmed initial findings reported by Weber et al. (1998). In whole-mount preparations from 1 to 3 dpf, we observed *tnn* and Tnn expression along vertical myosepta bordering each somite (analogous to myotendinous junctions in mammals) (Fig. 1a to f′). At 2 and 3 dpf but not 1 dpf, we also observed Tnn localization adjacent to the ventral boundary of the neural tube, including at MEP TZs, indicated by staining of motor axons with an antiacetylated tubulin antibody (Fig. 1b, b′, d, d′, f, and f′). To better assess the localization of this expression, we cryosectioned 2 and 3 dpf larvae and performed in situ hybridization (chromogenic and fluorescence) and IHC on transverse sections of the trunk. In these sections, we observed *tnn* expression bordering the ventral neural tube, including adjacent to MEP TZs indicated by peripheral *nkx2.2a* expression driven by the *Tg(nkx2.2a:megfp)* transgenic line (Fig. 1g to h′′) as well as Tnn localization along vertical myosepta (Fig. 1g′′ and h′′). From our data, we conclude that *tnn*/Tnn is localized along vertical myosepta and the ventral neural tube, including at MEP TZs.

**Fig. 1.**
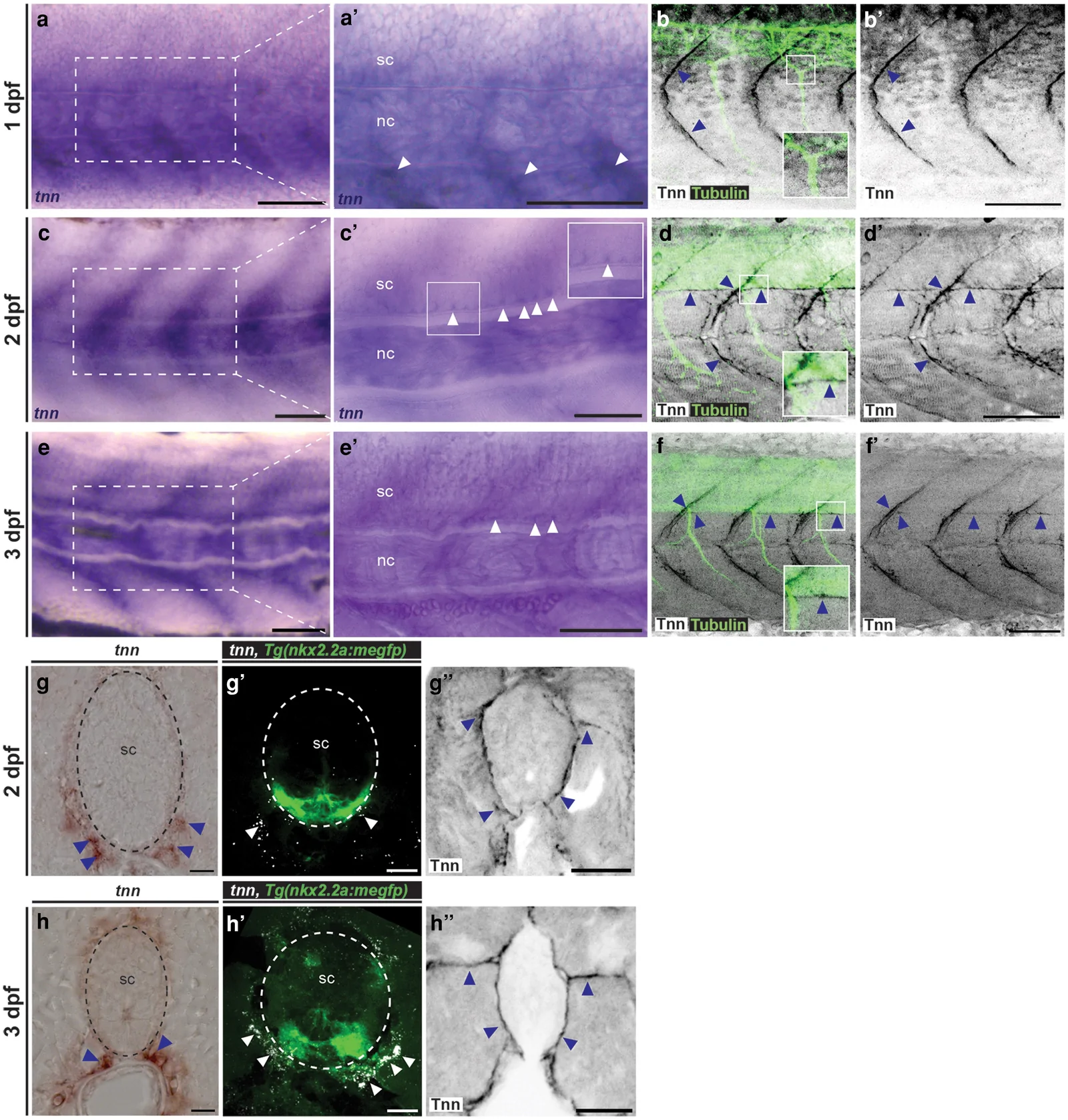
*Tnn/*TNN is expressed along vertical myosepta and the ventral border of the spinal cord during zebrafish development. a, c, and e) Bright-field images from a lateral view of whole-mount chromogenic ISH with enlarged images (a′, c′, and e′) showing *tnn* expression (purple) along vertical myosepta and adjacent to the ventral neural tube (white arrowheads in enlarged images) at 1, 2, and 3 dpf. b and b′, d and d′, and f and f′) Immunofluorescence showing Tnn (black) and tubulin (green) localized along vertical myosepta and ventral neural tube (blue arrowheads) at 1, 2, and 3 dpf (b and b′, d and d′, and f and f′ respectively). Insets in d and f show Tnn at sites of motor axon exit at 2 and 3 dpf, respectively. g) Bright-field image of chromogenic ISH in the transverse section of the trunk showing *tnn* expression (brown) in cells adjacent to the ventral neural tube (arrowheads) at 2 dpf. g′) Fluorescent ISH showing *tnn* expression (white) adjacent to the ventral neural tube (arrowheads) at 2 dpf in the transverse section of *Tg(nkx2.2a:megfp)* larvae. g′′) Immunofluorescence showing Tnn (black) bordering the neural tube and along vertical myosepta (arrowheads) at 2 dpf. h) Bright-field image of chromogenic ISH showing *tnn* expression (brown) in cells adjacent to the ventral neural tube (blue arrowheads) at 3 dpf in transverse section. h′) Fluorescent ISH showing *tnn* expression (white) adjacent to the ventral neural tube (arrowheads) at 3 dpf in the transverse section of *Tg(nkx2.2a:megfp)* larvae. h′′) Immunofluorescence showing Tnn (black) bordering the neural tube and along vertical myosepta (blue arrowheads) at 3 dpf. All images oriented with anterior to the left and dorsal to the top. Scale bars: (a to f) 50 μm; (g to h) 20 μm. sc, spinal cord; nc, notochord.

### Generation of *tnn* mutant allele with CRISPR/Cas9 genome editing

After observing that *tnn* was expressed in a pattern that could influence spinal motor nerve growth, we hypothesized that it may contribute to the development of these structures. To investigate this hypothesis, we created a genetic mutant using CRISPR/Cas9 genome editing (Hwang et al. 2013; Gagnon et al. 2014). We designed a gRNA to target the sixth coding exon of *tnn* and injected it with Cas9 protein into wild-type embryos at the 1-cell stage (Fig. 2a and b). After raising F0 embryos to adulthood, we identified a founder, *tnn^uva96^*, which harbors a 4-base pair insertion in the sixth coding exon that codes for the third of 5-fibronectin type III (FNIII) domains (Fig. 2b and c). This insertion results in a missense (W502L) and nonsense mutation at amino acid positions 502 and 503, respectively, truncating the sequence by 429 amino acids, eliminating 2 FNIII domains and the most C-terminal domain, a fibrinogen-related domain (Fig. 2c and d).

**Fig. 2.**
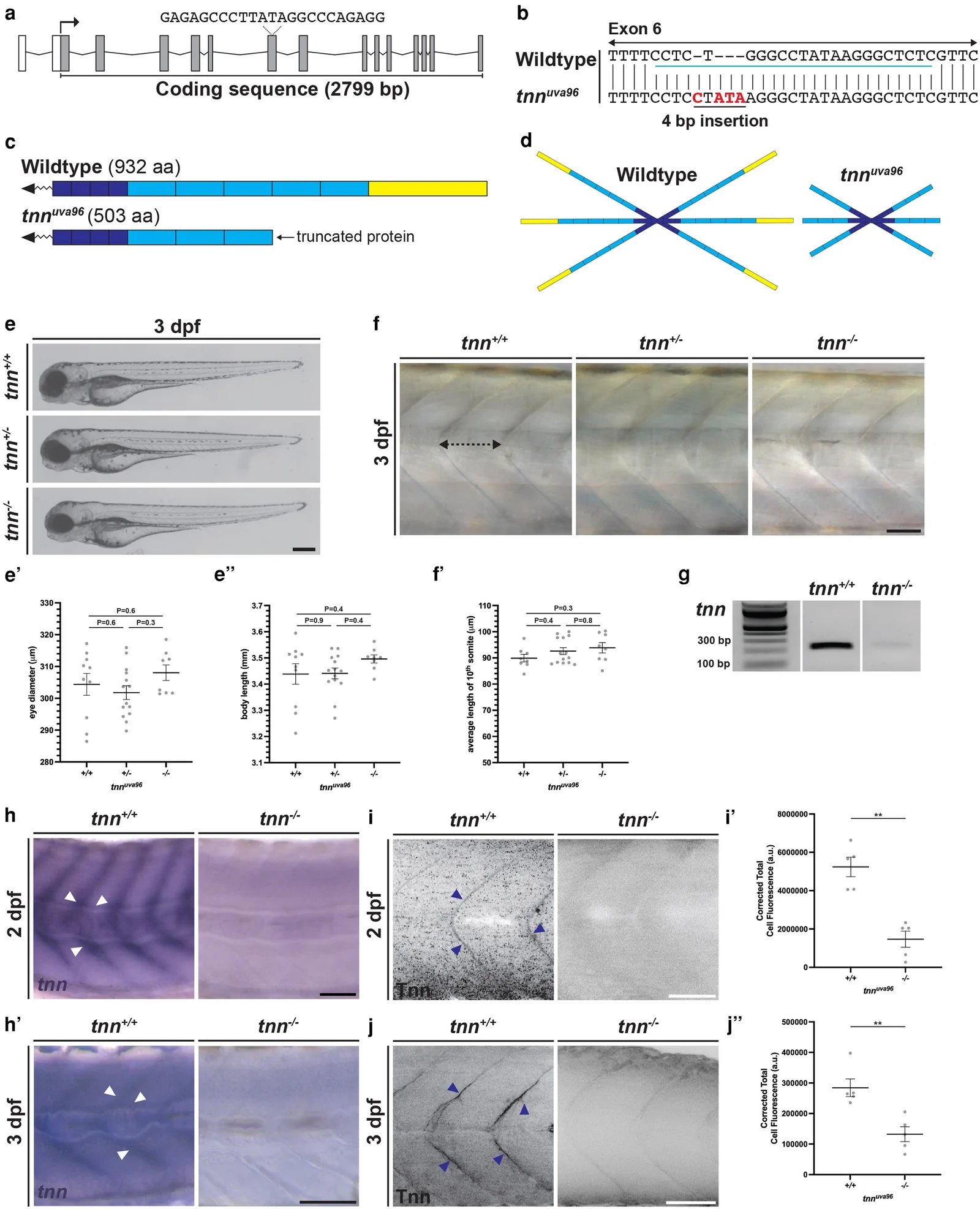
Generation of *tnn* mutant allele using CRISPR/Cas9 genome editing. a) Schematic of the zebrafish *tnn* gene. The coding sequence (gray; 2799 bp) encodes the protein domains represented in (c), and the noncoding sequence is represented in white. Dashed lines above coding exon 6 in schematic indicate the gRNA sequence and target region of the coding sequence. b) Genomic sequences for wild-type *tnn* and mutant *tnn^uva96^* showing a 4-bp insertion within exon 6 of the *tnn* gene. c) Schematic of the Tnn protein made in wild-type (932 aa; top) and predicted protein made in *tnn^uva96^* mutant fish (503 aa; bottom; dark blue, epidermal growth factor-like domains; light blue, FNIII domains; yellow, fibrinogen-related domain). d) Schematic showing that wild-type Tnn assembles into a hexabrachion made up of 2 Tnn trimers with a predicted hexabrachion structure in *tnn^uva96^* mutants. e) In vivo bright-field images of whole 3 dpf *tnn^uva96^* wild-type, heterozygous, and homozygous mutant larvae. e′) Scatter plot of eye diameter at 3 dpf (dot = measurement from 1 larva, wild-type *n* = 10, heterozygous *n* = 14, homozygous *n* = 8). Data compared with ordinary 1-way ANOVA with Holm–Šídák multiple comparisons test. d′′) Scatter plot of body length at 3 dpf (dot = measurement from 1 larva, wild-type *n* = 10, heterozygous *n* = 14, homozygous *n* = 8). Data compared with ordinary 1-way ANOVA with Holm–Šídák multiple comparisons test. f) Differential interference contrast images at the level of the 10th somite in *tnn^uva96^* wild-type, heterozygous, and homozygous mutant larvae at 3 dpf. Black dotted line capped with arrowheads represents the measurements taken in each image for quantification in (f′). f′) Scatter plot of the length of the 10th somite at 3 dpf (dot = measurement from 1 larva, wild-type *n* = 8 heterozygous *n* = 14, homozygous *n* = 8). Data compared with ordinary 1-way ANOVA with Tukey's post hoc test. g) Gel electrophoresis image showing wild-type and *tnn^uva96^* RT-PCR products at 2 dpf. h and h′) Bright-field images of chromogenic ISH showing *tnn* (purple) in wild-type larvae along vertical myosepta and ventral neural tube (arrowheads) and the reduction of *tnn* expression in *tnn^uva96^* homozygous larvae at 2 and 3 dpf. i and j) Immunofluorescence showing Tnn (black) localized along vertical myosepta and ventral neural tube (arrowheads) in wild-type larvae and the reduction of Tnn antibody labeling in *tnn^uva96^* homozygous mutant larvae at 2 and 3 dpf. i′ and j′) Scatter plots of corrected total cell fluorescence comparing Tnn staining intensity between wild-type and homozygous mutant larvae at 2 dpf (i′) and 3 dpf (j′) (dot = measurement from 1 image, wild-type *n* = 5, homozygous *n* = 5). **P < 0.01. Data compared with the Mann–Whitney test. All images oriented with anterior to the left and dorsal to the top. Scale bars: (e) 0.25 mm; (f, h, h′, i, and j) 50 μm.

To begin characterizing *tnn^uva96^* mutant embryos and larvae, we analyzed their general development and morphology. At 3 dpf, we did not observe any gross morphological abnormalities between wild-type, heterozygous, and mutant larvae (Fig. 2e). We also measured body length, eye diameter, and somite width at the level of the 10th somite and saw no significant difference between the genotypes in any of these comparisons (Fig. 2e to f′). Additionally, we observed no differences in swimming behavior (startle and touch response) in developing embryos from 1 to 3 dpf (data not shown), and both heterozygous and homozygous mutant animals survived to adulthood and successfully reproduced.

To determine if our mutant allele was a null allele, we performed RT-PCR, whole-mount RNA in situ hybridization, and IHC. Using all 4 methods, we observed significantly reduced *tnn/*Tnn expression in homozygous mutant larvae at 2 and 3 dpf (Fig. 2g to j′). Specifically, we observed reduced transcript in homozygous mutants with RT-PCR at 2 dpf and in situ hybridization at 2 and 3 dpf (Fig. 2g to h′). Additionally, analysis of IHC images showed a significant decrease in Tnn antibody labeling in homozygous mutant larvae compared to wild-type larvae at both 2 (*P* < 0.01) and 3 dpf (*P* < 0.01) (Fig. 2i to j′). From these data, we conclude that the *tnn^uva96^* allele is a putative null allele that provides us with a line with which to interrogate the functional role of *tnn* in spinal motor nerve assembly.

### *Tnn* contributes to motor axon exit and branching of spinal motor nerves at 2 dpf

Using our newly created mutant allele, *tnn^uva96^*, we investigated the role of *tnn* during spinal motor nerve development. To do this, we used in vivo, confocal imaging in *Tg(olig2:dsred)* larvae where *olig2* regulatory sequences drive expression of DsRed in MNs and axons. In wild-type larvae at 2 dpf, we observed uniform, discrete spacing of motor nerve exits along the anterior–posterior axis of the neural tube (Fig. 3a). In contrast, in *tnn^uva96^* heterozygous and homozygous larvae, we observed a trend toward increased incidence of ectopic motor nerve exit compared to wild-type larvae (Fig. 3a to b). Of all nerves imaged between somites 10 and 22, 6.3% and 5.7% were the result of ectopic exit in heterozygous and homozygous mutant embryos, respectively, compared to 3.0% in wild-type larvae. Furthermore, 50% and 52% of heterozygous and homozygous mutant larvae, respectively, displayed at least 1 ectopic motor nerve exit as compared to 31% of wild-type larvae (Fig. 3b; Fisher's exact test, *P* = 0.4). To further quantify this disorganization, we measured the periodicity, or distance between each nerve exit and its nearest neighbor at 2 dpf (Fig. 3c). Heterozygous and homozygous mutant larvae showed lower (*P* = 0.4 and *P* = 0.8, respectively) average periodicity per larvae compared to wild-type siblings but did not differ significantly (wild-type estimated marginal mean [95% confidence interval {CI}] [emm] = 76.2 μm [72.8, 79.7], heterozygous emm [95% CI] = 73.4 μm [71.1, 75.6], homozygous emm [95% CI] = 74.6 μm [71.3, 77.8]) (Fig. 3c). Additionally, we noticed that larvae with ectopic nerves had smaller average periodicities than those without, creating a wide distribution of these values, so we also statistically compared the variance of periodicity across genotypes. Heterozygous and homozygous mutant larvae showed higher average variance of periodicity (*P* = 0.5 and *P* = 0.3) compared to wild-type siblings (wild-type emm [95% CI] = 36.3 [17.4, 77.0], heterozygous emm [95% CI] = 59.3 [36.2, 97.1], homozygous emm [95% CI] = 75.8, 37.1, 155.2) (Fig. 3c and d).

**Fig. 3.**
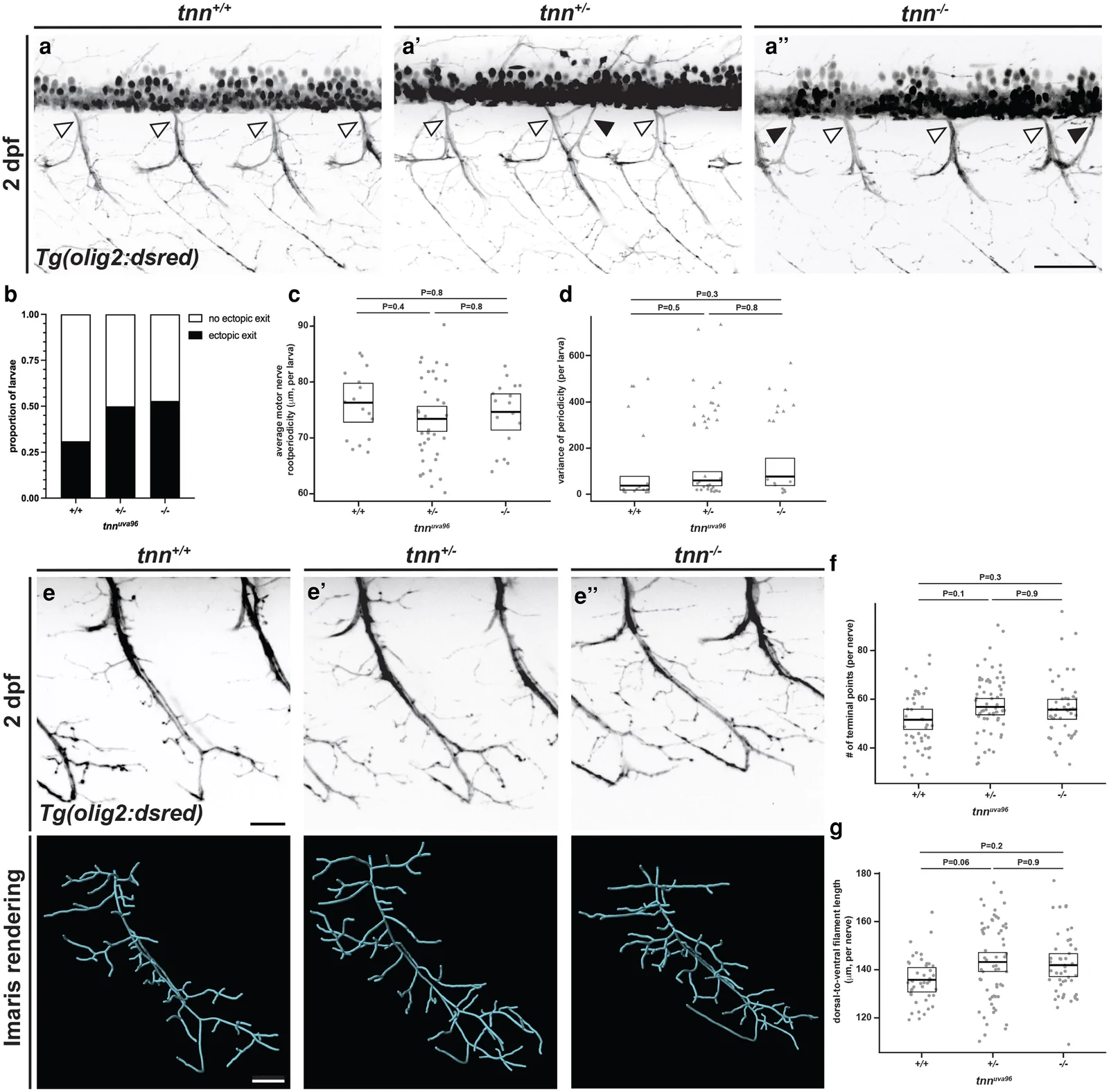
*Tnn* contributes to motor axon exit and branching of spinal motor nerves at 2 dpf. a) Representative images from in vivo, confocal images of laterally mounted *Tg(olig2:dsred)* wild-type (a), *tnn* heterozygous (a′), and *tnn* homozygous (a′′) embryos at 2 dpf showing instances of normally placed ventral motor nerve exits (white arrowheads) and ectopic motor nerve exits (black arrowheads). b) Stacked histogram of proportion of larvae with at least 1 instance of ectopic nerve exit and larvae with no instances of ectopic exit (wild-type *n* = 16, heterozygous *n* = 36, homozygous *n* = 17; Fisher's exact test *P* = 0.4). c) Scatter plot of the average periodicity per larva (measured as the distance between each nerve exit and its nearest neighbor) (dot = 1 larva; wild-type *n* = 16, heterozygous *n* = 36, homozygous *n* = 17). d) Scatter plot of the average variance of periodicity per larva (dot/triangle = 1 larvae; dot = larva with no instances of ectopic exit; triangle = larva with at least 1 instance of ectopic exit; wild-type *n* = 16, heterozygous *n* = 36, homozygous *n* = 17). e) Representative images (top images) from in vivo, confocal images of laterally mounted *Tg(olig2:dsred)* wild-type (e), *tnn* heterozygous (e′), and *tnn* homozygous (e′′) embryos at 2 dpf to show a single nerve selected for tracing with the corresponding Imaris 3D rendering produced from tracing shown below. e) Scatter plot of the number of terminal points per nerve (dot = 1 nerve; wild-type *n* = 43, heterozygous *n* = 62, homozygous *n* = 47). g) Scatter plot of the distance from the most dorsal to the most ventral point of each traced nerve (dot = 1 nerve; wild-type *n* = 43, heterozygous *n* = 62, homozygous *n* = 47). Summary statistics for each scatter plot represent the estimated marginal mean (emm) with 95% CI predicted from the described model. *P* values generated from post hoc Tukey HSD. All images oriented with anterior to the left and dorsal to the top. All images taken between somites 10 and 22. Scale bars: (a′′) 50 μm; (e) 20 μm.

In addition to perturbed motor nerve spacing at 2 dpf, we also observed irregularities in the branching of motor axons in mutant larvae. To thoroughly characterize this, we traced individual nerves using the Imaris imaging filament module (Fig. 3e). From these traces, we used terminal points (a count of each branch ending) as a measurement of arborization and accounted for differences in the length of the primary axon bundle by measuring the length between the dorsal-most and ventral-most portion of each trace rendering (Fig. 3f and g). Heterozygous and homozygous mutant larvae showed a trend of increased terminal points per nerve (*P* = 0.1 and *P* = 0.3) compared to wild-type larvae at 2 dpf but did not differ significantly (wild-type emm [95% CI] = 51.5 [47.5, 55.8], heterozygous emm [95% CI] = 56.7 [53.5, 60.2], homozygous emm [95% CI] = 55.6 [51.7, 59.8] terminal points) (Fig. 3f and g). Notably, we did not trace any of the ectopic nerves as they never grew into full, healthy nerves and would produce statistical outliers. Additionally, comparing traces between larvae with and without instances of ectopic exit does not affect terminal points per nerve (data not shown).

To determine when ectopic exits occurred, we employed the same imaging strategy described above between 24 and 28 hpf and observed initial breaches of ectopically exiting axons (Fig. 4a). At this timepoint, of all nerves imaged, 3.6% and 3.9% were the result of ectopic exit in wild-type and heterozygous mutant embryos, respectively, compared to 0% in homozygous mutant embryos. Furthermore, 21.4% and 36.4% of wild-type and heterozygous mutant embryos, respectively, displayed at least 1 ectopic motor nerve exit as compared to 0% of homozygous embryos (Fig. 4b; Fisher's exact test, *P* = 0.06). Additionally, the average periodicity per embryo did not differ significantly across genotypes but trended higher for homozygous mutants compared to wild-type and heterozygous mutant (*P* = 0.6 and *P* = 0.3) embryos, respectively, at 1 dpf (wild-type emm [95% CI] = 59.9 μm [56.9, 63.0], heterozygous emm [95% CI] = 59.1 μm [57.1, 61.1], homozygous emm [95% CI] = 62.0 μm [58.2, 65.9]) (Fig. 4c). We observed a significant difference across all 3 genotypes in variance of periodicity (wild-type emm [95% CI] = 26.1 [25.9, 26.3], heterozygous emm [95% CI] = 39.4 [39.0, 39.8], homozygous emm [95% CI] = 13.0 [12.8, 13.1]; *P* < 0.0001 for all comparisons) (Fig. 4d). Additionally, using in vivo, time-lapse imaging starting between 24 and 28 hpf, we observed that motor axons actively extend into the periphery at ectopic locations in all genotypes over the course of 16 h, and one of the videos (Supplementary Video 1) demonstrates that growing ectopic nerves can contact neighboring nerves (Supplementary Videos 1 to 3). These findings align with the timing of Tnn localization as the appearance of Tnn along the ventral neural tube does not occur until sometime between 1 and 2 dpf, and Tnn localized to the myosepta is not in a position that could directly contact exiting axons (Fig. 1b and d). Additionally, ectopically exiting axons seem to arise from secondary motor axons because they are *olig2* positive (arise from the pMN domain), they exit after primary motor axon exit, and at 2 dpf, all 3 pioneer pathways from primary motor axons are present. Together, we conclude that Tnn influences the spacing of motor axon exit from the ventral neural tube and arborization of growing motor axons between 1 and 2 dpf.

**Fig. 4.**
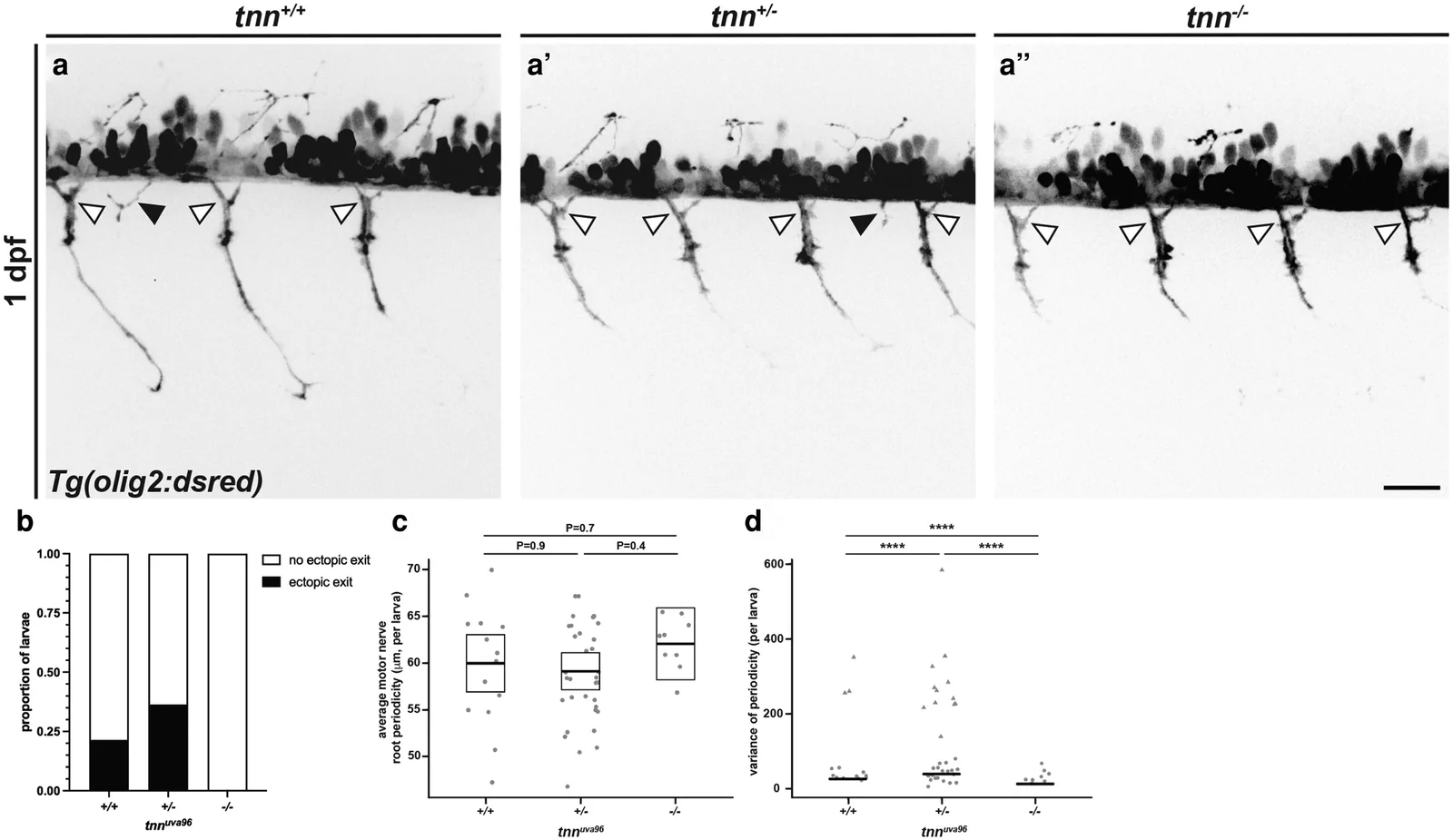
Ectopic exits appear at 1 dpf in *tnn^uva96^* larvae. a) Representative images from in vivo, confocal images of laterally mounted *Tg(olig2:dsred)* wild-type (a), *tnn* heterozygous (a′), and *tnn* homozygous (a′′) embryos at 1 dpf (between 24 and 28 hpf) showing instances of normally placed ventral motor nerve exits (white arrowheads) and ectopic motor nerve exits (black arrowheads). b) Stacked histogram of proportion of embryos with at least 1 instance of ectopic nerve exit and embryos with no instances of ectopic exit (wild-type *n* = 14, heterozygous *n* = 33, homozygous *n* = 10; Fisher's exact test *P* = 0.058). c) Scatter plot of the average periodicity per embryo (measured as the distance between each nerve exit and its nearest neighbor) (dot = 1 embryo, wild-type *n* = 14, heterozygous *n* = 33, homozygous *n* = 10). d) Scatter plot of the average variance of periodicity per embryo (dot/triangle = 1 embryo; dot = embryo with no instances of ectopic exit; triangle = embryo with at least 1 instance of ectopic exit; wild-type *n* = 14, heterozygous *n* = 33, homozygous *n* = 10). ****P < 0.0001. Summary statistics for each scatter plot represent the emm with 95% CI predicted from the described model. *P* values generated from post hoc Tukey HSD. Images oriented with anterior to the left and dorsal to the top. All images taken between somites 10 and 22. Scale bar: (a′′) 50 μm.

To validate our findings we observed at 2 dpf with an alternative method, we used CRISPR/Cas9 mutagenesis to generate mosaic mutants, termed “crispants,” using a different gRNA than we employed to create our *tnn^uva96^* allele. The alternative gRNA we used targeted the 13th coding exon of *tnn* (Supplementary Fig. 1a). We injected *Tg(olig2:dsred)* embryos at the 1-cell stage with the alternative gRNA and Cas9 protein to create crispants and Cas9 only for controls, raised embryos to 2 dpf, and used in vivo imaging as described above to characterize motor nerve exit. We confirmed cutting efficiency in crispant embryos via Sanger sequencing (Supplementary Fig. 1b′′). Of all nerves imaged, 2.5% were the result of ectopic exit in Cas9 controls compared to 5.1% in *tnn* crispants. At the larval level, 17% of control larvae displayed at least 1 ectopic exit per larvae compared to 25% of *tnn* crispant larvae (Supplementary Fig. 1c; Fisher's exact test, *P* = 0.7). Four of five control larvae with ectopic exit displayed only 1 instance of ectopic exit, whereas 2 of 5 crispant larvae with ectopic exit displayed 2 instances and 1 of 5 displayed 3 instances of ectopic exit. We also compared the average periodicity and its variance. *Tnn* crispants had significantly decreased average periodicity (*P* < 0.01) compared to Cas9-injected controls (Cas9-injected emm [95% CI] = 73.9 μm [71.5, 76.4], *tnn* crispant emm [95% CI] = 67.9 μm [65.0, 70.8]) (Supplementary Fig. 1d). Additionally, *tnn* crispants had significantly increased variance of periodicity (*P* < 0.0001) compared to Cas9-injected controls (Cas9-injected emm [95% CI] = 32.6 [32.2, 32.9], *tnn* crispant emm [95% CI] = 48.9 [48.2, 49.7]) (Supplementary Fig. 1e). This alternative method of disrupting *tnn* expression resulted in similar effects on axonal exit at 2 dpf as our *tnn^uva96^* allele, leading us to conclude that these effects are due to perturbation of *tnn* during early motor axon morphogenesis.

### Disruptions to peripheral spinal motor nerve exit and arborization are transient in *tnn^uva96^* homozygous larvae

To determine whether the disruptions to nerve exit and branching persisted as spinal motor nerve development progressed, we repeated in vivo imaging in 3 dpf *tnn^uva96^;Tg(olig2:dsred)* larvae (Fig. 5a). Interestingly, of all nerves imaged, 6.9% were the result of ectopic exit in heterozygous mutant larvae, compared to 4.5% and 4.3% for wild-type and homozygous mutant larvae, respectively. Furthermore, 56% of heterozygous mutant larvae displayed at least 1 ectopic nerve exit, compared to 36% and 29% of wild-type and homozygous mutant larvae (Fig. 5b; Fisher's exact test, *P* = 0.2). Examining periodicity at 3 dpf, we observed no significant difference between genotypes in average periodicity per larvae (wild-type emm [95% CI] = 79.2 μm [75.2, 83.1], heterozygous emm [95% CI] = 79.3 μm [76.5, 82.2], homozygous emm [95% CI] = 80.5 μm [76.9, 84.1]) (Fig. 5c). We did, however, observe significant differences in the variance of periodicity across all 3 genotypes (wild-type emm [95% CI] = 42.7 [42.3, 43.1], heterozygous emm [95% CI] = 55.5 [54.8, 56.2], homozygous emm [95% CI] = 37.7 [37.2, 38.2]; *P* < 0.0001 for all comparisons) (Fig. 5d). We also performed the same motor nerve tracing to characterize branching of ventral motor nerves at 3 dpf (Fig. 5e to g). Heterozygous and homozygous mutant larvae showed a trend of decreased motor axon arborization compared to wild-type larvae (*P* = 0.2 and *P* = 0.3) at 3 dpf, although they were not significantly different (wild-type emm [95% CI] = 109 [102.4, 117], heterozygous emm [95% CI] = 102 [97.2, 107], homozygous emm [95% CI] = 102 [95.8, 109] terminal points) (Fig. 5f and g). Taken together, we did not observe the persistence of the trends in ectopic axon exit and motor axon arborization at 3 dpf in homozygous mutant larvae, while in heterozygous mutant larvae, the trend observed at 2 dpf for incidence of ectopic exit and variance of periodicity did persist at 3 dpf. These findings led us to hypothesize that genetic compensation induced by loss of 2 copies of *tnn* may be contributing to these differences seen at 2 vs 3 dpf.

**Fig. 5.**
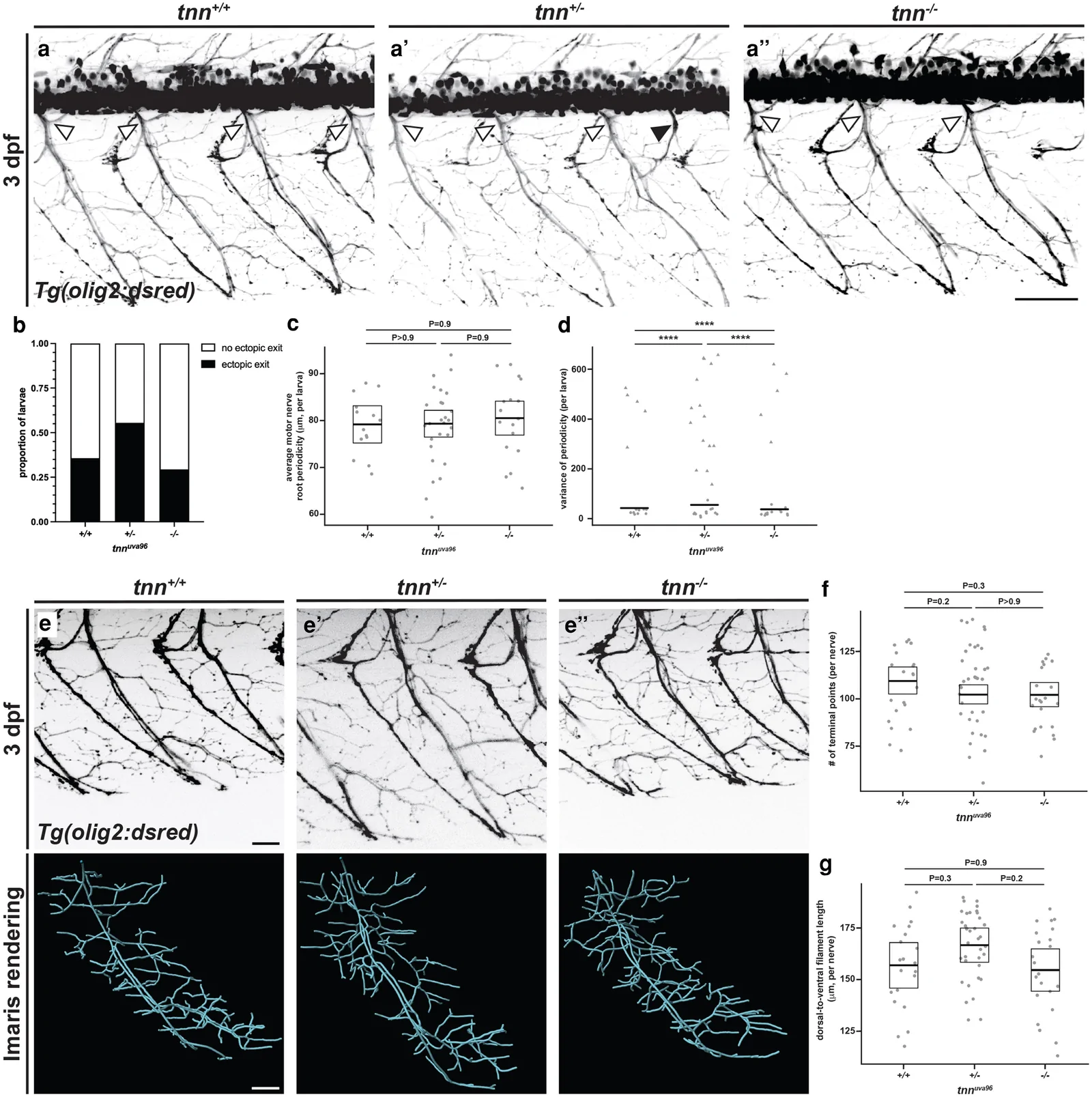
Disruptions to peripheral spinal motor nerve exit and arborization are transient in *tnn^uva96^* homozygous larvae. a) Representative images from in vivo, confocal images of laterally mounted *Tg(olig2:dsred)* wild-type (a), *tnn* heterozygous (a′), and *tnn* homozygous (a′′) larvae at 3 dpf showing instances of normally placed ventral motor nerve exits (white arrowheads) and ectopic motor nerve exits (black arrowheads). b) Stacked histogram of proportion of larvae with at least 1 instance of ectopic nerve exit and larvae with no instances of ectopic exit (wild-type: *n* = 14, heterozygous: *n* = 27, homozygous: *n* = 17; Fisher's exact test *P* = 0.2). c) Scatter plot of the average periodicity per larva measured as the distance between each nerve exit and its nearest neighbor (dot = 1 larva; wild-type *n* = 14, heterozygous *n* = 27, homozygous *n* = 17). d) Scatter plot of the average variance of periodicity per larva (dot/triangle = 1 larva; dot = larva with no instances of ectopic exit; triangle = larva with at least 1 instance of ectopic exit; wild-type *n* = 14, heterozygous *n* = 27, homozygous *n* = 17). ****P < 0.0001. e) Representative images (top images) from in vivo confocal imaging of laterally mounted *Tg(olig2:dsred)* wild-type (e), *tnn* heterozygous (e′), and *tnn* homozygous (e′′) larvae at 3 dpf enlarged to show a single nerve selected for tracing with the corresponding Imaris 3D rendering produced from tracing shown below. f) Scatter plot of the number of terminal points per nerve (dot = 1 nerve; wild-type *n* = 21, heterozygous *n* = 36, homozygous *n* = 22). g) Scatter plot of the distance from the most dorsal point to the most ventral point of each traced nerve (dot = 1 nerve; wild-type *n* = 21, heterozygous *n* = 36, homozygous *n* = 22). Summary statistics for each scatter plot represent the emm with 95% CI predicted from the described model. *P* values generated from post hoc Tukey HSD. All images oriented with anterior to the left and dorsal to the top. All images taken between somites 10 and 22. Scale bars: (a′′) 50 μm; (e) 20 μm.

### *Tnc* and *tnn* control distinct aspects of spinal motor nerve development

Because *tnc* is known to play a role in spinal motor nerve development in zebrafish, we hypothesized that *tnc* and *tnn* may play synergistic roles in this process. Therefore, we designed and created a gRNA to target *tnc* to create *tnc* crispants in our *tnn* mutant larvae to test whether loss of both genes would result in persistence of 2 dpf trends in ectopic exit and arborization in homozygous mutant larvae at 3 dpf. To do this, we designed a gRNA targeting the eighth exon of the *tncb* gene (Fig. 6a to b). We injected this gRNA with Cas9 protein into *tnn* heterozygous and homozygous mutant embryos with *Tg(olig2:dsred)* in the background at the 1-cell stage. We raised these embryos to 3 dpf and performed the same in vivo imaging protocol described above, followed by confirmation of cutting efficiency by Sanger sequencing (Fig. 6c). Of all nerves imaged, 7.1% were the result of ectopic exit in *tnn* heterozygous/*tnc* crispant larvae compared to 2.0% in *tnn* homozygous/*tnc* crispant larvae. Furthermore, 66.7% of *tnn* heterozygous/*tnc* crispant larvae displayed at least 1 ectopic nerve exit compared to 22.2% of *tnn* homozygous/*tnc* crispant larvae (Fig. 6d; Fisher's exact test, *P* = 0.08). The average periodicity in *tnn* heterozygous/*tnc* crispant larvae trended lower compared to *tnn* homozygous/*tnc* crispant larvae but was not significantly different (*P* = 0.1) (*tnn* heterozygous/*tnc* crispant emm [95% CI] = 75.2 [71.0, 79.4], *tnn* homozygous/*tnc* crispant emm [95% CI] = 80.5 [75.7, 85.4]) (Fig. 6e). The variance of periodicity was significantly increased in *tnn* heterozygous/*tnc* crispants compared to *tnn* homozygous/*tnc* crispants (*P* < 0.0001) (*tnn* heterozygous/*tnc* crispant emm [95% CI] = 95.0 [94.3, 95.8] *tnn* homozygous/*tnc* crispant emm [95% CI] = 40.1 [39.7, 40.6]) (Fig. 6f). These findings replicate the same trends we observed when comparing uninjected heterozygous and homozygous *tnn* mutants at 3 dpf, leading us to conclude that *tnc* does not contribute to motor axon exit at this timepoint via genetic compensation in *tnn* homozygous mutants, though it may be contributing to this phenotype independently.

**Fig. 6.**
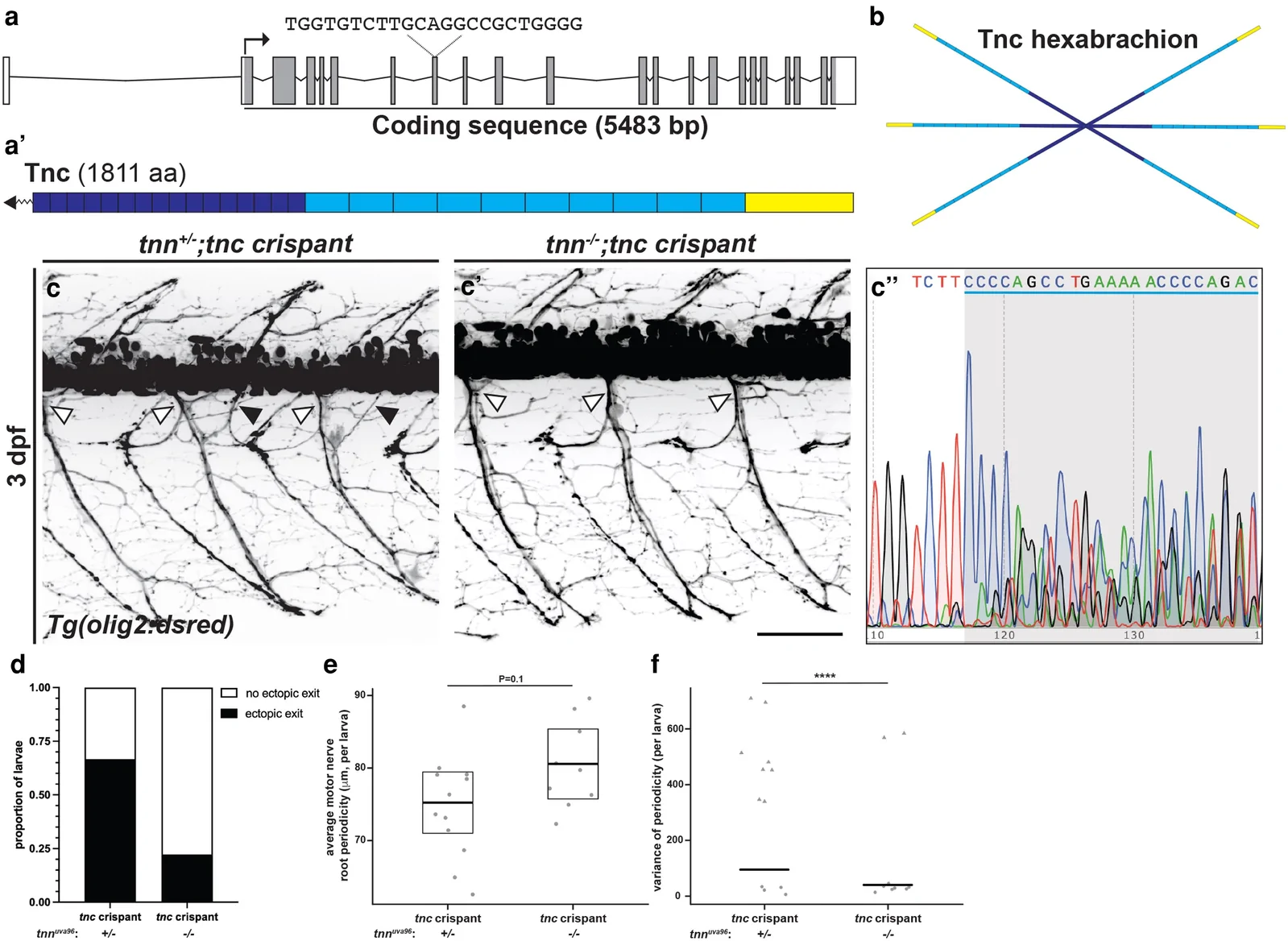
*Tnc* and *tnn* control distinct aspects of spinal motor nerve development. a) Schematic of the zebrafish *tncb* gene. The coding sequence (gray; 5,483 bp) encodes the protein domains represented in (a′), and the noncoding sequence is represented in white. Dashed lines above exon 8 in schematic indicate the gRNA sequence and target region of the coding sequence. a′) Schematic of the Tnc protein made in wild-type (1,811 aa; dark blue, epidermal growth factor-like domains; light blue, FNIII domains; yellow, fibrinogen-related domain). b) Schematic showing that wild-type Tnc assembles into a hexabrachion made up of 2 Tnc trimers. c) Representative images from in vivo, confocal images of laterally mounted *Tg(olig2:dsred) tnn* heterozygous/*tnc* crispant (c) and *tnn* homozygous/*tnc* crispant larvae (c′) larvae at 3 dpf showing instances of normally placed ventral motor nerve exits (white arrowheads) and ectopic motor nerve exits (black arrowheads). c′′) Example chromatogram from *tnc* crispant larvae showing multiple overlapping peaks after the target sequence of the gRNA (blue underline, gray shaded region) representing multiple different alleles generated from gRNA/Cas9 activity. d) Stacked histogram of proportion of larvae with at least 1 instance of ectopic nerve exit and larvae with no instances of ectopic exit (*tnn* heterozygous/*tnc* crispant *n* = 12, *tnn* homozygous/*tnc* crispant *n* = 9; Fisher's exact test *P* = 0.081). e) Scatter plot of the average periodicity per larva (measured as the distance between each nerve exit and its nearest neighbor) (dot = 1 larva; *tnn* heterozygous/*tnc* crispant *n* = 12, *tnn* homozygous, *tnc* crispant *n* = 9). f) Scatter plot of the average variance of periodicity per larva (dot/triangle = 1 larva; dot = larva with no instances of ectopic exit; triangle = larva with at least 1 instance of ectopic exit; *tnn* heterozygous/*tnc* crispant *n* = 12, *tnn* homozygous/*tnc* crispant *n* = 9). ****P < 0.0001. Summary statistics for each scatter plot represent the emm with 95% CI predicted from the described model. *P* values generated from post hoc Tukey HSD. Images oriented with anterior to the left and dorsal to the top. Images taken between somites 10 and 22. Scale bar: (c′) 50 μm.Chromatogram generated from SnapGene.

To further explore whether *tnc* affects motor axon exit dynamics in *tnn* heterozygous and homozygous mutants, we performed the same injection of our *tnc* gRNA with Cas9 protein as described above and imaged the larvae at 2 dpf (Supplementary Fig. 2a). Of all nerves imaged, 4.5% were the result of ectopic exit in *tnn* heterozygous*/tnc* crispant larvae compared to 1.5% in *tnn* homozygous/*tnc* crispant larvae. Additionally, 38% of *tnn* heterozygous/*tnc* crispant larvae displayed at least 1 ectopic nerve exit compared to 12.5% of *tnn* homozygous/*tnc* crispant larvae (Supplementary Fig. 2b; Fisher's exact test, *P* = 0.3). There were no differences in average periodicity between *tnn* heterozygous/*tnc* crispant and *tnn* homozygous/*tnc* crispant larvae (*P* = 0.7) (*tnn* heterozygous/*tnc* crispant emm [95% CI] = 67.9 [64.3, 71.5], *tnn* homozygous/*tnc* crispant emm [95% CI] = 68.9 [64.3, 73.5]) (Supplementary Fig. 2c), and the variance of periodicity was higher in *tnn* heterozygous/*tnc* crispants compared to *tnn* homozygous/*tnc* crispants, but did not differ significantly (*P* = 0.2) (*tnn* heterozygous/*tnc* crispant emm [95% CI] = 39.2 [18.02, 85.3] *tnn* homozygous/*tnc* crispant emm [95% CI] = 18.9 [7.14, 50.3]) (Supplementary Fig. 2d). Therefore, we conclude that disrupting *tnc* expression in *tnn* homozygous mutants does not worsen axonal exit phenotypes we observed at either 2 or 3 dpf.

### CRISPR/Cas9 mosaic mutagenesis of *tnn* does not affect spinal motor nerve exit or motor axon arborization

Previous studies demonstrate that in stable zebrafish mutant lines, models of known disease-causing mutations sometimes do not recapitulate previously reported phenotypes due to genetic compensation (Kok et al. 2015; Rossi et al. 2015). However, transient knockdown approaches or creating mosaic mutants via injection of a gRNA and Cas9 mRNA (or protein) at the 1-cell stage can mitigate the effects of genetic compensation, and this method can result in gene-specific phenotypes (Moreno et al. 2018; She, et al. 2019; Buglo, et al. 2020). To investigate whether broad genetic compensation of *tnn* contributed to the phenotypic differences we observed at 2 vs 3 dpf in *tnn^uva96^* mutant larvae, we utilized the CRISPR/Cas9 transient knockdown approach again. We injected the *tnn-*targeted gRNA that we used to create the *tnn^uva96^* allele and Cas9 protein (or Cas9 alone as a control) into *Tg(olig2:dsred)* embryos at the 1-cell stage creating mosaic mutants. We raised *tnn* crispant and Cas9-injected embryos to 3 dpf and used in vivo imaging to characterize motor nerve exit and branching, followed by Sanger sequencing to confirm cutting efficiency (Fig. 7a). In these studies, we did not observe any statistical differences between Cas9-injected and *tnn* crispant larvae in proportion of ectopic nerves (Cas9-injected = 2.6%; *tnn* crispant = 1.7%) nor proportion of larvae with at least 1 ectopic nerve exit (Cas9-injected = 27.8%; *tnn* crispant = 21.1%; Fisher's exact test, *P* = 0.7) (Fig. 7b). Additionally, there were no differences in average periodicity per larvae (*P* = 0.8) (Cas9-injected emm [95% CI] = 81.5 μm [79.1, 84.0], *tnn* crispant emm [95% CI] = 81.1 μm [78.7, 83.5]), but the variance of periodicity was significantly increased in *tnn* crispants (Cas9-injected emm [95% CI] = 34.4 [34.0, 34.8], *tnn* crispant emm [95% CI] = 43.1 [42.4, 43.9]; *P* < 0.0001) (Fig. 7c and d). Additionally, we performed the same tracing and analysis of the ventral portion of peripheral motor nerves as described above (Fig. 7e) and observed no significant differences in motor axon arborization measured by terminal points in *tnn* crispants compared to control larvae (*P* = 0.5) (Cas9-injected emm [95% CI] = 75.1 [67.9,83.0], *tnn* crispant emm [95% CI] = 78.5 [70.6,87.4] terminal points) (Fig. 7f and g). These findings, taken together, lead us to conclude that genetic compensation is unlikely the sole mechanism driving the differences in the phenotypes between 2 and 3 dpf in the stable knockout of *tnn.*

**Fig. 7.**
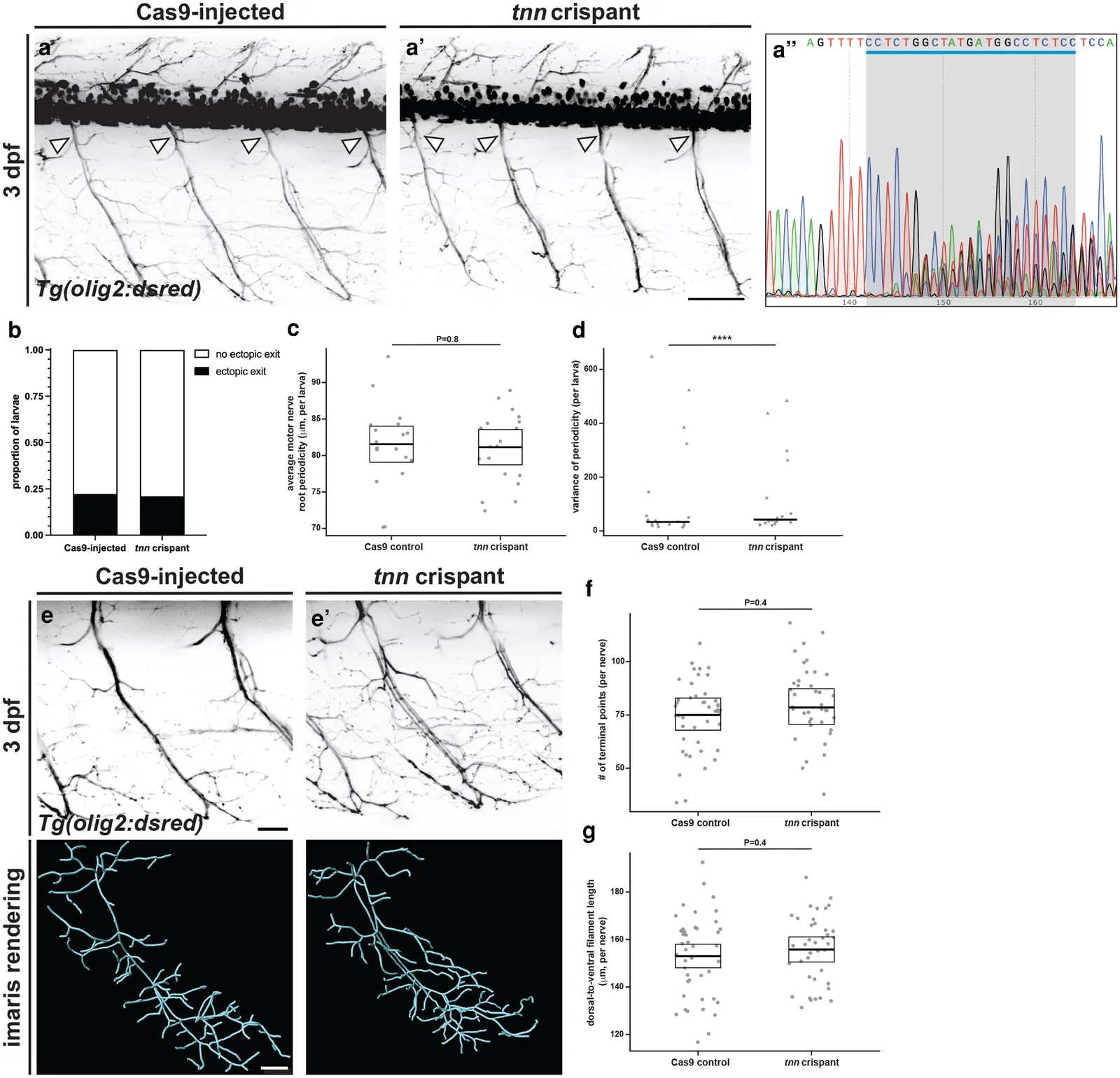
CRISPR/Cas9 mosaic mutagenesis of *tnn* does not affect peripheral spinal motor nerve exit or motor axon arborization at 3 dpf. a) Representative images from in vivo, confocal images of laterally mounted *Tg(olig2:dsred)* Cas9-injected control (a) and *tnn* crispant larvae (a′) at 3 dpf showing instances of normally placed ventral motor nerve exits (white arrowheads). a′′) Example chromatogram from *tnn* crispant larvae showing multiple superimposed sequences after the target sequence of the gRNA (blue underline, gray shaded region). b) Stacked histogram of proportion of larvae with at least 1 instance of ectopic nerve exit and larvae with no instances of ectopic exit (Cas9 control *n* = 18, *tnn* crispant *n* = 19; Fisher's exact test *P* = 0.7). c) Scatter plot of the average periodicity per larva (measured as the distance between each nerve exit and its nearest neighbor) (dot = 1 larvae; Cas9 control *n* = 18, *tnn* crispant *n* = 19). d) Scatter plot of the average variance of periodicity per larva (dot/triangle = 1 larva; dot = larva with no instances of ectopic exit; triangle = larva with at least 1 instance of ectopic exit; Cas9 control *n* = 18, *tnn* crispant *n* = 19). ****P < 0.0001. e) Representative images (top images) from in vivo, confocal imaging of laterally mounted *Tg(olig2:dsred)* Cas9 control (e) and *tnn* crispant (e′) larvae at 3 dpf enlarged to show a single nerve selected for tracing with the corresponding Imaris 3D rendering produced from tracing shown below. f) Scatter plot of the number of terminal points per nerve (dot = 1 nerve; Cas9 control *n* = 44, *tnn* crispant *n* = 39). g) Scatter plot of the distance from the most dorsal point to the most ventral point of each traced nerve (dot = 1 nerve; Cas9 control *n* = 44, *tnn* crispant *n* = 39). Summary statistics for each scatter plot represent the emm with 95% CI predicted from the described model. *P* values generated from post hoc Tukey HSD. All images oriented with anterior to the left and dorsal to the top. All images taken between somites 10 and 22. Scale bars: (a′) 50 μm; (f) 20 μm. Chromatogram generated from SnapGene.

We also sought to examine whether CRISPR/Cas9 mosaic mutagenesis could replicate the phenotypes we observed at 2 dpf in our stable *tnn* mutant larvae. We performed the same injection strategy described above to create crispants and Cas9-injected controls, raised the embryos to 2 dpf, used in vivo imaging to characterize motor nerve exit, and confirmed cutting efficiency via sequencing (Supplementary Fig. 3a). Of all nerves imaged, 5.3% were the result of ectopic exit in Cas9-injected controls compared to 6.7% in *tnn* crispants. Furthermore, 41% of control larvae displayed at least 1 ectopic nerve exit compared to 47% of *tnn* crispant larvae (Supplementary Fig. 3b; Fisher's exact test, *P* = 0.8). Although the controls and crispants show similar proportions of larvae with ectopic exit, 8 of the 9 Cas9-injected control larvae displayed only 1 ectopic exit, whereas 4 of 8 *tnn* crispant larvae displayed 2 ectopic exits, similar to what we observed at 2 dpf in our alternative gRNA *tnn* crispants. We also measured average periodicity per larvae which trended lower in *tnn* crispants compared to Cas9-injected controls but was not statistically significant (*P* = 0.4) (Cas9-injected emm [95% CI] = 71.1 μm [67.2, 75.0], *tnn* crispant emm [95% CI] = 68.4 μm [63.9, 72.8]) (Supplementary Fig. 3c). Additionally, the variance of periodicity did not differ significantly between groups (*P* = 0.8) (Cas9-injected emm [95% CI] = 59.5 [32.0, 110], *tnn* crispant emm [95% CI] = 65.5 [32.3, 133]) (Supplementary Fig. 3d). These results show similar trends as to what we observed at 2 dpf in our *tnn^uva96^* allele and our alternative gRNA crispants; however, the lack of statistically significant results leads us to conclude that mitigating genetic compensation via CRISPR/Cas9 mosaic mutagenesis does not worsen these axonal exit effect phenotypes at either 2 or 3 dpf.

## Discussion

In this study, we investigated the role of an understudied ECM protein, Tnn, in the development of spinal motor nerves. While we know from previous studies that the ECM plays important roles throughout this process, many of the proteins and their specific roles are still unknown. Here, we describe a subtle and transient role for Tnn in motor axon exit and arborization in developing zebrafish.

*Tnn* is expressed as early as 24 hpf in zebrafish embryos in cells adjacent to the neural tube and outgrowing motor axons (Weber et al. 1998). However, it was not known whether *tnn* contributed to the development of spinal motor nerves. We show that *tnn*/Tnn is expressed and localized along vertical myosepta and the border of the ventral neural tube from 1 to 3 dpf, the window in which MNs extend axons out of the spinal cord and grow toward their muscle targets. Additionally, we created a novel putative null mutant allele, *tnn^uva96^*, to interrogate the function of *tnn* during this critical window of peripheral nervous system development. With in vivo imaging and morphological analysis, we show that loss of *tnn* results in a transient trend toward increased incidence of ectopic motor axon exit from the neural tube and a transient trend toward increased arborization of motor axons at 2 dpf. However, by 3 dpf, only heterozygous mutant larvae maintain this trend toward increased incidence of ectopic nerve exits and a significantly increased variance of periodicity, leading us to conclude that *tnn* contributes to early spinal motor development, but that loss of 2 copies of *tnn* may induce a genetic compensation response.

The appearance of ectopic motor axons is described in multiple studies, often resulting from loss of diffusible guidance cues and/or their cognate receptors (Birely et al. 2005; Feldner, et al. 2007; Palaisa and Granato 2007). In these studies, ectopic exits were commonly caused by aberrant placement of primary MNs or misguided axons from primary MNs to MEP TZs (Birely et al. 2005; Feldner, et al. 2007; Palaisa and Granato 2007). In our study, we did not observe expression of *tnn/*Tnn within the neural tube nor did we see any ectopic exits until after primary MNs had already exited the spinal cord, which leads us to hypothesize that *tnn* does not impact primary MN cell body placement nor guidance of primary motor axons out of the spinal cord. The localization of Tnn adjacent to the ventral neural tube from 2 dpf on and trend toward increased the incidence of ectopic exits at this timepoint lead us to hypothesize that *tnn* loss affects permissibility of axonal exit from the spinal cord by secondary MNs. This could be a consequence of a direct mechanism that restricts axonal exit, where growing neurites contact Tnn at the boundary of the neural tube through a cell surface receptor, or an indirect mechanism, by which the loss of *tnn* could result in alterations to the ECM architecture surrounding the neural tube, leading to domains of increased axonal permissibility. All members of the tenascin family have predicted integrin binding sites within their FNIII domains supporting a possible direct mechanism (Chiquet-Ehrismann and Tucker 2011; Tucker and Degen 2019). For example, Neidhardt et al. (2003) showed that a substrate coated with a small splice variant of TNN repelled hippocampal neurites in vitro, and Degen et al. (2007) demonstrated that fibroblast cells adhere to a human TNN-coated substratum in a β1-integrin dependent manner. Recent findings of Nemoz-Billet et al. (2024), however, offer support for an indirect mechanism due to ECM rearrangement. They demonstrated that loss of *col15a1b* results in the disorganization of Tnc in the path of growing motor axons, and this ultimately leads to increased branching of motor axons at 27 hpf, a finding they also observe with the loss of *tnc* (Nemoz-Billet et al. 2024). Deciphering whether the loss of *tnn* leaves growing axons without a source of contact or changes the overall ECM architecture would provide a clearer understanding by which ectopic exits occur. While both hypotheses are possible causes of the ectopic phenotype we observe, we hypothesize that an indirect mechanism is more likely the cause of the trend toward increased branching we saw, primarily because Tnn is not localized in the pathway of growing motor axons. Therefore, it seems more likely that the loss of Tnn along vertical myosepta could cause reorganization of adjacent ECM proteins, creating an environment more permissible to branching. Uncovering which specific cell types are responsible for *tnn* expression along vertical myosepta and the ventral neural tube will aid in our understanding of which of these mechanisms drives the changes we observed.

Although we showed a similar pattern of expression and localization at 2 and 3 dpf, we observed a change in phenotypic trends between heterozygous and homozygous mutant larvae at 3 dpf. The incidence of ectopic exit trend did not persist at 3 dpf in homozygous mutant larvae but did in heterozygotes. Similarly at 3 dpf, *tnn* heterozygous larvae with *tnc* knockdown showed an even more dramatic trend toward increased incidence of ectopic exit compared to *tnn* homozygous larvae with *tnc* knockdown. Lastly, mosaic knockdown of *tnn* in wild-type embryos did not result in any differences in incidence of ectopic exit nor motor axon arborization at 3 dpf. Additionally, at both 1 and 3 dpf despite nonsignificant changes to incidence of ectopic exit and periodicity, *tnn* heterozygotes displayed significant increases in the variance of periodicity compared to wild-types and homozygotes, further suggesting that *tnn* has subtle impacts on the spacing of spinal motor axon exit.

All of this together led us to 2 major conclusions. First, although the trends we observed in the incidence of ectopic exit and motor axon arborization at 2 and 3 dpf in the stable knockout of *tnn* were not significantly different, the subtle difference at these 2 timepoints could be due to a more robust genetic compensation response in heterozygotes compared to homozygotes. Two groups have demonstrated that mutant mRNA containing a premature termination codon and nonsense-mediated decay proteins trigger the genetic compensation response and that for some genes, this effect is less robust in heterozygous mutants than in homozygous mutants (El-Brolosy et al. 2019; Ma et al. 2019). We investigated the possible compensation role of Tnc and ultimately found no evidence that Tnc compensates for the loss of *tnn.* Unfortunately, there are limited in vivo molecular interactions defined for Tnn in zebrafish, which makes the search for compensating genes/proteins challenging at this time. As more of these interactions are uncovered, we will be better equipped to investigate the difference in trends we observed across timepoints and the reason for the subtle nature of these trends. This leads into our second conclusion: our results highlight the complexity of the network of proteins that contribute to the assembly of spinal motor nerves. Transient phenotypes during this critical window of spinal motor nerve development have also been reported for Tnc and cannot be attributed to genetic compensation alone (Schweitzer et al. 2005). For example, overexpressing of either the Tnc protein or RNA encoding TNC's EGF domains resulted in truncated motor axon extension at 24 hpf but resolved by 33 hpf (Schweitzer et al. 2005). Furthermore, reduction of *tnc* expression resulted in aberrant branching not seen at 24 hpf but was apparent by 33 hpf (Schweitzer et al. 2005). Notably, these phenotypes were not investigated beyond 2 dpf. While much of our data here show trends of increased incidence of ectopic exit and motor axon arborization that lack statistical significance, we do not believe that this diminishes that Tnn functions in these processes. We believe it only adds to the already established conclusion that the ECM network that orchestrates the assembly of peripheral motor nerves is vast and complex and that Tnn is just 1 molecular mediator of many. The loss of Tnn may not be detrimental to the development of peripheral motor nerves, but its loss certainly results in changes to the process.

In conclusion, we describe a novel role for an understudied ECM protein, Tnn, in early spinal motor nerve morphogenesis. Our findings open the door to further investigation of the mechanisms by which Tnn affects this process and how it fits into the broader network of ECM proteins that influence peripheral motor nerve development. Our work further emphasizes the need to continue deciphering the connections in this complex network and how all of these proteins work in concert to achieve construction of peripheral motor nerves.

## Supplementary Material

jkag259_Supplementary_Data

## Data Availability

All data necessary for confirming the conclusion of the article are provided and presented in the text and figures within the manuscript and the Supplementary material. Fish lines, molecular tools, and detailed protocols used are available upon request. Statistical workflow documentation is provided in the Supplementary material. Supplemental material available at *G3* online.

## References

[jkag259-B1] Beattie CE et al 1997. Temporal separation in the specification of primary and secondary motoneurons in zebrafish. Dev Biol. 187:171–182. 10.1006/dbio.1997.8604. 9242415

[jkag259-B2] Bernhardt RR, Schachner M. 2000. Chondroitin sulfates affect the formation of the segmental motor nerves in zebrafish embryos. Dev Biol. 221:206–219. 10.1006/dbio.2000.9673. 10772802

[jkag259-B3] Binari LA, Lewis GM, Kucenas S. 2013. Perineurial glia require notch signaling during motor nerve development but not regeneration. J. Neurosci. 33:4241–4252. 10.1523/JNEUROSCI.4893-12.2013. PMC3668856 23467342

[jkag259-B4] Birely J et al 2005. Genetic screens for genes controlling motor nerve-muscle development and interactions. Dev Biol. 280:162–176. 10.1016/j.ydbio.2005.01.012. 15766756

[jkag259-B5] Bonanomi D, Pfaff SL. 2010. Motor axon pathfinding. Cold Spring Harb Perspect Biol. 2:. 10.1101/cshperspect.a001735. PMC2829954 20300210

[jkag259-B6] Brösamle C, Halpern ME. 2002. Characterization of myelination in the developing zebrafish. Glia. 39:47–57. 10.1002/glia.10088. 12112375

[jkag259-B7] Brose K et al 1999. Slit proteins bind Robo receptors and have an evolutionarily conserved role in repulsive axon guidance. Cell. 96:795–806. 10.1016/S0092-8674(00)80590-5. 10102268

[jkag259-B8] Bucchia M, Merwin SJ, Re DB, Kariya S. 2018. Limitations and challenges in modeling diseases involving spinal motor neuron degeneration in vitro. Front Cell Neurosci. 12:61. 10.3389/fncel.2018.00061. PMC5845677 29559895

[jkag259-B9] Buglo E et al 2020. Genetic compensation in a stable slc25a46 mutant zebrafish: a case for using F0 CRISPR mutagenesis to study phenotypes caused by inherited disease. PLoS One. 15:e0230566. 10.1371/journal.pone.0230566. PMC7092968 32208444

[jkag259-B10] Chiquet-Ehrismann R, Tucker RP. 2011. Tenascins and the importance of adhesion modulation. Cold Spring Harb Perspect Biol. 3:a004960. 10.1101/cshperspect.a004960. PMC3101840 21441591

[jkag259-B11] Clotman F, Tissir F. 2018. Guidance of motor axons: where do we stand? Curr Opin Physiol. 4:76–81. 10.1016/j.cophys.2018.06.004.

[jkag259-B12] Degen M et al 2007. Tenascin-W is a novel marker for activated tumor stroma in low-grade human breast cancer and influences cell behavior. Cancer Res. 67:9169–9179. 10.1158/0008-5472.CAN-07-0666. 17909022

[jkag259-B13] El-Brolosy MA et al 2019. Genetic compensation triggered by mutant mRNA degradation. Nature. 568:193–197. 10.1038/s41586-019-1064-z. PMC6707827 30944477

[jkag259-B14] Feldner J et al 2007. Plexina3 restricts spinal exit points and branching of trunk motor nerves in embryonic zebrafish. J. Neurosci. 27:4978–4983. 10.1523/JNEUROSCI.1132-07.2007. PMC6672091 17475806

[jkag259-B15] Fontenas L, Kucenas S. 2021. Spinal cord precursors utilize neural crest cell mechanisms to generate hybrid peripheral myelinating glia. eLife. 10:e64267. 10.7554/eLife.64267. PMC7886336 33554855

[jkag259-B16] Gagnon JA et al 2014. Efficient mutagenesis by Cas9 protein-mediated oligonucleotide insertion and large-scale assessment of single-guide RNAs. PLoS One. 9:e98186. 10.1371/journal.pone.0098186. PMC4038517 24873830

[jkag259-B17] Guillon E, Bretaud S, Ruggiero F. 2016. Slow muscle precursors lay down a collagen XV matrix fingerprint to guide motor axon navigation. J Neurosci. 36:2663–2676. 10.1523/JNEUROSCI.2847-15.2016. PMC6604867 26937007

[jkag259-B18] Hauptmann G, Gerster T. 2000. Multicolor whole-mount in situ hybridization. In: Developmental biology protocols. Humana Press. p. 139–148. 10.1385/1-59259-066-7:139. 10948532

[jkag259-B19] Hilario JD, Wang C, Beattie CE. 2010. Collagen XIXa1 is crucial for motor axon navigation at intermediate targets. Development. 137:4261–4269. 10.1242/dev.051730. PMC2990213 21098567

[jkag259-B20] Huber AB et al 2005. Distinct roles for secreted semaphorin signaling in spinal motor axon guidance. Neuron. 48:949–964. 10.1016/j.neuron.2005.12.003. 16364899

[jkag259-B21] Hwang WY et al 2013. Efficient genome editing in zebrafish using a CRISPR-Cas system. Nat Biotechnol. 31:227–229. 10.1038/nbt.2501. PMC3686313 23360964

[jkag259-B22] Jessen KR, Mirsky R. 2005. The origin and development of glial cells in peripheral nerves. Nat Rev Neurosci. 6:671–682. 10.1038/nrn1746. 16136171

[jkag259-B23] Kim M et al 2015. Motor neuron cell bodies are actively positioned by Slit/Robo repulsion and netrin/DCC attraction. Dev Biol. 399:68–79. 10.1016/j.ydbio.2014.12.014. PMC4339514 25530182

[jkag259-B24] Kim M, Fontelonga TM, Lee CH, Barnum SJ, Mastick GS. 2017. Motor axons are guided to exit points in the spinal cord by slit and netrin signals. Dev Biol. 432:178–191. 10.1016/j.ydbio.2017.09.038. PMC5694371 28986144

[jkag259-B25] Kimmel CB, Ballard WW, Kimmel SR, Ullmann B, Schilling TF. 1995. Stages of embryonic development of the zebrafish. Dev Dyn. 203:253–310. 10.1002/aja.1002030302. 8589427

[jkag259-B26] Kok FO et al 2015. Reverse genetic screening reveals poor correlation between morpholino-induced and mutant phenotypes in zebrafish. Dev Cell. 32:97–108. 10.1016/j.devcel.2014.11.018. PMC4487878 25533206

[jkag259-B27] Kucenas S et al 2008b. CNS-derived glia ensheath peripheral nerves and mediate motor root development. Nat Neurosci. 11:143–151. 10.1038/nn2025. PMC2657597 18176560

[jkag259-B28] Kucenas S, Snell H, Appel B. 2008a. Nkx2.2a promotes specification and differentiation of a myelinating subset of oligodendrocyte lineage cells in zebrafish. Neuron Glia Biol. 4:71–81. 10.1017/S1740925X09990123. PMC2821284 19737431

[jkag259-B29] Lieberam I, Agalliu D, Nagasawa T, Ericson J, Jessell TM. 2005. A Cxcl12-Cxcr4 chemokine signaling pathway defines the initial trajectory of mammalian motor axons. Neuron. 47:667–679. 10.1016/j.neuron.2005.08.011. 16129397

[jkag259-B30] Lyons DA et al 2005. Erbb3 and erbb2 are essential for Schwann cell migration and myelination in zebrafish. Curr Biol. 15:513–524. 10.1016/j.cub.2005.02.030. 15797019

[jkag259-B31] Ma Z et al 2019. PTC-bearing mRNA elicits a genetic compensation response via Upf3a and COMPASS components. Nature. 568:259–263. 10.1038/s41586-019-1057-y. 30944473

[jkag259-B32] Moreno RL, Williams K, Jones KL, Ribera AB. 2018. Investigation of Islet2a function in zebrafish embryos: mutants and morphants differ in morphologic phenotypes and gene expression. PLoS One. 13:e0199233. 10.1371/journal.pone.0199233. PMC6013100 29927984

[jkag259-B33] Moreno-Mateos MA et al 2015. CRISPRscan: designing highly efficient sgRNAs for CRISPR-Cas9 targeting in vivo. Nat Methods. 12:982–988. 10.1038/nmeth.3543. PMC4589495 26322839

[jkag259-B34] Morris AD, Lewis GM, Kucenas S. 2017. Perineurial glial plasticity and the role of TGF-β in the development of the blood–nerve barrier. J Neurosci. 37:4790–4807. 10.1523/JNEUROSCI.2875-16.2017. PMC5426569 28389474

[jkag259-B35] Myers PZ . 1985. Spinal motoneurons of the larval zebrafish. J Comp Neurol. 236:555–561. 10.1002/cne.902360411. 4056102

[jkag259-B36] Myers PZ, Eisen JS, Westerfield M. 1986. Development and axonal outgrowth of identified motoneurons in the zebrafish. J Neurosci. 6:2278–2289. 10.1523/JNEUROSCI.06-08-02278.1986. PMC6568750 3746410

[jkag259-B37] Neidhardt J, Fehr S, Kutsche M, Löhler J, Schachner M. 2003. Tenascin-N: characterization of a novel member of the tenascin family that mediates neurite repulsion from hippocampal explants. Mol Cell Neurosci. 23:193–209. 10.1016/S1044-7431(03)00012-5. 12812753

[jkag259-B38] Nemoz-Billet L et al 2024. Dual topologies of myotomal collagen XV and Tenascin C act in concert to guide and shape developing motor axons. Proc Natl Acad Sci U S A. 121:e2314588121. 10.1073/pnas.2314588121. PMC10990108 38502691

[jkag259-B39] Palaisa KA, Granato M. 2007. Analysis of zebrafish sidetracked mutants reveals a novel role for Plexin A3 in intraspinal motor axon guidance. Development. 134:3251–3257. 10.1242/dev.007112. 17699603

[jkag259-B40] Patel T et al 2022. Transcriptional dynamics of murine motor neuron maturation in vivo and in vitro. Nat Commun. 13:5427. 10.1038/s41467-022-33022-4. PMC9477853 36109497

[jkag259-B41] Pike SH, Melancon EF, Eisen JS. 1992. Pathfinding by zebrafish motoneurons in the absence of normal pioneer axons. Development. 114:825–831. 10.1242/dev.114.4.825. 1618146

[jkag259-B42] Raible DW et al 1992. Segregation and early dispersal of neural crest cells in the embryonic zebrafish. Dev Dyn. 195:29–42. 10.1002/aja.1001950104. 1292751

[jkag259-B43] Raible DW, Eisen JS. 1994. Restriction of neural crest cell fate in the trunk of the embryonic zebrafish. Development. 120:495–503. 10.1242/dev.120.3.495. 8162850

[jkag259-B44] Rossi A et al 2015. Genetic compensation induced by deleterious mutations but not gene knockdowns. Nature. 524:230–233. 10.1038/nature14580. 26168398

[jkag259-B45] Rothberg JM, Jacobs JR, Goodman CS, Artavanis-Tsakonas S. 1990. Slit: an extracellular protein necessary for development of midline glia and commissural axon pathways contains both EGF and LRR domains. Genes Dev. 4:2169–2187. 10.1101/gad.4.12a.2169. 2176636

[jkag259-B46] Schilling TF, Kimmel CB. 1994. Segment and cell type lineage restrictions during pharyngeal arch development in the zebrafish embryo. Development. 120:483–494. 10.1242/dev.120.3.483. 8162849

[jkag259-B47] Schneider VA, Granato M. 2006. The myotomal diwanka (lh3) glycosyltransferase and type XVIII collagen are critical for motor growth cone migration. Neuron. 50:683–695. 10.1016/j.neuron.2006.04.024. 16731508

[jkag259-B48] Schweitzer J et al 2005. Tenascin-C is involved in motor axon outgrowth in the trunk of developing zebrafish. Dev Dyn. 234:550–566. 10.1002/dvdy.20525. 16110513

[jkag259-B49] She J et al 2019. Genetic compensation by *epob* in pronephros development in *epoa* mutant zebrafish. Cell Cycle. 18:2683–2696. 10.1080/15384101.2019.1656019. PMC6773226 31451030

[jkag259-B50] Shin J, Park H-C, Topczewska JM, Mawdsley DJ, Appel B. 2003. Neural cell fate analysis in zebrafish using olig2 BAC transgenics. Methods Cell Sci. 25:7–14. 10.1023/B:MICS.0000006847.09037.3a. 14739582

[jkag259-B51] Smith CJ, Morris AD, Welsh TG, Kucenas S, Barres BA. 2014. Contact-mediated inhibition between oligodendrocyte progenitor cells and motor exit point glia establishes the spinal cord transition zone. PLoS Biol. 12:e1001961. 10.1371/journal.pbio.1001961. PMC4181976 25268888

[jkag259-B52] Takahashi H . 1977. Juvenile hermaphroditism in the zebrafish, Brachydanio rerio. Bulletin of Fisheries Sciences, Hokkaido University. 28:57–65.

[jkag259-B53] Truett GE et al 2000. Preparation of PCR-quality mouse genomic DNA with hot sodium hydroxide and tris (HotSHOT). BioTechniques. 29:52–54. 10.2144/00291bm09. 10907076

[jkag259-B54] Tucker RP, Degen M. 2019. The expression and possible functions of tenascin-W during development and disease. Front Cell Dev Biol. 7:53. 10.3389/fcell.2019.00053. PMC6473177 31032255

[jkag259-B55] Weber P, Montag D, Schachner M, Bernhardt RR. 1998. Zebrafish tenascin-W, a new member of the tenascin family. J Neurobiol. 35:1–16. 10.1002/(SICI)1097-4695(199804)35:1<1::AID-NEU1>3.0.CO;2-9. 9552162

[jkag259-B56] Westerfield M, McMurray JV, Eisen JS. 1986. Identified motoneurons and their innervation of axial muscles in the zebrafish. J Neurosci. 6:2267–2277. 10.1523/JNEUROSCI.06-08-02267.1986. PMC6568761 3746409

[jkag259-B57] Wiltbank AT, Steinson ER, Criswell SJ, Piller M, Kucenas S. 2022. Cd59 and inflammation regulate Schwann cell development. Elife. 11:e76640. 10.7554/eLife.76640. PMC9232220 35748863

