# Supplementary material for: Tenascin-N contributes to spinal motor nerve morphogenesis during development
Source: G3 (Bethesda). 2026 Sep 21;16(11):jkag259. doi: 10.1093/g3journal/jkag259 (PMC13732476; doi:10.1093/g3journal/jkag259)
Supplement: Supplementary file 1 [file jkag259_supplementary_data.zip › Supplemental_Figure_and_Video_Legends_G3-2026-407227.docx]

**Supplementary Figure Legends**

**Supplementary Figure 1.** (**a**) Schematic of zebrafish *tnn* gene with the coding sequence (grey; 2799 bp) and non-coding sequence represented in white. Dashed lines above coding exon 13 in schematic indicate the gRNA sequence of the alternative gRNA and target region of the coding sequence. (**b**) Representative images from *in vivo,* confocal images of laterally mounted *Tg(olig2:dsred)* Cas9-injected control (**b**) and *tnn* crispant larvae (**b’**) at 2 dpf showing instances of normally placed ventral motor nerve exits (white arrowheads) and ectopic motor nerve exits (black arrowheads). (**b’’**) Example chromatogram from a *tnn* crispant larvae showing multiple superimposed sequences after the target sequence of the gRNA (blue underline, grey shaded region). (**c**) Stacked histogram of proportion of larvae with at least one instance of ectopic nerve exit and larvae with no instances of ectopic exit (Cas9 control n=29, *tnn* crispant n=20; Fisher’s exact test P=0.7). (**c**) Scatter plot of average periodicity per larva (measured as distance between each nerve exit and its nearest neighbor) (dot = 1 larvae; Cas9 control n=29, *tnn* crispant n=20). ** (P<0.01) (**d**) Scatter plot of average variance of periodicity per larva (dot/triangle = 1 larva; dot = larva with no instances of ectopic exit; triangle = larva with at least one instance of ectopic exit; Cas9 control n=29, *tnn* crispant n=20). **** (P<0.0001). Summary statistics for each scatter plot represent the emm with 95% CI predicted from the described model. P-values generated from post-hoc Tukey HSD. Images oriented with anterior to the left, dorsal to the top. Images taken between somites 10 and 22. Scale bar: (**b’**): 50 μm. Chromatogram generated from SnapGene.

**Supplementary Figure 2.** (**a**) Representative images from *in vivo,* confocal images of laterally mounted *Tg(olig2:dsred)* *tnn* heterozygous/*tnc* crispant (**a**) and *tnn* homozygous/*tnc* crispant larvae (**a’**) larvae at 2 dpf showing instances of normally placed ventral motor nerve exits (white arrowheads) and ectopic motor nerve exits (black arrowheads). (**a’’**) Example chromatogram from a *tnc* crispant larvae showing multiple overlapping peaks after the target sequence of the gRNA (blue underline, grey shaded region) representing multiple different alleles generated from gRNA/Cas9 activity. (**b**) Stacked histogram of proportion of larvae with at least one instance of ectopic nerve exit and larvae with no instances of ectopic exit (*tnn* heterozygous/*tnc* crispant n=13, *tnn* homozygous/*tnc* crispant n=8; Fisher’s exact test P=0.3). (**c**) Scatter plot of average periodicity per larva (measured as distance between each nerve exit and its nearest neighbor) (dot = 1 larva; *tnn* heterozygous/*tnc* crispant n=13, *tnn* homozygous, *tnc* crispant n=8). (**d**) Scatter plot of average variance of periodicity per larva (dot/triangle = 1 larva; dot = larva with no instances of ectopic exit; triangle = larva with at least one instance of ectopic exit; *tnn* heterozygous/*tnc* crispant n=13, *tnn* homozygous/*tnc* crispant n=8). Summary statistics for each scatter plot represent the emm with 95% CI predicted from the described model. P-values generated from post-hoc Tukey HSD. Images oriented with anterior to the left, dorsal to the top. Images taken between somites 10 and 22. Scale bar: (**c’**): 50 μm. Chromatogram generated from SnapGene.

**Supplementary Figure 3.** (**a**) Representative images from *in vivo,* confocal images of laterally mounted *Tg(olig2:dsred)* Cas9-injected control (**a**) and *tnn* crispant larvae (**a’**) at 2 dpf showing instances of normally placed ventral motor nerve exits (white arrowheads) and ectopic motor nerve exits (black arrowheads). (**a’’**) Example chromatogram from a *tnn* crispant larvae showing multiple superimposed sequences after the target sequence of the gRNA (blue underline, grey shaded region). (**b**) Stacked histogram of proportion of larvae with at least one instance of ectopic nerve exit and larvae with no instances of ectopic exit (Cas9 control n=22, *tnn* crispant n=17; Fisher’s exact test P=0.78). (**b**) Scatter plot of average periodicity per larva (measured as distance between each nerve exit and its nearest neighbor) (dot = 1 larvae; Cas9 control n=29, *tnn* crispant n=20). (**d**) Scatter plot of average variance of periodicity per larva (dot/triangle = 1 larva; dot = larva with no instances of ectopic exit; triangle = larva with at least one instance of ectopic exit; Cas9 control n=29, *tnn* crispant n=20). Summary statistics for each scatter plot represent the emm with 95% CI predicted from the described model. P-values generated from post-hoc Tukey HSD. Images oriented with anterior to the left, dorsal to the top. Images taken between somites 10 and 22. Scale bar: (**b’**): 50 μm. Chromatogram generated from SnapGene.

**Supplementary Video 1. Appearance of an ectopic motor axon exit in wild-type larvae.** A spinal motor nerve exits at an ectopic location (magenta arrowhead) between 1 and 2 dpf in a *Tg(olig2:dsred),* wild-type larvae. Images captured every 20 minutes over the course of 16 hours beginning between 24-28 hpf. Anterior to the left, dorsal to the top. Scale bar: 50 μm.

**Supplementary Video 2. Appearance of an ectopic motor axon exit in *tnn^uva96^* heterozygous mutant larvae.** A spinal motor nerve exits at an ectopic location (magenta arrowhead) between 1 and 2 dpf in a *Tg(olig2:dsred),* *tnn^uva96^* heterozygous mutant larvae. Images captured every 20 minutes over the course of 16 hours beginning between 24-28 hpf. Anterior to the left, dorsal to the top. Scale bar: 50 μm.

**Supplementary Video 2. Appearance of an ectopic motor axon exit in *tnn^uva96^* homozygous mutant larvae.** A spinal motor nerve exits at an ectopic location (magenta arrowhead) between 1 and 2 dpf in a *Tg(olig2:dsred), tnn^uva96^* homozygous mutant larvae. Images captured every 20 minutes over the course of 16 hours beginning between 24-28 hpf. Anterior to the left, dorsal to the top. Scale bar: 50 μm.
