# Supplementary material for: Tenascin-N contributes to spinal motor nerve morphogenesis during development
Source: G3 (Bethesda). 2026 Sep 21;16(11):jkag259. doi: 10.1093/g3journal/jkag259 (PMC13732476; doi:10.1093/g3journal/jkag259)
Supplement: Supplementary file 1 [file jkag259_supplementary_data.zip › Supplemental_Material_G3-2026-407227.docx]

Analyses of Nerve Development in Zebrafish

Marieke Jones, PhD

Table of contents

[Goals	1](#_Toc238307292)

[Set up	3](#_Toc238307293)

[TNN at 2dpf	3](#_Toc238307294)

[TNN at 3dpf	18](#_Toc238307295)

[TNC crispant+TNN at 3dpf	36](#_Toc238307296)

[TNN crispant at 3dpf	45](#_Toc238307297)

[TNN at 1dpf	60](#_Toc238307298)

[Why does TNN at 2dpf have high SE in the variance analysis?	69](#_Toc238307299)

[ADDED ANALYSES BASED ON REVIEWER COMMENTS	74](#_Toc238307300)

[TNN gRNA1 (alternative gRNA) at 2dpf	74](#_Toc238307301)

[TNN gRNA2 (tnn^uva96^ gRNA) at 2dpf	85](#_Toc238307302)

[TNC crispant + TNN at 2dpf	96](#_Toc238307303)

# Goals

This script performs analyses to support the PhD studies of Charles Marcucci in the lab of Dr. Sarah Kucenas.

Specifically goals of this script are:

**TNN at 2dpf:**

1. At 2 dpf, Analyze period by genotype (wt, het, mut)
2. At 2 dpf, Analyze variance of period by genotype (wt, het, mut)
3. At 2 dpf, Analyze number of terminal points by genotype (wt, het, mut) taking into account total filament length
4. At 2 dpf, analyze total filament length by genotype (wt, het, mut)

**TNN at 3dpf:**

1. At 3 dpf, Analyze period by genotype (wt, het, mut)
2. At 3 dpf, Analyze variance of period by genotype (wt, het, mut)
3. At 3 dpf, Analyze number of terminal points by genotype (wt, het, mut) taking into account total filament length
4. At 3 dpf, analyze total filament length by genotype (wt, het, mut)

**TNC crispant+TNN at 3dpf:**

1. At 3dpf, Analyze period by genotype (crisp/het, crisp/mut)
2. At 3dpf, Analyze variance of period by genotype (crisp/het, crisp/mut)

**TNN crispant at 3dpf:**

1. At 3 dpf, analyze period by genotype (crispant and control)
2. At 3 dpf, analyze variance of period by genotype (crispant and control)
3. At 3 dpf, analyze number of terminal points by genotype (crispant and control) taking into account total filament length
4. At 3 dpf, analyze total filament length by genotype (crispant and control)

**TNN at 1dpf:**

1. At 1 dpf, Analyze period by genotype (wt, het, mut)
2. At 1 dpf, Analyze variance of period by genotype (wt, het, mut)

### Based on Reviewer comments, the team added the following

**TNN gRNA1 (alternative gRNA) at 2dpf**

1. At 2 dpf, Analyze period by genotype (cas9, crispant)
2. At 2 dpf, Analyze variance of period by genotype (cas9, crispant)

**TNN gRNA2 (tnn^uva96^ gRNA) at 2dpf**

1. At 2 dpf, Analyze period by genotype (cas9, crispant)
2. At 2 dpf, Analyze variance of period by genotype (cas9, crispant)

**TNC crispant + TNN at 2dpf**

1. At 2 dpf, Analyze period by genotype (het_crisp, mut_crisp)
2. At 2 dpf, Analyze variance of period by genotype (het_crisp, mut_crisp)

# Set up

Load the packages I will need for the rest of the script here

library(lme4)
library(lmerTest)
library(janitor)
library(tidyverse)

# TNN at 2dpf

Load the TNN 2dpf data file with ectopic column sent by email 9/22

tnn2dpf <- read_csv("2dpf_x_period_ectopic.csv")
tnn2dpf <- clean_names(tnn2dpf)

Drop empty columns

```
tnn2dpf <- tnn2dpf |>
  select(date:ectopic)
```

Create unique fish id and image id

```
tnn2dpf <- tnn2dpf |>
  mutate(fish_id = paste(fish_number, date, genotype, sep = "_"),
         image_id = paste(fish_id, image, sep = "_image"))
```

Put genotype in order: WT, HET, MUT

```
tnn2dpf <- tnn2dpf |>
  mutate(genotype = factor(genotype, levels = c("wt", "het", "mut")))
```

## 1. Analyze period

Period is the distance between nerve exits, so if the mutation is disrupting nerve placement, there may be a difference in average period.

Include random effect of image and of larva

```
tnn2dpf |>
  ggplot(aes(period)) +
  geom_density()
```

```
tnn2dpf |>
  ggplot(aes(period, linetype = genotype)) +
  geom_density()
```

Ugly but try some LMMs anyway. I’ll try one with a random effect of both image and fish and a second with just the random effect of fish to see if including the image is important.

```
permod1 <- lmer(period ~ genotype + (1|image_id) + (1|fish_id),
                   data = tnn2dpf)

permod2 <- lmer(period ~ genotype + (1|fish_id),
                   data = tnn2dpf)

anova(permod1, permod2)
Data: tnn2dpf
Models:
permod2: period ~ genotype + (1 | fish_id)
permod1: period ~ genotype + (1 | image_id) + (1 | fish_id)
        npar    AIC    BIC  logLik -2*log(L)  Chisq Df Pr(>Chisq)
permod2    5 4882.4 4904.2 -2436.2    4872.4
permod1    6 4856.8 4883.0 -2422.4    4844.8 27.596  1  1.495e-07 ***
---
Signif. codes:  0 '***' 0.001 '**' 0.01 '*' 0.05 '.' 0.1 ' ' 1
```

The better model includes the random effect of image and fish. Take a look at that model

```
anova(permod1)
Type III Analysis of Variance Table with Satterthwaite's method
         Sum Sq Mean Sq NumDF  DenDF F value Pr(>F)
genotype 363.13  181.56     2 67.582  0.9904 0.3768
summary(permod1)
Linear mixed model fit by REML. t-tests use Satterthwaite's method [
lmerModLmerTest]
Formula: period ~ genotype + (1 | image_id) + (1 | fish_id)
   Data: tnn2dpf

REML criterion at convergence: 4837

Scaled residuals:
    Min      1Q  Median      3Q     Max
-4.2016 -0.1540  0.1990  0.4972  1.5614

Random effects:
 Groups   Name        Variance Std.Dev.
 image_id (Intercept)  53.384   7.306
 fish_id  (Intercept)   2.714   1.648
 Residual             183.325  13.540
Number of obs: 587, groups:  image_id, 178; fish_id, 69

Fixed effects:
            Estimate Std. Error     df t value Pr(>|t|)
(Intercept)   76.232      1.742 75.913  43.760   <2e-16 ***
genotypehet   -2.881      2.070 72.236  -1.392    0.168
genotypemut   -1.657      2.375 69.047  -0.698    0.488
---
Signif. codes:  0 '***' 0.001 '**' 0.01 '*' 0.05 '.' 0.1 ' ' 1

Correlation of Fixed Effects:
            (Intr) gntyph
genotypehet -0.842
genotypemut -0.733  0.617
```

There is no significant difference by genotype

Retrieve the emmeans

```
emmeans::emmeans(permod1, specs = "genotype")
 genotype emmean   SE   df lower.CL upper.CL
 wt         76.2 1.74 73.7     72.8     79.7
 het        73.4 1.12 62.5     71.1     75.6
 mut        74.6 1.62 60.4     71.3     77.8

Degrees-of-freedom method: kenward-roger
Confidence level used: 0.95
pairs(emmeans::emmeans(permod1, specs = "genotype"))
 contrast  estimate   SE   df t.ratio p.value
 wt - het      2.88 2.07 70.2   1.390  0.3514
 wt - mut      1.66 2.38 67.2   0.697  0.7662
 het - mut    -1.22 1.97 61.1  -0.623  0.8082

Degrees-of-freedom method: kenward-roger
P value adjustment: tukey method for comparing a family of 3 estimates
```

### Plot

Show the average for each fish as the raw data and the emmeans with 95% CI as the summary stat

First generate the average period for each fish

```
fish <- tnn2dpf |>
  group_by(genotype, fish_id) |>
  summarize(avg_per = mean(period, na.rm = T),
            .groups = "drop")
```

Save the emmeans as a dataset

```
permod1_emm <- emmeans::emmeans(permod1, specs = "genotype") |>
  as_tibble()
```

Now plot

```
plot_tnn_2dpf_period <- fish |>
  ggplot(aes(genotype, avg_per)) +
  geom_jitter(width = .2,
              color = "gray60") +
  geom_crossbar(data = permod1_emm,
                aes(genotype, y = emmean,
                    ymin = lower.CL,
                    ymax = upper.CL),
                width = .4) +
  labs(y = "average periodicty (per larvae)") +
  theme_classic()

plot_tnn_2dpf_period
```

```
ggsave(plot_tnn_2dpf_period,
       filename = "plots/tnn_2dpf_period.tiff",
       height = 4, width = 5)
```

## 2. Analyze variance of period

Because we see a difference in the variance of the period, we can analyze that directly.

First create the variance of the period for each image

```
var_per <- tnn2dpf |>
  group_by(genotype, fish_id, image_id) |>
  summarize(var_per = var(period, na.rm = T))
```

See these variances

```
var_per |>
  ggplot(aes(var_per, linetype = genotype)) +
  geom_density()
```

```
var_per |>
  ggplot(aes(genotype, var_per)) +
  geom_jitter(width = .2)
```

How many images and fish per genotype?

```
# number of images per genotype
var_per |>
  ungroup() |>
  count(genotype)
# A tibble: 3 × 2
  genotype     n
  <fct>    <int>
1 wt          39
2 het         93
3 mut         46
# number of fish per genotype
var_per |>
  ungroup() |>
  group_by(genotype) |>
  summarize(n_fish = n_distinct(fish_id))
# A tibble: 3 × 2
  genotype n_fish
  <fct>     <int>
1 wt           16
2 het          36
3 mut          17
```

Then model using a gamma regression

```
varmod1 <- glmer(var_per ~ genotype + (1|fish_id),
              data = var_per,
              family = Gamma(link = "log"))
```

See that model output

```
summary(varmod1)
Generalized linear mixed model fit by maximum likelihood (Laplace
  Approximation) [glmerMod]
 Family: Gamma  ( log )
Formula: var_per ~ genotype + (1 | fish_id)
   Data: var_per

      AIC       BIC    logLik -2*log(L)  df.resid
   1963.1    1979.0    -976.6    1953.1       173

Scaled residuals:
    Min      1Q  Median      3Q     Max
-0.8201 -0.6992 -0.3084  0.4640  2.5372

Random effects:
 Groups  Name        Variance Std.Dev.
 fish_id (Intercept) 1.654    1.286
Number of obs: 178, groups:  fish_id, 69

Fixed effects:
            Estimate Std. Error t value Pr(>|z|)
(Intercept)   3.5988     0.3799   9.472   <2e-16 ***
genotypehet   0.4832     0.4536   1.065    0.287
genotypemut   0.7299     0.5248   1.391    0.164
---
Signif. codes:  0 '***' 0.001 '**' 0.01 '*' 0.05 '.' 0.1 ' ' 1

Correlation of Fixed Effects:
            (Intr) gntyph
genotypehet -0.832
genotypemut -0.718  0.602
```

See the predicted variance for each genotype

```
emmeans::emmeans(varmod1, specs = "genotype", type = "response")
 genotype response   SE  df asymp.LCL asymp.UCL
 wt           36.6 13.9 Inf      17.4      77.0
 het          59.3 14.9 Inf      36.2      97.1
 mut          75.8 27.7 Inf      37.1     155.2

Confidence level used: 0.95
Intervals are back-transformed from the log scale
pairs(emmeans::emmeans(varmod1, specs = "genotype"))
 contrast  estimate    SE  df z.ratio p.value
 wt - het    -0.483 0.454 Inf  -1.065  0.5357
 wt - mut    -0.730 0.525 Inf  -1.391  0.3457
 het - mut   -0.247 0.441 Inf  -0.559  0.8417

Results are given on the log (not the response) scale.
P value adjustment: tukey method for comparing a family of 3 estimates
```

There does seem to be a difference, but the variability is so large that the statistical significance is erased

### Plot

First generate the variance of the period for each fish

```
fishvar <- tnn2dpf |>
  group_by(fish_id) |>
  fill(ectopic, .direction = "updown") |>
  ungroup() |>
  group_by(genotype, fish_id, ectopic) |>
  summarize(var_per = var(period, na.rm = T),
            .groups = "drop") |>
  mutate(ectopic = if_else(is.na(ectopic), 0, ectopic),
         ectopic = factor(ectopic))
```

Save the emmeans as a dataset

```
varmod1_emm <- emmeans::emmeans(varmod1, specs = "genotype", type = "response") |>
  as_tibble()
```

Now plot

```
plot_tnn_2dpf_variance <- fishvar |>
  ggplot(aes(genotype, var_per)) +
  geom_jitter(width = .2,
              color = "gray60",
              aes(shape = ectopic)) +
  geom_crossbar(data = varmod1_emm,
                aes(genotype, y = response,
                    ymin = asymp.LCL,
                    ymax = asymp.UCL),
                width = .4) +
  labs(y = "variance of the periodicty (per larvae)") +
  theme_classic() +
  theme(legend.position = "none")

plot_tnn_2dpf_variance
```

```
ggsave(plot_tnn_2dpf_variance,
       filename = "plots/tnn_2dpf_variance.tiff",
       height = 4, width = 5)
```

## 3. Analyze number of terminal points

Number of terminal points is counted for each image, so we will need a random effect for each fish. We should account for the fact that some nerves are longer so they would have more terminal points, so add total filament length (length of nerve) as an offset

First load these data

tnntrace2dpf <- read_csv("csvs_MJ/2dpf_tnn_tracing.csv")

Make a unique fish ID and an image ID

```
tnntrace2dpf <- tnntrace2dpf |>
  mutate(fish_id = paste(fish, date, genotype, sep = "_"),
         image_id = paste(fish_id, image, sep = "_image"))
```

Put genotype in order: WT, HET, MUT

```
tnntrace2dpf <- tnntrace2dpf |>
  mutate(genotype = factor(genotype, levels = c("wt", "het", "mut")))
```

Look at raw number of terminal points by genotype. This ignores the nesting

```
tnntrace2dpf |>
  ggplot(aes(genotype, terminal_pts)) +
  geom_jitter(width = .2)
```

Model using Poisson taking into account the main_distance of each nerve

```
termmod1 <- glmer(terminal_pts ~ genotype + (1|fish_id),
                   family = poisson,
                   offset = log(main_distance),
                   data = tnntrace2dpf)

summary(termmod1)
Generalized linear mixed model fit by maximum likelihood (Laplace
  Approximation) [glmerMod]
 Family: poisson  ( log )
Formula: terminal_pts ~ genotype + (1 | fish_id)
   Data: tnntrace2dpf
 Offset: log(main_distance)

      AIC       BIC    logLik -2*log(L)  df.resid
   1080.9    1093.0    -536.4    1072.9       148

Scaled residuals:
     Min       1Q   Median       3Q      Max
-1.93224 -0.53624  0.08164  0.49976  2.03931

Random effects:
 Groups  Name        Variance Std.Dev.
 fish_id (Intercept) 0.01478  0.1216
Number of obs: 152, groups:  fish_id, 53

Fixed effects:
            Estimate Std. Error z value Pr(>|z|)
(Intercept) -0.99987    0.04083 -24.489   <2e-16 ***
genotypehet  0.09731    0.05056   1.925   0.0543 .
genotypemut  0.07724    0.05529   1.397   0.1624
---
Signif. codes:  0 '***' 0.001 '**' 0.01 '*' 0.05 '.' 0.1 ' ' 1

Correlation of Fixed Effects:
            (Intr) gntyph
genotypehet -0.807
genotypemut -0.738  0.596
```

See how many terminal points each genotype is predicted by this model to have

```
emmeans::emmeans(termmod1, specs = "genotype", type = "response")
 genotype rate   SE  df asymp.LCL asymp.UCL
 wt       51.5 2.10 Inf      47.5      55.8
 het      56.7 1.69 Inf      53.5      60.2
 mut      55.6 2.07 Inf      51.7      59.8

Confidence level used: 0.95
Intervals are back-transformed from the log scale
pairs(emmeans::emmeans(termmod1, specs = "genotype"))
 contrast  estimate     SE  df z.ratio p.value
 wt - het   -0.0973 0.0506 Inf  -1.925  0.1317
 wt - mut   -0.0772 0.0553 Inf  -1.397  0.3424
 het - mut   0.0201 0.0478 Inf   0.420  0.9073

Results are given on the log (not the response) scale.
P value adjustment: tukey method for comparing a family of 3 estimates
```

For the average main_distance, WT is expected to have 51 terminal points, while het has 57 and mut has 56

### Plot

This plot is on the image level

Save the emmeans as a dataset

```
termmod1_emm <- emmeans::emmeans(termmod1, specs = "genotype",
                                 type = "response") |>
  as_tibble()
```

Now plot

```
plot_tnn_2dpf_termpts <- tnntrace2dpf |>
  ggplot(aes(genotype, terminal_pts)) +
  geom_jitter(width = .2,
              color = "gray60") +
  geom_crossbar(data = termmod1_emm,
                aes(genotype, y = rate,
                    ymin = asymp.LCL,
                    ymax = asymp.UCL),
                width = .4) +
  labs(y = "number of terminal points (per image)") +
  theme_classic()

plot_tnn_2dpf_termpts
```

```
ggsave(plot_tnn_2dpf_termpts,
       filename = "plots/tnn_2dpf_termpts.tiff",
       height = 4, width = 5)
```

## 4. Analyze total filament length

Random effect for each fish

See a plot

```
tnntrace2dpf |>
  ggplot(aes(genotype, main_distance)) +
  geom_jitter(width = .2)
```

Now model

```
distmod1 <- lmer(main_distance ~ genotype + (1|fish_id),
                   data = tnntrace2dpf)
summary(distmod1)
Linear mixed model fit by REML. t-tests use Satterthwaite's method [
lmerModLmerTest]
Formula: main_distance ~ genotype + (1 | fish_id)
   Data: tnntrace2dpf

REML criterion at convergence: 1217.6

Scaled residuals:
     Min       1Q   Median       3Q      Max
-2.33741 -0.67222 -0.06797  0.63211  2.31918

Random effects:
 Groups   Name        Variance Std.Dev.
 fish_id  (Intercept)  27.16    5.211
 Residual             169.13   13.005
Number of obs: 152, groups:  fish_id, 53

Fixed effects:
            Estimate Std. Error      df t value Pr(>|t|)
(Intercept)  135.794      2.494  32.158  54.439   <2e-16 ***
genotypehet    7.394      3.177  37.987   2.327   0.0254 *
genotypemut    6.092      3.428  33.890   1.777   0.0846 .
---
Signif. codes:  0 '***' 0.001 '**' 0.01 '*' 0.05 '.' 0.1 ' ' 1

Correlation of Fixed Effects:
            (Intr) gntyph
genotypehet -0.785
genotypemut -0.728  0.571
```

See the predicted means

```
emmeans::emmeans(distmod1, specs = "genotype")
 genotype emmean   SE   df lower.CL upper.CL
 wt          136 2.50 37.7      131      141
 het         143 1.97 57.8      139      147
 mut         142 2.36 41.9      137      147

Degrees-of-freedom method: kenward-roger
Confidence level used: 0.95
pairs(emmeans::emmeans(distmod1, specs = "genotype"))
 contrast  estimate   SE   df t.ratio p.value
 wt - het     -7.39 3.18 44.1  -2.322  0.0631
 wt - mut     -6.09 3.44 39.6  -1.772  0.1920
 het - mut     1.30 3.07 47.7   0.424  0.9059

Degrees-of-freedom method: kenward-roger
P value adjustment: tukey method for comparing a family of 3 estimates
```

WT is expected to have a shorter total filament, but the difference is not quite statistically significant

### Plot

This plot is on the image level

Save the emmeans as a dataset

```
distmod1emm <- emmeans::emmeans(distmod1, specs = "genotype") |>
  as_tibble()
```

Now plot

```
plot_tnn_2dpf_length <- tnntrace2dpf |>
  ggplot(aes(genotype, main_distance)) +
  geom_jitter(width = .2,
              color = "gray60") +
  geom_crossbar(data = distmod1emm,
                aes(genotype, y = emmean,
                    ymin = lower.CL,
                    ymax = upper.CL),
                width = .4) +
  labs(y = "dorsal to ventral filament length (per image)") +
  theme_classic()

plot_tnn_2dpf_length
```

```
ggsave(plot_tnn_2dpf_length,
       filename = "plots/tnn_2dpf_length.tiff",
       height = 4, width = 5)
```

# TNN at 3dpf

Load the TNN 3dpf data file.

Note that the original file was missing image number for all fish on 7052024 so I added those in and created the file 3dpf_tnn_periodicity_edited.csv

tnn3dpf <- read_csv("csvs_MJ/3dpf_tnn_periodicity_edited.csv")

Create unique fish id and image id

```
tnn3dpf <- tnn3dpf |>
  mutate(fish_id = paste(fish, date, genotype, sep = "_"),
         image_id = paste(fish_id, image, sep = "_image"))
```

Counts

```
tnn3dpf |>
  group_by(genotype) |>
  summarize(n_fish = n_distinct(fish_id),
            n_images = n_distinct(image_id),
            n_nerve = n())
# A tibble: 3 × 4
  genotype n_fish n_images n_nerve
  <chr>     <int>    <int>   <int>
1 het          27       81     305
2 mut          17       51     185
3 wt           14       42     155
```

Put genotype in order: WT, HET, MUT

```
tnn3dpf <- tnn3dpf |>
  mutate(genotype = factor(genotype, levels = c("wt", "het", "mut")))
```

## 1. Analyze period

Period is the distance between nerve exits, so if the mutation is disrupting nerve placement, there may be a difference in average period.

Include random effect of image and of larva

```
tnn3dpf |>
  ggplot(aes(period)) +
  geom_density()
```

```
tnn3dpf |>
  ggplot(aes(period, linetype = genotype)) +
  geom_density()
```

They do not look different at all

Ugly but try some LMMs anyway. I’ll try one with a random effect of both image and fish and a second with just the random effect of fish to see if including the image is important.

```
per3mod1 <- lmer(period ~ genotype + (1|image_id) + (1|fish_id),
                   data = tnn3dpf)

per3mod2 <- lmer(period ~ genotype + (1|fish_id),
                   data = tnn3dpf)

anova(per3mod1, per3mod2)
Data: tnn3dpf
Models:
per3mod2: period ~ genotype + (1 | fish_id)
per3mod1: period ~ genotype + (1 | image_id) + (1 | fish_id)
         npar    AIC    BIC  logLik -2*log(L)  Chisq Df Pr(>Chisq)
per3mod2    5 3928.2 3949.0 -1959.1    3918.2
per3mod1    6 3900.7 3925.6 -1944.3    3888.7 29.538  1  5.483e-08 ***
---
Signif. codes:  0 '***' 0.001 '**' 0.01 '*' 0.05 '.' 0.1 ' ' 1
```

The better model includes the random effect of image and fish. Take a look at that model

```
anova(per3mod1)
Type III Analysis of Variance Table with Satterthwaite's method
         Sum Sq Mean Sq NumDF  DenDF F value Pr(>F)
genotype 58.477  29.238     2 56.413  0.1712 0.8431
summary(per3mod1)
Linear mixed model fit by REML. t-tests use Satterthwaite's method [
lmerModLmerTest]
Formula: period ~ genotype + (1 | image_id) + (1 | fish_id)
   Data: tnn3dpf

REML criterion at convergence: 3880

Scaled residuals:
    Min      1Q  Median      3Q     Max
-3.7662 -0.1696  0.1883  0.4577  1.8507

Random effects:
 Groups   Name        Variance Std.Dev.
 image_id (Intercept)  63.37    7.960
 fish_id  (Intercept)  11.91    3.452
 Residual             170.83   13.070
Number of obs: 471, groups:  image_id, 174; fish_id, 58

Fixed effects:
            Estimate Std. Error      df t value Pr(>|t|)
(Intercept)  79.1506     1.9789 56.3164  39.996   <2e-16 ***
genotypehet   0.1547     2.4353 56.0026   0.064    0.950
genotypemut   1.3477     2.6781 56.7582   0.503    0.617
---
Signif. codes:  0 '***' 0.001 '**' 0.01 '*' 0.05 '.' 0.1 ' ' 1

Correlation of Fixed Effects:
            (Intr) gntyph
genotypehet -0.813
genotypemut -0.739  0.600
```

There is no significant difference by genotype

Retrieve the emmeans

```
emmeans::emmeans(per3mod1, specs = "genotype")
 genotype emmean   SE   df lower.CL upper.CL
 wt         79.2 1.98 55.0     75.2     83.1
 het        79.3 1.42 54.1     76.5     82.2
 mut        80.5 1.80 56.0     76.9     84.1

Degrees-of-freedom method: kenward-roger
Confidence level used: 0.95
pairs(emmeans::emmeans(per3mod1, specs = "genotype"))
 contrast  estimate   SE   df t.ratio p.value
 wt - het    -0.155 2.44 54.7  -0.063  0.9978
 wt - mut    -1.348 2.68 55.4  -0.503  0.8701
 het - mut   -1.193 2.30 55.2  -0.519  0.8622

Degrees-of-freedom method: kenward-roger
P value adjustment: tukey method for comparing a family of 3 estimates
```

### Plot

Show the average for each fish as the raw data and the emmeans with 95% CI as the summary stat

First generate the average period for each fish

```
fish3 <- tnn3dpf |>
  group_by(genotype, fish_id) |>
  summarize(avg_per = mean(period, na.rm = T),
            .groups = "drop")
```

Save the emmeans as a dataset

```
per3mod1_emm <- emmeans::emmeans(per3mod1, specs = "genotype") |>
  as_tibble()
```

Now plot

```
plot_tnn_3dpf_period <- fish3 |>
  ggplot(aes(genotype, avg_per)) +
  geom_jitter(width = .2,
              color = "gray60") +
  geom_crossbar(data = per3mod1_emm,
                aes(genotype, y = emmean,
                    ymin = lower.CL,
                    ymax = upper.CL),
                width = .4) +
  labs(y = "average periodicty (per larvae)") +
  theme_classic()

plot_tnn_3dpf_period
```

```
ggsave(plot_tnn_3dpf_period,
       filename = "plots/tnn_3dpf_period.tiff",
       height = 4, width = 5)
```

## 2. Analyze variance of period

Because we see a difference in the variance of the period, we can analyze that directly.

First create the variance of the period for each image

```
var_per3 <- tnn3dpf |>
  group_by(genotype, fish_id, image_id) |>
  summarize(var_per = var(period, na.rm = T),
            .groups = "drop")
```

See these variances

```
var_per3 |>
  ggplot(aes(var_per, linetype = genotype)) +
  geom_density()
```

```
var_per3 |>
  ggplot(aes(genotype, var_per)) +
  geom_jitter(width = .2)
```

This time the het looks different from the other two.

How many images and fish per genotype?

```
# number of images per genotype
var_per3 |>
  ungroup() |>
  count(genotype)
# A tibble: 3 × 2
  genotype     n
  <fct>    <int>
1 wt          42
2 het         81
3 mut         51
# number of fish per genotype
var_per3 |>
  ungroup() |>
  group_by(genotype) |>
  summarize(n_fish = n_distinct(fish_id))
# A tibble: 3 × 2
  genotype n_fish
  <fct>     <int>
1 wt           14
2 het          27
3 mut          17
```

Then model using log-normal and gamma regression

```
var3mod1 <- glmer(var_per ~ genotype + (1|fish_id),
              data = var_per3,
              family = gaussian(link = "log"))

var3mod2 <- glmer(var_per ~ genotype + (1|fish_id),
              data = var_per3,
              family = Gamma(link = "log"))
```

I researched this failure to converge message and it seems that this one is a false positive. We can use this model and it fits so much better than the log-normal

```
# run the model using all available optimizers
gm_all <- allFit(var3mod2, maxfun = 1e5)

# Check which optimizers converged successfully
convergence_status <- sapply(gm_all, inherits, "try-error")
which_ok <- !convergence_status
names(gm_all)[which_ok]

# see that they all DID converge!

# Extract and compare specific the Log-likelihoods.
# higher is generally better, but they should be similar if they converged correctly

sapply(gm_all[which_ok], logLik)

# yes, they are similar
```

Check the fit of the log-normal and the fit of the Gamma

```
performance::check_predictions(var3mod1)
Warning: Maximum value of original data is not included in the
  replicated data.
  Model may not capture the variation of the data.
```

performance::check_predictions(var3mod2)

See that the Gamma fits the raw data so much better than the log-normal. The Gamma is by far the better model!

```
summary(var3mod2)
Generalized linear mixed model fit by maximum likelihood (Laplace
  Approximation) [glmerMod]
 Family: Gamma  ( log )
Formula: var_per ~ genotype + (1 | fish_id)
   Data: var_per3

      AIC       BIC    logLik -2*log(L)  df.resid
   1813.1    1828.9    -901.6    1803.1       169

Scaled residuals:
    Min      1Q  Median      3Q     Max
-0.8190 -0.7360 -0.3477  0.5013  2.3043

Random effects:
 Groups  Name        Variance Std.Dev.
 fish_id (Intercept) 1.924    1.387
Number of obs: 174, groups:  fish_id, 58

Fixed effects:
             Estimate Std. Error t value Pr(>|z|)
(Intercept)  3.753632   0.004699  798.79   <2e-16 ***
genotypehet  0.262525   0.004706   55.78   <2e-16 ***
genotypemut -0.123419   0.004734  -26.07   <2e-16 ***
---
Signif. codes:  0 '***' 0.001 '**' 0.01 '*' 0.05 '.' 0.1 ' ' 1

Correlation of Fixed Effects:
            (Intr) gntyph
genotypehet -0.007
genotypemut -0.001 -0.001
optimizer (Nelder_Mead) convergence code: 0 (OK)
Model failed to converge with max|grad| = 0.0151016 (tol = 0.002, component 1)
  See ?lme4::convergence and ?lme4::troubleshooting.
```

See the predicted variance for each genotype

```
emmeans::emmeans(var3mod2, specs = "genotype", type = "response")
 genotype response    SE  df asymp.LCL asymp.UCL
 wt           42.7 0.201 Inf      42.3      43.1
 het          55.5 0.368 Inf      54.8      56.2
 mut          37.7 0.252 Inf      37.2      38.2

Confidence level used: 0.95
Intervals are back-transformed from the log scale
pairs(emmeans::emmeans(var3mod2, specs = "genotype"))
 contrast  estimate      SE  df z.ratio p.value
 wt - het    -0.263 0.00471 Inf -55.780 <0.0001
 wt - mut     0.123 0.00473 Inf  26.070 <0.0001
 het - mut    0.386 0.00668 Inf  57.795 <0.0001

Results are given on the log (not the response) scale.
P value adjustment: tukey method for comparing a family of 3 estimates
```

Het has highest variance, then wt then mut. All 3 comparisons are statistically significant

### Plot

First generate the variance of the period for each fish

```
fishvar3 <- tnn3dpf |>
  group_by(fish_id) |>
  fill(ectopic, .direction = "updown") |>
  ungroup() |>
  group_by(genotype, fish_id, ectopic) |>
  summarize(var_per = var(period, na.rm = T),
            .groups = "drop") |>
  mutate(ectopic = if_else(is.na(ectopic), 0, ectopic),
         ectopic = factor(ectopic))
```

Save the emmeans as a dataset

```
var3mod2_emm <- emmeans::emmeans(var3mod2, specs = "genotype", type = "response") |>
  as_tibble()
```

Now plot

```
plot_tnn_3dpf_variance <- fishvar3 |>
  ggplot(aes(genotype, var_per)) +
  geom_jitter(width = .2,
              color = "gray60",
              aes(shape = ectopic)) +
  geom_crossbar(data = var3mod2_emm,
                aes(genotype, y = response,
                    ymin = asymp.LCL,
                    ymax = asymp.UCL),
                width = .4) +
  labs(y = "variance of the periodicty (per larvae)") +
  theme_classic() +
  theme(legend.position = "none")

plot_tnn_3dpf_variance
```

```
ggsave(plot_tnn_3dpf_variance,
       filename = "plots/tnn_3dpf_variance.tiff",
       height = 4, width = 5)
```

## 3. Analyze number of terminal points

Number of terminal points is counted for each image, so we will need a random effect for each fish. We should account for the fact that some nerves are longer so they would have more terminal points, so add total filament length (length of nerve) as an offset

First load these data

tnntrace3dpf <- read_csv("csvs_MJ/3dpf_tnn_tracing_final.csv")

Make a unique fish ID and an image ID

```
tnntrace3dpf <- tnntrace3dpf |>
  mutate(fish_id = paste(fish, date, genotype, sep = "_"),
         image_id = paste(fish_id, image, sep = "_image"))
```

Put genotype in order: WT, HET, MUT

```
tnntrace3dpf <- tnntrace3dpf |>
  mutate(genotype = factor(genotype, levels = c("wt", "het", "mut")))
```

Look at raw number of terminal points by genotype. This ignores the nesting

```
tnntrace3dpf |>
  ggplot(aes(genotype, terminal_pts)) +
  geom_jitter(width = .2)
```

Model using Poisson taking into account the main_distance of each nerve

```
term3mod1 <- glmer(terminal_pts ~ genotype + (1|fish_id),
                   family = poisson,
                   offset = log(main_distance),
                   data = tnntrace3dpf)

summary(term3mod1)
Generalized linear mixed model fit by maximum likelihood (Laplace
  Approximation) [glmerMod]
 Family: poisson  ( log )
Formula: terminal_pts ~ genotype + (1 | fish_id)
   Data: tnntrace3dpf
 Offset: log(main_distance)

      AIC       BIC    logLik -2*log(L)  df.resid
    629.6     639.1    -310.8     621.6        75

Scaled residuals:
    Min      1Q  Median      3Q     Max
-3.6402 -0.5962  0.1088  0.6330  1.7009

Random effects:
 Groups  Name        Variance Std.Dev.
 fish_id (Intercept) 0.005378 0.07333
Number of obs: 79, groups:  fish_id, 36

Fixed effects:
            Estimate Std. Error z value Pr(>|z|)
(Intercept) -0.37632    0.03316 -11.350   <2e-16 ***
genotypehet -0.06806    0.04154  -1.639    0.101
genotypemut -0.06939    0.04600  -1.509    0.131
---
Signif. codes:  0 '***' 0.001 '**' 0.01 '*' 0.05 '.' 0.1 ' ' 1

Correlation of Fixed Effects:
            (Intr) gntyph
genotypehet -0.798
genotypemut -0.722  0.576
```

See how many terminal points each genotype is predicted by this model to have

```
emmeans::emmeans(term3mod1, specs = "genotype", type = "response")
 genotype rate   SE  df asymp.LCL asymp.UCL
 wt        109 3.62 Inf     102.4       117
 het       102 2.56 Inf      97.2       107
 mut       102 3.24 Inf      95.8       109

Confidence level used: 0.95
Intervals are back-transformed from the log scale
pairs(emmeans::emmeans(term3mod1, specs = "genotype"))
 contrast  estimate     SE  df z.ratio p.value
 wt - het   0.06806 0.0415 Inf   1.639  0.2295
 wt - mut   0.06939 0.0460 Inf   1.509  0.2868
 het - mut  0.00133 0.0405 Inf   0.033  0.9994

Results are given on the log (not the response) scale.
P value adjustment: tukey method for comparing a family of 3 estimates
```

For the average main_distance, WT is expected to have 109 terminal points, while het has 102 and mut has 102

### Plot

This plot is on the image level

Save the emmeans as a dataset

```
term3mod1_emm <- emmeans::emmeans(term3mod1, specs = "genotype",
                                 type = "response") |>
  as_tibble()
```

Now plot

```
plot_tnn_3dpf_termpts <- tnntrace3dpf |>
  ggplot(aes(genotype, terminal_pts)) +
  geom_jitter(width = .2,
              color = "gray60") +
  geom_crossbar(data = term3mod1_emm,
                aes(genotype, y = rate,
                    ymin = asymp.LCL,
                    ymax = asymp.UCL),
                width = .4) +
  labs(y = "number of terminal points (per image)") +
  theme_classic()

plot_tnn_3dpf_termpts
```

```
ggsave(plot_tnn_3dpf_termpts,
       filename = "plots/tnn_3dpf_termpts.tiff",
       height = 4, width = 5)
```

## 4. Analyze total filament length

Random effect for each fish

See a plot

```
tnntrace3dpf |>
  ggplot(aes(genotype, main_distance)) +
  geom_jitter(width = .2)
```

Now model

```
dist3mod1 <- lmer(main_distance ~ genotype + (1|fish_id),
                   data = tnntrace3dpf)
summary(dist3mod1)
Linear mixed model fit by REML. t-tests use Satterthwaite's method [
lmerModLmerTest]
Formula: main_distance ~ genotype + (1 | fish_id)
   Data: tnntrace3dpf

REML criterion at convergence: 658

Scaled residuals:
    Min      1Q  Median      3Q     Max
-1.8933 -0.6410  0.3036  0.6141  1.6643

Random effects:
 Groups   Name        Variance Std.Dev.
 fish_id  (Intercept) 165.7    12.87
 Residual             190.9    13.82
Number of obs: 79, groups:  fish_id, 36

Fixed effects:
            Estimate Std. Error      df t value Pr(>|t|)
(Intercept)  156.848      5.378  32.665  29.163   <2e-16 ***
genotypehet    9.781      6.734  32.931   1.453    0.156
genotypemut   -2.304      7.359  34.352  -0.313    0.756
---
Signif. codes:  0 '***' 0.001 '**' 0.01 '*' 0.05 '.' 0.1 ' ' 1

Correlation of Fixed Effects:
            (Intr) gntyph
genotypehet -0.799
genotypemut -0.731  0.584
```

See the predicted means

```
emmeans::emmeans(dist3mod1, specs = "genotype")
 genotype emmean   SE   df lower.CL upper.CL
 wt          157 5.40 30.1      146      168
 het         167 4.06 30.9      158      175
 mut         155 5.04 33.8      144      165

Degrees-of-freedom method: kenward-roger
Confidence level used: 0.95
pairs(emmeans::emmeans(dist3mod1, specs = "genotype"))
 contrast  estimate   SE   df t.ratio p.value
 wt - het     -9.78 6.75 30.4  -1.448  0.3297
 wt - mut      2.30 7.38 31.8   0.312  0.9478
 het - mut    12.09 6.48 32.6   1.866  0.1646

Degrees-of-freedom method: kenward-roger
P value adjustment: tukey method for comparing a family of 3 estimates
```

The het has a slightly longer main filament, but the differnences are not statistically significant

### Plot

This plot is on the image level

Save the emmeans as a dataset

```
dist3mod1_emm <- emmeans::emmeans(dist3mod1, specs = "genotype") |>
  as_tibble()
```

Now plot

```
plot_tnn_3dpf_length <- tnntrace3dpf |>
  ggplot(aes(genotype, main_distance)) +
  geom_jitter(width = .2,
              color = "gray60") +
  geom_crossbar(data = dist3mod1_emm,
                aes(genotype, y = emmean,
                    ymin = lower.CL,
                    ymax = upper.CL),
                width = .4) +
  labs(y = "dorsal to ventral filament length (per image)") +
  theme_classic()

plot_tnn_3dpf_length
```

```
ggsave(plot_tnn_3dpf_length,
       filename = "plots/tnn_3dpf_length.tiff",
       height = 4, width = 5)
```

# TNC crispant+TNN at 3dpf

Load the TNC crispant + TNN 3dpf data file

tnc3dpf <- read_csv("csvs_MJ/3dpf_tnn_tnc_crispant_periodicity.csv")

Create unique fish id and image id

```
tnc3dpf <- tnc3dpf |>
  mutate(fish_id = paste(fish_number, date, genotype, sep = "_"),
         image_id = paste(fish_id, image, sep = "_image"))
```

Put genotype in order: HET, MUT

```
tnc3dpf <- tnc3dpf |>
  mutate(genotype = factor(genotype, levels = c("het_crisp", "mut_crisp")))
```

## 1. Analyze period

Period is the distance between nerve exits, so if the mutation is disrupting nerve placement, there may be a difference in average period.

Include random effect of image and of larva

```
tnc3dpf |>
  ggplot(aes(period)) +
  geom_density()
```

```
tnc3dpf |>
  ggplot(aes(period, linetype = genotype)) +
  geom_density()
```

They do not look much different

Ugly but try some LMMs anyway. I’ll try one with a random effect of both image and fish and a second with just the random effect of fish to see if including the image is important.

```
per3tncmod1 <- lmer(period ~ genotype + (1|image_id) + (1|fish_id),
                   data = tnc3dpf)

per3tncmod2 <- lmer(period ~ genotype + (1|fish_id),
                   data = tnc3dpf)

anova(per3tncmod1, per3tncmod2)
Data: tnc3dpf
Models:
per3tncmod2: period ~ genotype + (1 | fish_id)
per3tncmod1: period ~ genotype + (1 | image_id) + (1 | fish_id)
            npar    AIC    BIC  logLik -2*log(L)  Chisq Df Pr(>Chisq)
per3tncmod2    4 1383.2 1395.6 -687.60    1375.2
per3tncmod1    5 1378.4 1393.9 -684.22    1368.4 6.7742  1   0.009249 **
---
Signif. codes:  0 '***' 0.001 '**' 0.01 '*' 0.05 '.' 0.1 ' ' 1
```

The better model includes the random effect of image and fish. Take a look at that model

```
anova(per3tncmod1)
Type III Analysis of Variance Table with Satterthwaite's method
         Sum Sq Mean Sq NumDF  DenDF F value  Pr(>F)
genotype 648.94  648.94     1 65.832  3.0513 0.08534 .
---
Signif. codes:  0 '***' 0.001 '**' 0.01 '*' 0.05 '.' 0.1 ' ' 1
summary(per3tncmod1)
Linear mixed model fit by REML. t-tests use Satterthwaite's method [
lmerModLmerTest]
Formula: period ~ genotype + (1 | image_id) + (1 | fish_id)
   Data: tnc3dpf

REML criterion at convergence: 1361.7

Scaled residuals:
    Min      1Q  Median      3Q     Max
-3.8578 -0.1683  0.1433  0.4829  1.4411

Random effects:
 Groups   Name        Variance Std.Dev.
 image_id (Intercept)  60.15    7.756
 fish_id  (Intercept)   0.00    0.000
 Residual             212.68   14.584
Number of obs: 163, groups:  image_id, 63; fish_id, 21

Fixed effects:
                  Estimate Std. Error     df t value Pr(>|t|)
(Intercept)         75.211      2.019 67.936  37.256   <2e-16 ***
genotypemut_crisp    5.334      3.053 65.832   1.747   0.0853 .
---
Signif. codes:  0 '***' 0.001 '**' 0.01 '*' 0.05 '.' 0.1 ' ' 1

Correlation of Fixed Effects:
            (Intr)
gntypmt_crs -0.661
optimizer (nloptwrap) convergence code: 0 (OK)
boundary (singular) fit: see help('isSingular')
```

mut_crisp has a slightly higher average period, but the difference is not quite statistically significant

Retrieve the emmeans

```
emmeans::emmeans(per3tncmod1, specs = "genotype")
 genotype  emmean   SE   df lower.CL upper.CL
 het_crisp   75.2 2.02 19.5     71.0     79.4
 mut_crisp   80.5 2.29 18.1     75.7     85.4

Degrees-of-freedom method: kenward-roger
Confidence level used: 0.95
pairs(emmeans::emmeans(per3tncmod1, specs = "genotype"))
 contrast              estimate   SE   df t.ratio p.value
 het_crisp - mut_crisp    -5.33 3.06 18.7  -1.744  0.0976

Degrees-of-freedom method: kenward-roger
```

### Plot

Show the average for each fish as the raw data and the emmeans with 95% CI as the summary stat

First generate the average period for each fish

```
fish3tnc <- tnc3dpf |>
  group_by(genotype, fish_id) |>
  summarize(avg_per = mean(period, na.rm = T),
            .groups = "drop")
```

Save the emmeans as a dataset

```
per3tncmod1_emm <- emmeans::emmeans(per3tncmod1, specs = "genotype") |>
  as_tibble()
```

Now plot

```
plot_tnc_3dpf_period <- fish3tnc |>
  ggplot(aes(genotype, avg_per)) +
  geom_jitter(width = .2,
              color = "gray60") +
  geom_crossbar(data = per3tncmod1_emm,
                aes(genotype, y = emmean,
                    ymin = lower.CL,
                    ymax = upper.CL),
                width = .4) +
  labs(y = "average periodicty (per larvae)") +
  theme_classic()

plot_tnc_3dpf_period
```

```
ggsave(plot_tnc_3dpf_period,
       filename = "plots/tnc_3dpf_period.tiff",
       height = 4, width = 5)
```

## 2. Analyze variance of period

Because we might see a difference in the variance of the period, we can analyze that directly.

First create the variance of the period for each image

```
var_per3tnc <- tnc3dpf |>
  group_by(genotype, fish_id, image_id) |>
  summarize(var_per = var(period, na.rm = T),
            .groups = "drop")
```

See these variances

```
var_per3tnc |>
  ggplot(aes(var_per, linetype = genotype)) +
  geom_density()
```

```
var_per3tnc |>
  ggplot(aes(genotype, var_per)) +
  geom_jitter(width = .2)
```

The het_crisp has higher variance in the period than the mut_crisp

Then model using gamma regression

```
var3tncmod2 <- glmer(var_per ~ genotype + (1|fish_id),
              data = var_per3tnc,
              family = Gamma(link = "log"))
```

Again the failure to converge is a false positive for this gamma model. We can use this model

```
summary(var3tncmod2)
Generalized linear mixed model fit by maximum likelihood (Laplace
  Approximation) [glmerMod]
 Family: Gamma  ( log )
Formula: var_per ~ genotype + (1 | fish_id)
   Data: var_per3tnc

      AIC       BIC    logLik -2*log(L)  df.resid
    688.2     696.8    -340.1     680.2        59

Scaled residuals:
    Min      1Q  Median      3Q     Max
-0.7496 -0.7038 -0.4267  0.3974  2.3318

Random effects:
 Groups  Name        Variance Std.Dev.
 fish_id (Intercept) 1.515    1.231
Number of obs: 63, groups:  fish_id, 21

Fixed effects:
                   Estimate Std. Error t value Pr(>|z|)
(Intercept)        4.554095   0.003945  1154.5   <2e-16 ***
genotypemut_crisp -0.862468   0.003934  -219.2   <2e-16 ***
---
Signif. codes:  0 '***' 0.001 '**' 0.01 '*' 0.05 '.' 0.1 ' ' 1

Correlation of Fixed Effects:
            (Intr)
gntypmt_crs 0.003
optimizer (Nelder_Mead) convergence code: 0 (OK)
Model failed to converge with max|grad| = 0.0180053 (tol = 0.002, component 1)
  See ?lme4::convergence and ?lme4::troubleshooting.
```

See the predicted variance for each genotype

```
emmeans::emmeans(var3tncmod2, specs = "genotype", type = "response")
 genotype  response    SE  df asymp.LCL asymp.UCL
 het_crisp     95.0 0.375 Inf      94.3      95.8
 mut_crisp     40.1 0.224 Inf      39.7      40.6

Confidence level used: 0.95
Intervals are back-transformed from the log scale
pairs(emmeans::emmeans(var3tncmod2, specs = "genotype"))
 contrast              estimate      SE  df z.ratio p.value
 het_crisp - mut_crisp    0.862 0.00393 Inf 219.219 <0.0001

Results are given on the log (not the response) scale.
```

Het has much higher variance than mut

### Plot

First generate the variance of the period for each fish

```
fishvar3tnc <- tnc3dpf |>
  group_by(fish_id) |>
  fill(ectopic, .direction = "updown") |>
  ungroup() |>
  group_by(genotype, fish_id, ectopic) |>
  summarize(var_per = var(period, na.rm = T),
            .groups = "drop") |>
  mutate(ectopic = if_else(is.na(ectopic), 0, ectopic),
         ectopic = factor(ectopic))
```

Save the emmeans as a dataset

```
var3tncmod2_emm <- emmeans::emmeans(var3tncmod2, specs = "genotype", type = "response") |>
  as_tibble()
```

Now plot

```
plot_tnc_3dpf_variance <- fishvar3tnc |>
  ggplot(aes(genotype, var_per)) +
  geom_jitter(width = .2,
              color = "gray60",
              aes(shape = ectopic)) +
  geom_crossbar(data = var3tncmod2_emm,
                aes(genotype, y = response,
                    ymin = asymp.LCL,
                    ymax = asymp.UCL),
                width = .4) +
  labs(y = "variance of the periodicty (per larvae)") +
  theme_classic() +
  theme(legend.position = "none")

plot_tnc_3dpf_variance
```

```
ggsave(plot_tnc_3dpf_variance,
       filename = "plots/tnc_3dpf_variance.tiff",
       height = 4, width = 5)
```

# TNN crispant at 3dpf

Load the TNN crispant 3dpf data file

tnncrisp3dpf <- read_csv("csvs_MJ/3dpf_tnn_crisp_periodicity.csv")

Create unique fish id and image id

```
tnncrisp3dpf <- tnncrisp3dpf |>
  mutate(fish_id = paste(fish, date, genotype, sep = "_"),
         image_id = paste(fish_id, image, sep = "_image"))
```

## 1. Analyze period

Period is the distance between nerve exits, so if the mutation is disrupting nerve placement, there may be a difference in average period.

Include random effect of image and of larva

```
tnncrisp3dpf |>
  ggplot(aes(periodicity)) +
  geom_density()
```

```
tnncrisp3dpf |>
  ggplot(aes(periodicity, linetype = genotype)) +
  geom_density()
```

They do not look different at all

Try some LMMs. I’ll try one with a random effect of both image and fish and a second with just the random effect of fish to see if including the image is important.

```
percrisp3mod1 <- lmer(periodicity ~ genotype +
                        (1|image_id) + (1|fish_id),
                   data = tnncrisp3dpf)

percrisp3mod2 <- lmer(periodicity ~ genotype + (1|fish_id),
                   data = tnncrisp3dpf)

anova(percrisp3mod1, percrisp3mod2)
Data: tnncrisp3dpf
Models:
percrisp3mod2: periodicity ~ genotype + (1 | fish_id)
percrisp3mod1: periodicity ~ genotype + (1 | image_id) + (1 | fish_id)
              npar    AIC    BIC  logLik -2*log(L)  Chisq Df Pr(>Chisq)
percrisp3mod2    4 2731.2 2746.6 -1361.6    2723.2
percrisp3mod1    5 2713.1 2732.4 -1351.6    2703.1 20.058  1  7.515e-06 ***
---
Signif. codes:  0 '***' 0.001 '**' 0.01 '*' 0.05 '.' 0.1 ' ' 1
```

The better model includes the random effect of image and fish. Take a look at that model

```
anova(percrisp3mod1)
Type III Analysis of Variance Table with Satterthwaite's method
         Sum Sq Mean Sq NumDF  DenDF F value Pr(>F)
genotype 5.9167  5.9167     1 35.284   0.058 0.8111
summary(percrisp3mod1)
Linear mixed model fit by REML. t-tests use Satterthwaite's method [
lmerModLmerTest]
Formula: periodicity ~ genotype + (1 | image_id) + (1 | fish_id)
   Data: tnncrisp3dpf

REML criterion at convergence: 2698.8

Scaled residuals:
    Min      1Q  Median      3Q     Max
-5.4434 -0.2986  0.0481  0.4326  2.2525

Random effects:
 Groups   Name        Variance Std.Dev.
 image_id (Intercept)  33.537   5.791
 fish_id  (Intercept)   4.405   2.099
 Residual             102.055  10.102
Number of obs: 351, groups:  image_id, 111; fish_id, 37

Fixed effects:
                 Estimate Std. Error      df t value Pr(>|t|)
(Intercept)       81.5471     1.2086 34.8397  67.473   <2e-16 ***
genotypecrispant  -0.4073     1.6915 35.2838  -0.241    0.811
---
Signif. codes:  0 '***' 0.001 '**' 0.01 '*' 0.05 '.' 0.1 ' ' 1

Correlation of Fixed Effects:
            (Intr)
gntypcrspnt -0.714
```

There is no significant difference by genotype

Retrieve the emmeans

```
emmeans::emmeans(percrisp3mod1, specs = "genotype")
 genotype emmean   SE   df lower.CL upper.CL
 control    81.5 1.21 34.5     79.1     84.0
 crispant   81.1 1.18 35.4     78.7     83.5

Degrees-of-freedom method: kenward-roger
Confidence level used: 0.95
pairs(emmeans::emmeans(percrisp3mod1, specs = "genotype"))
 contrast           estimate   SE   df t.ratio p.value
 control - crispant    0.407 1.69 34.9   0.241  0.8112

Degrees-of-freedom method: kenward-roger
```

### Plot

Show the average for each fish as the raw data and the emmeans with 95% CI as the summary stat

First generate the average period for each fish

```
fish3crisp <- tnncrisp3dpf |>
  group_by(genotype, fish_id) |>
  summarize(avg_per = mean(periodicity, na.rm = T),
            .groups = "drop")
```

Save the emmeans as a dataset

```
percrisp3mod1_emm <- emmeans::emmeans(percrisp3mod1, specs = "genotype") |>
  as_tibble()
```

Now plot

```
plot_tnncrisp_3dpf_period <- fish3crisp |>
  ggplot(aes(genotype, avg_per)) +
  geom_jitter(width = .2,
              color = "gray60") +
  geom_crossbar(data = percrisp3mod1_emm,
                aes(genotype, y = emmean,
                    ymin = lower.CL,
                    ymax = upper.CL),
                width = .4) +
  labs(y = "average periodicty (per larvae)") +
  theme_classic()

plot_tnncrisp_3dpf_period
```

```
ggsave(plot_tnncrisp_3dpf_period,
       filename = "plots/tnncrisp_3dpf_period.tiff",
       height = 4, width = 5)
```

## 2. Analyze variance of period

Because we may see a difference in the variance of the period, we can analyze that directly.

First create the variance of the period for each image

```
var_per3crisp <- tnncrisp3dpf |>
  group_by(genotype, fish_id, image_id) |>
  summarize(var_per = var(periodicity, na.rm = T),
            .groups = "drop")
```

See these variances

```
var_per3crisp |>
  ggplot(aes(var_per, linetype = genotype)) +
  geom_density()
```

```
var_per3crisp |>
  ggplot(aes(genotype, var_per)) +
  geom_jitter(width = .2)
```

The crispant has slightly higher variance in the period than the control.

Then model using gamma regression

```
var3crispmod2 <- glmer(var_per ~ genotype + (1|fish_id),
              data = var_per3crisp,
              family = Gamma(link = "log"))
```

The gamma model fits much better

```
summary(var3crispmod2)
Generalized linear mixed model fit by maximum likelihood (Laplace
  Approximation) [glmerMod]
 Family: Gamma  ( log )
Formula: var_per ~ genotype + (1 | fish_id)
   Data: var_per3crisp

      AIC       BIC    logLik -2*log(L)  df.resid
   1129.6    1140.4    -560.8    1121.6       107

Scaled residuals:
    Min      1Q  Median      3Q     Max
-0.8295 -0.6700 -0.3094  0.3706  2.9920

Random effects:
 Groups  Name        Variance Std.Dev.
 fish_id (Intercept) 1.212    1.101
Number of obs: 111, groups:  fish_id, 37

Fixed effects:
                 Estimate Std. Error t value Pr(>|z|)
(Intercept)      3.538708   0.005974  592.34   <2e-16 ***
genotypecrispant 0.225573   0.005997   37.61   <2e-16 ***
---
Signif. codes:  0 '***' 0.001 '**' 0.01 '*' 0.05 '.' 0.1 ' ' 1

Correlation of Fixed Effects:
            (Intr)
gntypcrspnt -0.001
optimizer (Nelder_Mead) convergence code: 0 (OK)
Model failed to converge with max|grad| = 0.0117035 (tol = 0.002, component 1)
  See ?lme4::convergence and ?lme4::troubleshooting.
```

See the predicted variance for each genotype

```
emmeans::emmeans(var3crispmod2, specs = "genotype", type = "response")
 genotype response    SE  df asymp.LCL asymp.UCL
 control      34.4 0.206 Inf      34.0      34.8
 crispant     43.1 0.365 Inf      42.4      43.9

Confidence level used: 0.95
Intervals are back-transformed from the log scale
pairs(emmeans::emmeans(var3crispmod2, specs = "genotype"))
 contrast           estimate    SE  df z.ratio p.value
 control - crispant   -0.226 0.006 Inf -37.613 <0.0001

Results are given on the log (not the response) scale.
```

Crispant has higher variance but not statistically significantly

### Plot

First generate the variance of the period for each fish

```
fishvar3crisp <- tnncrisp3dpf |>
  group_by(fish_id) |>
  fill(ectopic, .direction = "updown") |>
  ungroup() |>
  group_by(genotype, fish_id, ectopic) |>
  summarize(var_per = var(periodicity, na.rm = T),
            .groups = "drop") |>
  mutate(ectopic = if_else(is.na(ectopic), 0, ectopic),
         ectopic = factor(ectopic))
```

Save the emmeans as a dataset

```
var3crispmod2_emm <- emmeans::emmeans(var3crispmod2, specs = "genotype", type = "response") |>
  as_tibble()
```

Now plot.

Note there is one fish in the control group (fish4_4112025_control) that was not labeled as ectopic but there IS an ectopic nerve there, so change that group label.

```
plot_tnncrisp_3dpf_variance <- fishvar3crisp |>
  mutate(ectopic = if_else(ectopic == 0, "no", "yes")) |>
  mutate(ectopic = if_else(fish_id == "fish4_4112025_control",
                           "yes",
                           ectopic)) |>
  ggplot(aes(genotype, var_per)) +
  geom_jitter(width = .2,
              color = "gray60",
              aes(shape = ectopic)) +
  geom_crossbar(data = var3crispmod2_emm,
                aes(genotype, y = response,
                    ymin = asymp.LCL,
                    ymax = asymp.UCL),
                width = .4) +
  labs(y = "variance of the periodicty (per larvae)") +
  theme_classic() +
  theme(legend.position = "none")

plot_tnncrisp_3dpf_variance
```

```
ggsave(plot_tnncrisp_3dpf_variance,
       filename = "plots/tnncrisp_3dpf_variance.tiff",
       height = 4, width = 5)
```

## 3. Analyze number of terminal points

Number of terminal points is counted for each image, so we will need a random effect for each fish. We should account for the fact that some nerves are longer so they would have more terminal points, so add total filament length (length of nerve) as an offset

First load these data

crisptrace3dpf <- read_csv("csvs_MJ/3dpf_tnn_crisp_tracing.csv")

Make a unique fish ID and an image ID

```
crisptrace3dpf <- crisptrace3dpf |>
  mutate(fish_id = paste(fish, date, genotype, sep = "_"),
         image_id = paste(fish_id, image, sep = "_image"))
```

Look at raw number of terminal points by genotype. This ignores the nesting

```
crisptrace3dpf |>
  ggplot(aes(genotype, segment_terminal_pts)) +
  geom_jitter(width = .2)
```

Model using Poisson taking into account the main_distance of each nerve

```
term3crispmod1 <- glmer(segment_terminal_pts ~ genotype + (1|fish_id),
                   family = poisson,
                   offset = log(main_distance),
                   data = crisptrace3dpf)

summary(term3crispmod1)
Generalized linear mixed model fit by maximum likelihood (Laplace
  Approximation) [glmerMod]
 Family: poisson  ( log )
Formula: segment_terminal_pts ~ genotype + (1 | fish_id)
   Data: crisptrace3dpf
 Offset: log(main_distance)

      AIC       BIC    logLik -2*log(L)  df.resid
    677.8     685.1    -335.9     671.8        80

Scaled residuals:
    Min      1Q  Median      3Q     Max
-3.4060 -0.3956  0.0517  0.4455  2.2653

Random effects:
 Groups  Name        Variance Std.Dev.
 fish_id (Intercept) 0.0368   0.1918
Number of obs: 83, groups:  fish_id, 30

Fixed effects:
                 Estimate Std. Error z value Pr(>|z|)
(Intercept)      -0.71586    0.05126 -13.965   <2e-16 ***
genotypecrispant  0.04532    0.07486   0.605    0.545
---
Signif. codes:  0 '***' 0.001 '**' 0.01 '*' 0.05 '.' 0.1 ' ' 1

Correlation of Fixed Effects:
            (Intr)
gntypcrspnt -0.685
```

Crispant has significantly more terminal points than control

See how many terminal points each genotype is predicted by this model to have

```
emmeans::emmeans(term3crispmod1, specs = "genotype", type = "response")
 genotype rate   SE  df asymp.LCL asymp.UCL
 control  75.1 3.85 Inf      67.9      83.0
 crispant 78.5 4.28 Inf      70.6      87.4

Confidence level used: 0.95
Intervals are back-transformed from the log scale
pairs(emmeans::emmeans(term3crispmod1, specs = "genotype"))
 contrast           estimate     SE  df z.ratio p.value
 control - crispant  -0.0453 0.0749 Inf  -0.605  0.5449

Results are given on the log (not the response) scale.
```

For the average main_distance, crispant is expected to have 86 terminal points, while control has 72

### Plot

This plot is on the image level

Save the emmeans as a dataset

```
term3crispmod1_emm <- emmeans::emmeans(term3crispmod1,
                                       specs = "genotype",
                                 type = "response") |>
  as_tibble()
```

Now plot

```
plot_tnncrisp_3dpf_termpts <- crisptrace3dpf |>
  ggplot(aes(genotype, segment_terminal_pts)) +
  geom_jitter(width = .2,
              color = "gray60") +
  geom_crossbar(data = term3crispmod1_emm,
                aes(genotype, y = rate,
                    ymin = asymp.LCL,
                    ymax = asymp.UCL),
                width = .4) +
  labs(y = "number of terminal points (per image)") +
  theme_classic()

plot_tnncrisp_3dpf_termpts
```

```
ggsave(plot_tnncrisp_3dpf_termpts,
       filename = "plots/tnncrisp_3dpf_termpts.tiff",
       height = 4, width = 5)
```

## 4. Analyze total filament length

Random effect for each fish

See a plot

```
crisptrace3dpf |>
  ggplot(aes(genotype, main_distance)) +
  geom_jitter(width = .2)
```

Now model

```
dist3crispmod1 <- lmer(main_distance ~ genotype + (1|fish_id),
                   data = crisptrace3dpf)
summary(dist3crispmod1)
Linear mixed model fit by REML. t-tests use Satterthwaite's method [
lmerModLmerTest]
Formula: main_distance ~ genotype + (1 | fish_id)
   Data: crisptrace3dpf

REML criterion at convergence: 686.3

Scaled residuals:
    Min      1Q  Median      3Q     Max
-2.2683 -0.7529  0.1505  0.6947  2.4710

Random effects:
 Groups   Name        Variance Std.Dev.
 fish_id  (Intercept)   0.0     0.00
 Residual             255.6    15.99
Number of obs: 83, groups:  fish_id, 30

Fixed effects:
                 Estimate Std. Error      df t value Pr(>|t|)
(Intercept)       153.082      2.410  81.000  63.517   <2e-16 ***
genotypecrispant    2.772      3.516  81.000   0.788    0.433
---
Signif. codes:  0 '***' 0.001 '**' 0.01 '*' 0.05 '.' 0.1 ' ' 1

Correlation of Fixed Effects:
            (Intr)
gntypcrspnt -0.685
optimizer (nloptwrap) convergence code: 0 (OK)
boundary (singular) fit: see help('isSingular')
```

See the predicted means

```
emmeans::emmeans(dist3crispmod1, specs = "genotype")
 genotype emmean   SE   df lower.CL upper.CL
 control     153 2.42 27.1      148      158
 crispant    156 2.56 26.4      151      161

Degrees-of-freedom method: kenward-roger
Confidence level used: 0.95
pairs(emmeans::emmeans(dist3crispmod1, specs = "genotype"))
 contrast           estimate   SE   df t.ratio p.value
 control - crispant    -2.77 3.52 26.7  -0.787  0.4384

Degrees-of-freedom method: kenward-roger
```

The crispant has a slightly longer main filament, but the differences are not statistically significant

### Plot

This plot is on the image level

Save the emmeans as a dataset

```
dist3crispmod1_emm <- emmeans::emmeans(dist3crispmod1, specs = "genotype") |>
  as_tibble()
```

Now plot

```
plot_tnncrisp_3dpf_length <- crisptrace3dpf |>
  ggplot(aes(genotype, main_distance)) +
  geom_jitter(width = .2,
              color = "gray60") +
  geom_crossbar(data = dist3crispmod1_emm,
                aes(genotype, y = emmean,
                    ymin = lower.CL,
                    ymax = upper.CL),
                width = .4) +
  labs(y = "dorsal to ventral filament length (per image)") +
  theme_classic()

plot_tnncrisp_3dpf_length
```

```
ggsave(plot_tnncrisp_3dpf_length,
       filename = "plots/tnncrisp_3dpf_length.tiff",
       height = 4, width = 5)
```

# TNN at 1dpf

Load the TNN 1dpf data file

Note that there is one observation from fish29 mislabeled as mut. It should be het

tnn1dpf <- read_csv("csvs_MJ/1dpf_tnn_periodicity.csv")

Fix the genotype on the one incorrect observation

```
tnn1dpf <- tnn1dpf |>
  mutate(genotype = if_else(fish == "fish29", "het", genotype))
```

Create unique fish id and image id

```
tnn1dpf <- tnn1dpf |>
  mutate(fish_id = paste(fish, date, genotype, sep = "_"),
         image_id = paste(fish_id, image, sep = "_image"))
```

Put genotype in order: WT, HET, MUT

```
tnn1dpf <- tnn1dpf |>
  mutate(genotype = factor(genotype, levels = c("wt", "het", "mut")))
```

## 1. Analyze period

Period is the distance between nerve exits, so if the mutation is disrupting nerve placement, there may be a difference in average period.

Include random effect of image and of larva

```
tnn1dpf |>
  ggplot(aes(period)) +
  geom_density()
```

```
tnn1dpf |>
  ggplot(aes(period, linetype = genotype)) +
  geom_density()
```

The mut has a higher period

Ugly but try some LMMs anyway. I’ll try one with a random effect of both image and fish and a second with just the random effect of fish to see if including the image is important.

```
per1mod1 <- lmer(period ~ genotype + (1|image_id) + (1|fish_id),
                   data = tnn1dpf)

per1mod2 <- lmer(period ~ genotype + (1|fish_id),
                   data = tnn1dpf)

anova(per1mod1, per1mod2)
Data: tnn1dpf
Models:
per1mod2: period ~ genotype + (1 | fish_id)
per1mod1: period ~ genotype + (1 | image_id) + (1 | fish_id)
         npar    AIC    BIC  logLik -2*log(L)  Chisq Df Pr(>Chisq)
per1mod2    5 3406.8 3427.3 -1698.4    3396.8
per1mod1    6 3345.2 3369.8 -1666.6    3333.2 63.588  1  1.534e-15 ***
---
Signif. codes:  0 '***' 0.001 '**' 0.01 '*' 0.05 '.' 0.1 ' ' 1
```

The better model includes the random effect of image and fish. Take a look at that model

```
anova(per1mod1)
Type III Analysis of Variance Table with Satterthwaite's method
         Sum Sq Mean Sq NumDF  DenDF F value Pr(>F)
genotype 146.14  73.068     2 116.92  0.9296 0.3976
summary(per1mod1)
Linear mixed model fit by REML. t-tests use Satterthwaite's method [
lmerModLmerTest]
Formula: period ~ genotype + (1 | image_id) + (1 | fish_id)
   Data: tnn1dpf

REML criterion at convergence: 3325.6

Scaled residuals:
    Min      1Q  Median      3Q     Max
-4.2860 -0.2483  0.0935  0.4557  2.2856

Random effects:
 Groups   Name        Variance  Std.Dev.
 image_id (Intercept) 4.462e+01 6.679e+00
 fish_id  (Intercept) 1.225e-12 1.107e-06
 Residual             7.860e+01 8.866e+00
Number of obs: 445, groups:  image_id, 112; fish_id, 56

Fixed effects:
            Estimate Std. Error       df t value Pr(>|t|)
(Intercept)  59.9376     1.5262 115.6531  39.272   <2e-16 ***
genotypehet  -0.8499     1.8178 114.8136  -0.468    0.641
genotypemut   2.0806     2.4534 118.3747   0.848    0.398
---
Signif. codes:  0 '***' 0.001 '**' 0.01 '*' 0.05 '.' 0.1 ' ' 1

Correlation of Fixed Effects:
            (Intr) gntyph
genotypehet -0.840
genotypemut -0.622  0.522
optimizer (nloptwrap) convergence code: 0 (OK)
boundary (singular) fit: see help('isSingular')
```

mut has a slightly higher average period, but the difference is not statistically significant

Retrieve the emmeans

```
emmeans::emmeans(per1mod1, specs = "genotype")
 genotype emmean    SE   df lower.CL upper.CL
 wt         59.9 1.530 53.5     56.9     63.0
 het        59.1 0.988 52.1     57.1     61.1
 mut        62.0 1.920 55.4     58.2     65.9

Degrees-of-freedom method: kenward-roger
Confidence level used: 0.95
pairs(emmeans::emmeans(per1mod1, specs = "genotype"))
 contrast  estimate   SE   df t.ratio p.value
 wt - het      0.85 1.82 53.0   0.467  0.8868
 wt - mut     -2.08 2.45 54.6  -0.848  0.6750
 het - mut    -2.93 2.16 54.7  -1.357  0.3706

Degrees-of-freedom method: kenward-roger
P value adjustment: tukey method for comparing a family of 3 estimates
```

### Plot

Show the average for each fish as the raw data and the emmeans with 95% CI as the summary stat

First generate the average period for each fish

```
fish1 <- tnn1dpf |>
  group_by(genotype, fish_id) |>
  summarize(avg_per = mean(period, na.rm = T),
            .groups = "drop")
```

Save the emmeans as a dataset

```
per1mod1_emm <- emmeans::emmeans(per1mod1, specs = "genotype") |>
  as_tibble()
```

Now plot

```
plot_tnn_1dpf_period <- fish1 |>
  ggplot(aes(genotype, avg_per)) +
  geom_jitter(width = .2,
              color = "gray60") +
  geom_crossbar(data = per1mod1_emm,
                aes(genotype, y = emmean,
                    ymin = lower.CL,
                    ymax = upper.CL),
                width = .4) +
  labs(y = "average periodicty (per larvae)") +
  theme_classic()

plot_tnn_1dpf_period
```

```
ggsave(plot_tnn_1dpf_period,
       filename = "plots/tnn_1dpf_period.tiff",
       height = 4, width = 5)
```

## 2. Analyze variance of period

Because we might see a difference in the variance of the period, we can analyze that directly.

First create the variance of the period for each image

```
var_per1 <- tnn1dpf |>
  group_by(genotype, fish_id, image_id) |>
  summarize(var_per = var(period, na.rm = T),
            .groups = "drop")
```

See these variances

```
var_per1 |>
  ggplot(aes(var_per, linetype = genotype)) +
  geom_density()
```

```
var_per1 |>
  ggplot(aes(genotype, var_per)) +
  geom_jitter(width = .2)
```

The het has higher variance in the period than the other two

Then model using gamma regression

```
var1mod2 <- glmer(var_per ~ genotype + (1|fish_id),
              data = var_per1,
              family = Gamma(link = "log"))
```

The gamma model fits so much better than the log-normal. The failure to converge message is a false positive.

```
summary(var1mod2)
Generalized linear mixed model fit by maximum likelihood (Laplace
  Approximation) [glmerMod]
 Family: Gamma  ( log )
Formula: var_per ~ genotype + (1 | fish_id)
   Data: var_per1

      AIC       BIC    logLik -2*log(L)  df.resid
   1098.0    1111.6    -544.0    1088.0       107

Scaled residuals:
    Min      1Q  Median      3Q     Max
-0.9549 -0.6263 -0.2278  0.3356  2.2567

Random effects:
 Groups  Name        Variance Std.Dev.
 fish_id (Intercept) 1.111    1.054
Number of obs: 112, groups:  fish_id, 56

Fixed effects:
             Estimate Std. Error t value Pr(>|z|)
(Intercept)  3.261755   0.003538   921.9   <2e-16 ***
genotypehet  0.411077   0.003543   116.0   <2e-16 ***
genotypemut -0.700113   0.003543  -197.6   <2e-16 ***
---
Signif. codes:  0 '***' 0.001 '**' 0.01 '*' 0.05 '.' 0.1 ' ' 1

Correlation of Fixed Effects:
            (Intr) gntyph
genotypehet 0.000
genotypemut 0.000  0.000
optimizer (Nelder_Mead) convergence code: 0 (OK)
Model failed to converge with max|grad| = 0.0199581 (tol = 0.002, component 1)
  See ?lme4::convergence and ?lme4::troubleshooting.
```

See the predicted variance for each genotype

```
emmeans::emmeans(var1mod2, specs = "genotype", type = "response")
 genotype response     SE  df asymp.LCL asymp.UCL
 wt           26.1 0.0923 Inf      25.9      26.3
 het          39.4 0.1970 Inf      39.0      39.8
 mut          13.0 0.0649 Inf      12.8      13.1

Confidence level used: 0.95
Intervals are back-transformed from the log scale
pairs(emmeans::emmeans(var1mod2, specs = "genotype"))
 contrast  estimate      SE  df  z.ratio p.value
 wt - het    -0.411 0.00354 Inf -116.037 <0.0001
 wt - mut     0.700 0.00354 Inf  197.610 <0.0001
 het - mut    1.111 0.00501 Inf  221.785 <0.0001

Results are given on the log (not the response) scale.
P value adjustment: tukey method for comparing a family of 3 estimates
```

All pairwise comparisons are significant.

### Plot

First generate the variance of the period for each fish

```
fishvar1 <- tnn1dpf |>
  group_by(fish_id) |>
  fill(ectopic, .direction = "updown") |>
  ungroup() |>
  group_by(genotype, fish_id, ectopic) |>
  summarize(var_per = var(period, na.rm = T),
            .groups = "drop") |>
  mutate(ectopic = if_else(is.na(ectopic), 0, ectopic),
         ectopic = factor(ectopic))
```

Save the emmeans as a dataset

```
var1mod2_emm <- emmeans::emmeans(var1mod2, specs = "genotype", type = "response") |>
  as_tibble()
```

Now plot

```
plot_tnn_1dpf_variance <- fishvar1 |>
  ggplot(aes(genotype, var_per)) +
  geom_jitter(width = .2,
              color = "gray60",
              aes(shape = ectopic)) +
  geom_crossbar(data = var1mod2_emm,
                aes(genotype, y = response,
                    ymin = asymp.LCL,
                    ymax = asymp.UCL),
                width = .4) +
  labs(y = "variance of the periodicty (per larvae)") +
  theme_classic() +
  theme(legend.position = "none")

plot_tnn_1dpf_variance
```

```
ggsave(plot_tnn_1dpf_variance,
       filename = "plots/tnn_1dpf_variance.tiff",
       height = 4, width = 5)
```

# Why does TNN at 2dpf have high SE in the variance analysis?

```
# 2dpf TNN
var_per |>
  ggplot(aes(genotype, log(var_per))) +
  geom_boxplot()
```

```
vcov(varmod1)
3 x 3 Matrix of class "dpoMatrix"
            (Intercept) genotypehet genotypemut
(Intercept)   0.1443379  -0.1433474  -0.1431413
genotypehet  -0.1433474   0.2057438   0.1432942
genotypemut  -0.1431413   0.1432942   0.2754533
# 3dpf TNN
var_per3 |>
  mutate(genotype = factor(genotype, levels = c("wt", "het", "mut"))) |>
  ggplot(aes(genotype, log(var_per))) +
  geom_boxplot()
```

```
vcov(var3mod2)
3 x 3 Matrix of class "dpoMatrix"
              (Intercept)   genotypehet   genotypemut
(Intercept)  2.208176e-05 -1.496312e-07 -1.879063e-08
genotypehet -1.496312e-07  2.215026e-05 -1.537136e-08
genotypemut -1.879063e-08 -1.537136e-08  2.241258e-05
# 3dpf TNC
var_per3tnc|>
  ggplot(aes(genotype, log(var_per))) +
  geom_boxplot()
```

```
# 3dpf TNN crisp
var_per3crisp |>
  ggplot(aes(genotype, log(var_per))) +
  geom_boxplot()
```

```
# 1dpf TNN
var_per1 |>
  ggplot(aes(genotype, log(var_per))) +
  geom_boxplot()
```

For the TNN 2dpf, to explain its higher SE:

- The medians are close
- The IQRs overlap a lot
- No clean separation between groups

For a Gamma(log) model, we estimate a ratio of means. When the bulk of the data overlap like this, many different ratios fit almost equally well, creating a flat likelihood for the group contrast, leading to large SE

# ADDED ANALYSES BASED ON REVIEWER COMMENTS

# TNN gRNA1 at 2dpf

Load the TNN gRNA1 2dpf data file.

grna1 <- read_csv("csvs_MJ/tnn_gRNA1_crisp_cas9_x_period.csv")

Create unique fish id and image id

```
grna1 <- grna1 |>
  mutate(fish_id = paste(fish, date, genotype, sep = "_"),
         image_id = paste(fish_id, image, sep = "_image"))
```

Counts

```
grna1 |>
  group_by(genotype) |>
  summarize(n_fish = n_distinct(fish_id),
            n_images = n_distinct(image_id),
            n_nerve = n())
# A tibble: 2 × 4
  genotype n_fish n_images n_nerve
  <chr>     <int>    <int>   <int>
1 cas9         29       58     238
2 crispant     20       40     175
```

## 1. Analyze period

Period is the distance between nerve exits, so if the mutation is disrupting nerve placement, there may be a difference in average period.

Include random effect of image and of larva

```
grna1 |>
  ggplot(aes(period)) +
  geom_density()
```

```
grna1 |>
  ggplot(aes(period, linetype = genotype)) +
  geom_density()
```

They look quite different

Ugly but try some LMMs anyway. I’ll try one with a random effect of both image and fish and a second with just the random effect of fish to see if including the image is important.

```
grna1mod1 <- lmer(period ~ genotype + (1|image_id) + (1|fish_id),
                   data = grna1)

grna1mod2 <- lmer(period ~ genotype + (1|fish_id),
                   data = grna1)

anova(grna1mod1, grna1mod2)
Data: grna1
Models:
grna1mod2: period ~ genotype + (1 | fish_id)
grna1mod1: period ~ genotype + (1 | image_id) + (1 | fish_id)
          npar    AIC    BIC  logLik -2*log(L)  Chisq Df Pr(>Chisq)
grna1mod2    4 2460.6 2475.6 -1226.3    2452.6
grna1mod1    5 2442.8 2461.5 -1216.4    2432.8 19.844  1  8.403e-06 ***
---
Signif. codes:  0 '***' 0.001 '**' 0.01 '*' 0.05 '.' 0.1 ' ' 1
```

The better model includes the random effect of image and fish. Take a look at that model

```
anova(grna1mod1)
Type III Analysis of Variance Table with Satterthwaite's method
         Sum Sq Mean Sq NumDF  DenDF F value   Pr(>F)
genotype 1039.9  1039.9     1 49.716    10.3 0.002331 **
---
Signif. codes:  0 '***' 0.001 '**' 0.01 '*' 0.05 '.' 0.1 ' ' 1
summary(grna1mod1)
Linear mixed model fit by REML. t-tests use Satterthwaite's method [
lmerModLmerTest]
Formula: period ~ genotype + (1 | image_id) + (1 | fish_id)
   Data: grna1

REML criterion at convergence: 2428

Scaled residuals:
    Min      1Q  Median      3Q     Max
-4.4726 -0.2359  0.0883  0.4776  2.2889

Random effects:
 Groups   Name        Variance Std.Dev.
 image_id (Intercept)  39.321   6.271
 fish_id  (Intercept)   6.423   2.534
 Residual             100.961  10.048
Number of obs: 315, groups:  image_id, 98; fish_id, 49

Fixed effects:
                 Estimate Std. Error     df t value Pr(>|t|)
(Intercept)        73.935      1.211 51.287  61.070  < 2e-16 ***
genotypecrispant   -6.034      1.880 49.716  -3.209  0.00233 **
---
Signif. codes:  0 '***' 0.001 '**' 0.01 '*' 0.05 '.' 0.1 ' ' 1

Correlation of Fixed Effects:
            (Intr)
gntypcrspnt -0.644
```

There is a significant difference by genotype

Retrieve the emmeans

```
emmeans::emmeans(grna1mod1, specs = "genotype")
 genotype emmean   SE   df lower.CL upper.CL
 cas9       73.9 1.21 48.0     71.5     76.4
 crispant   67.9 1.44 45.4     65.0     70.8

Degrees-of-freedom method: kenward-roger
Confidence level used: 0.95
pairs(emmeans::emmeans(grna1mod1, specs = "genotype"))
 contrast        estimate   SE   df t.ratio p.value
 cas9 - crispant     6.03 1.88 46.5   3.209  0.0024

Degrees-of-freedom method: kenward-roger
```

The crispant has significantly shorter period than the cas9

### Plot

Show the average for each fish as the raw data and the emmeans with 95% CI as the summary stat

First generate the average period for each fish

```
sumsgrna1 <- grna1 |>
  group_by(genotype, fish_id) |>
  summarize(avg_per = mean(period, na.rm = T),
            .groups = "drop")
```

Save the emmeans as a dataset

```
predsgrna1 <- emmeans::emmeans(grna1mod1, specs = "genotype") |>
  as_tibble()
```

Now plot

```
plot_grna1_period <- sumsgrna1 |>
  ggplot(aes(genotype, avg_per)) +
  geom_jitter(width = .2,
              color = "gray60") +
  geom_crossbar(data = predsgrna1,
                aes(genotype, y = emmean,
                    ymin = lower.CL,
                    ymax = upper.CL),
                width = .4) +
  labs(y = "average periodicty (per larvae)") +
  theme_classic()

plot_grna1_period
```

```
ggsave(plot_grna1_period,
       filename = "plots/tnn_grna1_2dpf_period.tiff",
       height = 4, width = 5)
```

## 2. Analyze variance of period

Because we see a difference in the variance of the period, we can analyze that directly.

First create the variance of the period for each image

```
var_pergrna1 <- grna1 |>
  group_by(genotype, fish_id, image_id) |>
  summarize(var_per = var(period, na.rm = T),
            .groups = "drop")
```

See these variances

```
var_pergrna1 |>
  ggplot(aes(var_per, linetype = genotype)) +
  geom_density()
```

```
var_pergrna1 |>
  ggplot(aes(genotype, var_per)) +
  geom_jitter(width = .2)
```

The crispant has slightly higher variance

How many images and fish per genotype?

```
# number of images per genotype
var_pergrna1 |>
  count(genotype)
# A tibble: 2 × 2
  genotype     n
  <chr>    <int>
1 cas9        58
2 crispant    40
# number of fish per genotype
var_pergrna1 |>
  group_by(genotype) |>
  summarize(n_fish = n_distinct(fish_id))
# A tibble: 2 × 2
  genotype n_fish
  <chr>     <int>
1 cas9         29
2 crispant     20
```

Then model using log-normal and gamma regression

```
grna1_var_mod1 <- glmer(var_per ~ genotype + (1|fish_id),
              data = var_pergrna1,
              family = gaussian(link = "log"))

grna1_var_mod2 <- glmer(var_per ~ genotype + (1|fish_id),
              data = var_pergrna1,
              family = Gamma(link = "log"))
```

I researched this failure to converge message and it seems that this one is a false positive. We can use this model and it fits so much better than the log-normal

Check the fit of the log-normal and the fit of the Gamma

performance::check_predictions(grna1_var_mod1)

performance::check_predictions(grna1_var_mod2)

See that the Gamma fits the raw data so much better than the log-normal. The Gamma is by far the better model!

```
summary(grna1_var_mod2)
Generalized linear mixed model fit by maximum likelihood (Laplace
  Approximation) [glmerMod]
 Family: Gamma  ( log )
Formula: var_per ~ genotype + (1 | fish_id)
   Data: var_pergrna1

      AIC       BIC    logLik -2*log(L)  df.resid
   1002.1    1012.4    -497.0     994.1        94

Scaled residuals:
    Min      1Q  Median      3Q     Max
-0.9378 -0.6688 -0.2674  0.3539  2.4165

Random effects:
 Groups  Name        Variance Std.Dev.
 fish_id (Intercept) 0.8531   0.9237
Number of obs: 98, groups:  fish_id, 49

Fixed effects:
                 Estimate Std. Error t value Pr(>|z|)
(Intercept)      3.483984   0.005449  639.35   <2e-16 ***
genotypecrispant 0.406052   0.005434   74.73   <2e-16 ***
---
Signif. codes:  0 '***' 0.001 '**' 0.01 '*' 0.05 '.' 0.1 ' ' 1

Correlation of Fixed Effects:
            (Intr)
gntypcrspnt 0.002
optimizer (Nelder_Mead) convergence code: 0 (OK)
Model failed to converge with max|grad| = 0.0129907 (tol = 0.002, component 1)
  See ?lme4::convergence and ?lme4::troubleshooting.
```

See the predicted variance for each genotype

```
emmeans::emmeans(grna1_var_mod2, specs = "genotype", type = "response")
 genotype response    SE  df asymp.LCL asymp.UCL
 cas9         32.6 0.178 Inf      32.2      32.9
 crispant     48.9 0.377 Inf      48.2      49.7

Confidence level used: 0.95
Intervals are back-transformed from the log scale
pairs(emmeans::emmeans(grna1_var_mod2, specs = "genotype"))
 contrast        estimate      SE  df z.ratio p.value
 cas9 - crispant   -0.406 0.00543 Inf -74.727 <0.0001

Results are given on the log (not the response) scale.
```

Crispant has higher variance

### Plot

First generate the variance of the period for each fish

```
grna1_varsum <- grna1 |>
  group_by(fish_id) |>
  fill(ectopic, .direction = "updown") |>
  ungroup() |>
  group_by(genotype, fish_id, ectopic) |>
  summarize(var_per = var(period, na.rm = T),
            .groups = "drop") |>
  mutate(ectopic = if_else(is.na(ectopic), 0, ectopic),
         ectopic = factor(ectopic))
```

Save the emmeans as a dataset

```
grna1_var_emm <- emmeans::emmeans(grna1_var_mod2, specs = "genotype", type = "response") |>
  as_tibble()
```

Now plot

```
plot_grna1_variance <- grna1_varsum |>
  ggplot(aes(genotype, var_per)) +
  geom_jitter(width = .2,
              color = "gray60",
              aes(shape = ectopic)) +
  geom_crossbar(data = grna1_var_emm,
                aes(genotype, y = response,
                    ymin = asymp.LCL,
                    ymax = asymp.UCL),
                width = .4) +
  labs(y = "variance of the periodicty (per larvae)") +
  theme_classic() +
  theme(legend.position = "none")

plot_grna1_variance
```

```
ggsave(plot_grna1_variance,
       filename = "plots/tnn_grna1_2dpf_variance.tiff",
       height = 4, width = 5)
```

# TNN gRNA2 at 2dpf

Load the TNN gRNA2 2dpf data file.

grna2 <- read_csv("csvs_MJ/tnn_gRNA2_cas9_x_period_2dpf.csv")

Create unique fish id and image id

```
grna2 <- grna2 |>
  mutate(fish_id = paste(fish, date, genotype, sep = "_"),
         image_id = paste(fish_id, image, sep = "_image"))
```

Counts

```
grna2 |>
  group_by(genotype) |>
  summarize(n_fish = n_distinct(fish_id),
            n_images = n_distinct(image_id),
            n_nerve = n())
# A tibble: 2 × 4
  genotype n_fish n_images n_nerve
  <chr>     <int>    <int>   <int>
1 cas9         22       44     187
2 crispant     17       34     148
```

## 1. Analyze period

Period is the distance between nerve exits, so if the mutation is disrupting nerve placement, there may be a difference in average period.

Include random effect of image and of larva

```
grna2 |>
  ggplot(aes(period)) +
  geom_density()
```

```
grna2 |>
  ggplot(aes(period, linetype = genotype)) +
  geom_density()
```

They look quite similar

Ugly but try some LMMs anyway. I’ll try one with a random effect of both image and fish and a second with just the random effect of fish to see if including the image is important.

```
grna2mod1 <- lmer(period ~ genotype + (1|image_id) + (1|fish_id),
                   data = grna2)

grna2mod2 <- lmer(period ~ genotype + (1|fish_id),
                   data = grna2)

anova(grna2mod1, grna2mod2)
Data: grna2
Models:
grna2mod2: period ~ genotype + (1 | fish_id)
grna2mod1: period ~ genotype + (1 | image_id) + (1 | fish_id)
          npar    AIC    BIC  logLik -2*log(L)  Chisq Df Pr(>Chisq)
grna2mod2    4 2112.6 2126.8 -1052.3    2104.6
grna2mod1    5 2110.3 2128.1 -1050.2    2100.3 4.2455  1    0.03936 *
---
Signif. codes:  0 '***' 0.001 '**' 0.01 '*' 0.05 '.' 0.1 ' ' 1
```

The better model includes the random effect of image and fish. Take a look at that model

```
anova(grna2mod1)
Type III Analysis of Variance Table with Satterthwaite's method
         Sum Sq Mean Sq NumDF  DenDF F value Pr(>F)
genotype 139.94  139.94     1 37.978  0.8609 0.3593
summary(grna2mod1)
Linear mixed model fit by REML. t-tests use Satterthwaite's method [
lmerModLmerTest]
Formula: period ~ genotype + (1 | image_id) + (1 | fish_id)
   Data: grna2

REML criterion at convergence: 2093.8

Scaled residuals:
    Min      1Q  Median      3Q     Max
-3.3030 -0.2575  0.1502  0.5596  1.9913

Random effects:
 Groups   Name        Variance Std.Dev.
 image_id (Intercept)  28.76    5.363
 fish_id  (Intercept)  43.27    6.578
 Residual             162.55   12.750
Number of obs: 257, groups:  image_id, 78; fish_id, 39

Fixed effects:
                 Estimate Std. Error     df t value Pr(>|t|)
(Intercept)        71.102      1.941 38.332  36.625   <2e-16 ***
genotypecrispant   -2.722      2.934 37.978  -0.928    0.359
---
Signif. codes:  0 '***' 0.001 '**' 0.01 '*' 0.05 '.' 0.1 ' ' 1

Correlation of Fixed Effects:
            (Intr)
gntypcrspnt -0.662
```

There is no significant difference by genotype

Retrieve the emmeans

```
emmeans::emmeans(grna2mod1, specs = "genotype")
 genotype emmean   SE   df lower.CL upper.CL
 cas9       71.1 1.94 37.2     67.2     75.0
 crispant   68.4 2.20 36.6     63.9     72.8

Degrees-of-freedom method: kenward-roger
Confidence level used: 0.95
pairs(emmeans::emmeans(grna2mod1, specs = "genotype"))
 contrast        estimate   SE   df t.ratio p.value
 cas9 - crispant     2.72 2.93 36.9   0.928  0.3596

Degrees-of-freedom method: kenward-roger
```

The crispant has very slightly shorter period than the cas9

### Plot

Show the average for each fish as the raw data and the emmeans with 95% CI as the summary stat

First generate the average period for each fish

```
sumsgrna2 <- grna2 |>
  group_by(genotype, fish_id) |>
  summarize(avg_per = mean(period, na.rm = T),
            .groups = "drop")
```

Save the emmeans as a dataset

```
predsgrna2 <- emmeans::emmeans(grna2mod1, specs = "genotype") |>
  as_tibble()
```

Now plot

```
plot_grna2_period <- sumsgrna2 |>
  ggplot(aes(genotype, avg_per)) +
  geom_jitter(width = .2,
              color = "gray60") +
  geom_crossbar(data = predsgrna2,
                aes(genotype, y = emmean,
                    ymin = lower.CL,
                    ymax = upper.CL),
                width = .4) +
  labs(y = "average periodicty (per larvae)") +
  theme_classic()

plot_grna2_period
```

```
ggsave(plot_grna2_period,
       filename = "plots/tnn_grna2_2dpf_period.tiff",
       height = 4, width = 5)
```

## 2. Analyze variance of period

Because we see a difference in the variance of the period, we can analyze that directly.

First create the variance of the period for each image

```
var_pergrna2 <- grna2 |>
  group_by(genotype, fish_id, image_id) |>
  summarize(var_per = var(period, na.rm = T),
            .groups = "drop")
```

See these variances

```
var_pergrna2 |>
  ggplot(aes(var_per, linetype = genotype)) +
  geom_density()
```

```
var_pergrna2 |>
  ggplot(aes(genotype, var_per)) +
  geom_jitter(width = .2)
```

The crispant has similar variance

How many images and fish per genotype?

```
# number of images per genotype
var_pergrna2 |>
  count(genotype)
# A tibble: 2 × 2
  genotype     n
  <chr>    <int>
1 cas9        44
2 crispant    34
# number of fish per genotype
var_pergrna2 |>
  group_by(genotype) |>
  summarize(n_fish = n_distinct(fish_id))
# A tibble: 2 × 2
  genotype n_fish
  <chr>     <int>
1 cas9         22
2 crispant     17
```

Then model using log-normal and gamma regression

```
grna2_var_mod1 <- glmer(var_per ~ genotype + (1|fish_id),
              data = var_pergrna2,
              family = gaussian(link = "log"))

grna2_var_mod2 <- glmer(var_per ~ genotype + (1|fish_id),
              data = var_pergrna2,
              family = Gamma(link = "log"))
```

Check the fit of the log-normal and the fit of the Gamma

```
performance::check_predictions(grna2_var_mod1)
Warning: Maximum value of original data is not included in the
  replicated data.
  Model may not capture the variation of the data.
```

performance::check_predictions(grna2_var_mod2)

See that the Gamma fits the raw data so much better than the log-normal. The Gamma is by far the better model!

```
summary(grna2_var_mod2)
Generalized linear mixed model fit by maximum likelihood (Laplace
  Approximation) [glmerMod]
 Family: Gamma  ( log )
Formula: var_per ~ genotype + (1 | fish_id)
   Data: var_pergrna2

      AIC       BIC    logLik -2*log(L)  df.resid
    898.0     907.4    -445.0     890.0        74

Scaled residuals:
    Min      1Q  Median      3Q     Max
-1.0318 -0.6587 -0.1457  0.4181  1.7782

Random effects:
 Groups  Name        Variance Std.Dev.
 fish_id (Intercept) 1.781    1.335
Number of obs: 78, groups:  fish_id, 39

Fixed effects:
                 Estimate Std. Error t value Pr(>|z|)
(Intercept)       4.08564    0.31582  12.937   <2e-16 ***
genotypecrispant  0.09638    0.47823   0.202     0.84
---
Signif. codes:  0 '***' 0.001 '**' 0.01 '*' 0.05 '.' 0.1 ' ' 1

Correlation of Fixed Effects:
            (Intr)
gntypcrspnt -0.655
```

See the predicted variance for each genotype

```
emmeans::emmeans(grna2_var_mod2, specs = "genotype", type = "response")
 genotype response   SE  df asymp.LCL asymp.UCL
 cas9         59.5 18.8 Inf      32.0       110
 crispant     65.5 23.7 Inf      32.3       133

Confidence level used: 0.95
Intervals are back-transformed from the log scale
pairs(emmeans::emmeans(grna2_var_mod2, specs = "genotype"))
 contrast        estimate    SE  df z.ratio p.value
 cas9 - crispant  -0.0964 0.478 Inf  -0.202  0.8403

Results are given on the log (not the response) scale.
```

The difference in variance of period is not statistically significant between cas9 and crispant in the TNN gRNA2 fish at 2dpf

### Plot

First generate the variance of the period for each fish

```
grna2_varsum <- grna2 |>
  group_by(fish_id) |>
  fill(ectopic, .direction = "updown") |>
  ungroup() |>
  group_by(genotype, fish_id, ectopic) |>
  summarize(var_per = var(period, na.rm = T),
            .groups = "drop") |>
  mutate(ectopic = if_else(is.na(ectopic), 0, ectopic),
         ectopic = factor(ectopic))
```

Save the emmeans as a dataset

```
grna2_var_emm <- emmeans::emmeans(grna2_var_mod2, specs = "genotype", type = "response") |>
  as_tibble()
```

Now plot

```
plot_grna2_variance <- grna2_varsum |>
  ggplot(aes(genotype, var_per)) +
  geom_jitter(width = .2,
              color = "gray60",
              aes(shape = ectopic)) +
  geom_crossbar(data = grna2_var_emm,
                aes(genotype, y = response,
                    ymin = asymp.LCL,
                    ymax = asymp.UCL),
                width = .4) +
  labs(y = "variance of the periodicty (per larvae)") +
  theme_classic() +
  theme(legend.position = "none")

plot_grna2_variance
```

```
ggsave(plot_grna2_variance,
       filename = "plots/tnn_grna2_2dpf_variance.tiff",
       height = 4, width = 5)
```

# TNC 94 Injection TNN at 2dpf

Load the TNC injection TNN 2dpf data file.

tnc94 <- read_csv("csvs_MJ/tnc94_injection_tnn_muthet_2dpf.csv")

Create unique fish id and image id

```
tnc94 <- tnc94 |>
  mutate(fish_id = paste(fish, date, genotype, sep = "_"),
         image_id = paste(fish_id, image, sep = "_image"))
```

Counts

```
tnc94 |>
  group_by(genotype) |>
  summarize(n_fish = n_distinct(fish_id),
            n_images = n_distinct(image_id),
            n_nerve = n())
# A tibble: 2 × 4
  genotype  n_fish n_images n_nerve
  <chr>      <int>    <int>   <int>
1 het_crisp     13       26     109
2 mut_crisp      8       16      65
```

## 1. Analyze period

Period is the distance between nerve exits, so if the mutation is disrupting nerve placement, there may be a difference in average period.

Include random effect of image and of larva

```
tnc94 |>
  ggplot(aes(period)) +
  geom_density()
```

```
tnc94 |>
  ggplot(aes(period, linetype = genotype)) +
  geom_density()
```

They look quite similar

Ugly but try some LMMs anyway. I’ll try one with a random effect of both image and fish and a second with just the random effect of fish to see if including the image is important.

```
tnc94mod1 <- lmer(period ~ genotype + (1|image_id) + (1|fish_id),
                   data = tnc94)

tnc94mod2 <- lmer(period ~ genotype + (1|fish_id),
                   data = tnc94)

anova(tnc94mod1, tnc94mod2)
Data: tnc94
Models:
tnc94mod2: period ~ genotype + (1 | fish_id)
tnc94mod1: period ~ genotype + (1 | image_id) + (1 | fish_id)
          npar    AIC    BIC  logLik -2*log(L)  Chisq Df Pr(>Chisq)
tnc94mod2    4 1024.9 1036.5 -508.47    1016.9
tnc94mod1    5 1021.4 1035.8 -505.70    1011.4 5.5443  1    0.01854 *
---
Signif. codes:  0 '***' 0.001 '**' 0.01 '*' 0.05 '.' 0.1 ' ' 1
```

The better model includes the random effect of image and fish. Take a look at that model

```
anova(tnc94mod1)
Type III Analysis of Variance Table with Satterthwaite's method
         Sum Sq Mean Sq NumDF  DenDF F value Pr(>F)
genotype 13.012  13.012     1 19.068  0.1317 0.7207
summary(tnc94mod1)
Linear mixed model fit by REML. t-tests use Satterthwaite's method [
lmerModLmerTest]
Formula: period ~ genotype + (1 | image_id) + (1 | fish_id)
   Data: tnc94

REML criterion at convergence: 1005.2

Scaled residuals:
    Min      1Q  Median      3Q     Max
-4.3181 -0.2633  0.0791  0.4754  2.2866

Random effects:
 Groups   Name        Variance Std.Dev.
 image_id (Intercept) 30.946   5.563
 fish_id  (Intercept)  6.552   2.560
 Residual             98.812   9.940
Number of obs: 132, groups:  image_id, 42; fish_id, 21

Fixed effects:
                  Estimate Std. Error     df t value Pr(>|t|)
(Intercept)         67.906      1.704 18.657  39.858   <2e-16 ***
genotypemut_crisp    1.008      2.777 19.068   0.363    0.721
---
Signif. codes:  0 '***' 0.001 '**' 0.01 '*' 0.05 '.' 0.1 ' ' 1

Correlation of Fixed Effects:
            (Intr)
gntypmt_crs -0.614
```

There is no significant difference by genotype

Retrieve the emmeans

```
emmeans::emmeans(tnc94mod1, specs = "genotype")
 genotype  emmean   SE   df lower.CL upper.CL
 het_crisp   67.9 1.70 18.7     64.3     71.5
 mut_crisp   68.9 2.19 19.4     64.3     73.5

Degrees-of-freedom method: kenward-roger
Confidence level used: 0.95
pairs(emmeans::emmeans(tnc94mod1, specs = "genotype"))
 contrast              estimate   SE   df t.ratio p.value
 het_crisp - mut_crisp    -1.01 2.78 19.1  -0.363  0.7209

Degrees-of-freedom method: kenward-roger
```

The het_crisp and mut_crisp are very similar

### Plot

Show the average for each fish as the raw data and the emmeans with 95% CI as the summary stat

First generate the average period for each fish

```
sumstnc94 <- tnc94 |>
  group_by(genotype, fish_id) |>
  summarize(avg_per = mean(period, na.rm = T),
            .groups = "drop")
```

Save the emmeans as a dataset

```
predstnc94 <- emmeans::emmeans(tnc94mod1, specs = "genotype") |>
  as_tibble()
```

Now plot

```
plot_tnc94_period <- sumstnc94 |>
  ggplot(aes(genotype, avg_per)) +
  geom_jitter(width = .2,
              color = "gray60") +
  geom_crossbar(data = predstnc94,
                aes(genotype, y = emmean,
                    ymin = lower.CL,
                    ymax = upper.CL),
                width = .4) +
  labs(y = "average periodicty (per larvae)") +
  theme_classic()

plot_tnc94_period
```

```
ggsave(plot_tnc94_period,
       filename = "plots/tnc94_injecttnn_2dpf_period.tiff",
       height = 4, width = 5)
```

## 2. Analyze variance of period

Because we see a difference in the variance of the period, we can analyze that directly.

First create the variance of the period for each image

```
var_pertnc94 <- tnc94 |>
  group_by(genotype, fish_id, image_id) |>
  summarize(var_per = var(period, na.rm = T),
            .groups = "drop")
```

See these variances

```
var_pertnc94 |>
  ggplot(aes(var_per, linetype = genotype)) +
  geom_density()
```

```
var_pertnc94 |>
  ggplot(aes(genotype, var_per)) +
  geom_jitter(width = .2)
```

The mut_crisp is shifted slightly higher, but the het_crisp has several high outliers

How many images and fish per genotype?

```
# number of images per genotype
var_pertnc94 |>
  count(genotype)
# A tibble: 2 × 2
  genotype      n
  <chr>     <int>
1 het_crisp    26
2 mut_crisp    16
# number of fish per genotype
var_pertnc94 |>
  group_by(genotype) |>
  summarize(n_fish = n_distinct(fish_id))
# A tibble: 2 × 2
  genotype  n_fish
  <chr>      <int>
1 het_crisp     13
2 mut_crisp      8
```

Then model using log-normal and gamma regression

```
tnc94_var_mod1 <- glmer(var_per ~ genotype + (1|fish_id),
              data = var_pertnc94,
              family = gaussian(link = "log"))

tnc94_var_mod2 <- glmer(var_per ~ genotype + (1|fish_id),
              data = var_pertnc94,
              family = Gamma(link = "log"))
```

Check the fit of the log-normal and the fit of the Gamma

```
performance::check_predictions(tnc94_var_mod1)
Warning: Maximum value of original data is not included in the
  replicated data.
  Model may not capture the variation of the data.
```

```
performance::check_predictions(tnc94_var_mod2)
Warning: Minimum value of original data is not included in the
  replicated data.
  Model may not capture the variation of the data.
```

See that the Gamma fits the raw data so much better than the log-normal. The Gamma is by far the better model!

```
summary(tnc94_var_mod2)
Generalized linear mixed model fit by maximum likelihood (Laplace
  Approximation) [glmerMod]
 Family: Gamma  ( log )
Formula: var_per ~ genotype + (1 | fish_id)
   Data: var_pertnc94

      AIC       BIC    logLik -2*log(L)  df.resid
      420       427      -206       412        38

Scaled residuals:
    Min      1Q  Median      3Q     Max
-0.9215 -0.7038 -0.2468  0.4215  2.1288

Random effects:
 Groups  Name        Variance Std.Dev.
 fish_id (Intercept) 1.274    1.129
Number of obs: 42, groups:  fish_id, 21

Fixed effects:
                  Estimate Std. Error t value Pr(>|z|)
(Intercept)         3.6689     0.3967   9.248   <2e-16 ***
genotypemut_crisp  -0.7272     0.6249  -1.164    0.245
---
Signif. codes:  0 '***' 0.001 '**' 0.01 '*' 0.05 '.' 0.1 ' ' 1

Correlation of Fixed Effects:
            (Intr)
gntypmt_crs -0.605
```

See the predicted variance for each genotype

```
emmeans::emmeans(tnc94_var_mod2, specs = "genotype", type = "response")
 genotype  response    SE  df asymp.LCL asymp.UCL
 het_crisp     39.2 15.60 Inf     18.02      85.3
 mut_crisp     18.9  9.43 Inf      7.14      50.3

Confidence level used: 0.95
Intervals are back-transformed from the log scale
pairs(emmeans::emmeans(tnc94_var_mod2, specs = "genotype"))
 contrast              estimate    SE  df z.ratio p.value
 het_crisp - mut_crisp    0.727 0.625 Inf   1.164  0.2445

Results are given on the log (not the response) scale.
```

het_crisp has slightly higher variance of period, but the difference is not statistically significant

### Plot

First generate the variance of the period for each fish

```
tnc94_varsum <- tnc94 |>
  group_by(fish_id) |>
  fill(ectopic, .direction = "updown") |>
  ungroup() |>
  group_by(genotype, fish_id, ectopic) |>
  summarize(var_per = var(period, na.rm = T),
            .groups = "drop") |>
  mutate(ectopic = if_else(is.na(ectopic), 0, ectopic),
         ectopic = factor(ectopic))
```

Save the emmeans as a dataset

```
tnc94_var_emm <- emmeans::emmeans(tnc94_var_mod2, specs = "genotype", type = "response") |>
  as_tibble()
```

Now plot

```
plot_tnc94_variance <- tnc94_varsum |>
  ggplot(aes(genotype, var_per)) +
  geom_jitter(width = .2,
              color = "gray60",
              aes(shape = ectopic)) +
  geom_crossbar(data = tnc94_var_emm,
                aes(genotype, y = response,
                    ymin = asymp.LCL,
                    ymax = asymp.UCL),
                width = .4) +
  labs(y = "variance of the periodicty (per larvae)") +
  theme_classic() +
  theme(legend.position = "none")

plot_tnc94_variance
```

```
ggsave(plot_tnc94_variance,
       filename = "plots/tnc94_injecttnn_2dpf_variance.tiff",
       height = 4, width = 5)
```
